# Atlas of cell types and regulatory elements underlying human facial diversity

**DOI:** 10.1038/s41588-026-02748-y

**Published:** 2026-09-24

**Authors:** Alek G. Erickson, Yaakov Gershtein, Rozalina Galimullina, Felix Waern, Tonyak Riba, Markéta Kaiser, Daniela Schnyder, Lei Li, Nikita Vaulin, Saga Samuelsson, Aliia Murtazina, Sergey Isaev, Thibault Bouderlique, Viktoria Parobkova, Hugo Zeberg, Tomáš Zikmund, Jozef Kaiser, Kaj Fried, Elena I. Shagimardanova, Leyla Shigapova, Guzel R. Gazizova, Ivan V. Reva, Ruslan Deviatiiarov, Kateřina Vymazalová, Seppe Goovaerts, Yuk Kit Lor, Gonçalo Castelo-Branco, Peter Claes, Xiaofei Li, Oleg Gusev, Andrei S. Chagin, Erik Sundström, Peter V. Kharchenko, Igor Adameyko

**Affiliations:** 1 Department of Molecular Biosciences, Wenner-Gren Institute, Stockholm University, Stockholm, Sweden; 2 Department of Physiology and Pharmacology, Karolinska Institutet, Stockholm, Sweden; 3 Department of Neuroimmunology, Center for Brain Research, Medical University of Vienna, Vienna, Austria; 4 Central European Institute of Technology, Brno University of Technology, Brno, Czech Republic; 5 Department of Internal Medicine and Clinical Nutrition, Institute of Medicine, Sahlgrenska Academy, Gothenburg University, Gothenburg, Sweden; 6 Department of Neuroscience, Karolinska Institutet, Stockholm, Sweden; 7 Institute of Fundamental Medicine and Biology, Kazan Federal University, Kazan, Russia; 8 Loginov Moscow Clinical Scientific Center, Moscow, Russia; 9 Life Improvement by Future Technologies (LIFT) Center, Moscow, Russia; 10 Intractable Disease Research Center, Graduate School of Medicine, Juntendo University, Tokyo, Japan; 11 Research Group of Medical Anthropology and Clinical Anatomy, Department of Anatomy, Faculty of Medicine, Masaryk University, Brno, Czech Republic; 12 Department of Human Genetics, KU Leuven, Leuven, Belgium; 13 Division of Molecular Neurobiology, Department of Molecular Biology and Biochemistry, Karolinska Institute, Stockholm, Sweden; 14 Department of Electrical Engineering, Processing of Speech and Images, KU Leuven, Leuven, Belgium; 15 Department of Neurobiology, Care Sciences and Society, Karolinska Institutet, Solna, Sweden; 16 Altos Labs, San Diego Institute of Science, San Diego, CA USA

**Keywords:** Transcriptomics, Stem cells, Anatomy

## Abstract

Human genetic diversity generates an astonishing variety of facial shapes, and craniofacial anomalies rank among the most common birth defects. Identifying the cellular mechanisms that mediate the genome’s influence on facial variation remains a challenge. Here we created a multimodal facial atlas across embryonic weeks 6–11, providing single-cell transcriptomics, chromatin accessibility and spatial transcriptomics, all at single-cell resolution. We characterized 56 cell states, mapping mesenchymal subtypes and their gene–enhancer *cis*-regulatory landscapes in space and time. Gene expression associations with facial traits were strongest in early mesenchymal progenitor cells, gradually becoming more region restricted. Autocorrelation analysis revealed patterning genes that define spatial neighborhoods of mesenchyme, potentially explaining trait specificity of their nearby variants. Enhancers of key pathology-related genes were found to be likely vehicles for generating facial variation in modern human populations. One such enhancer, linked to *PAX1* expression, appears to be important for normal skeletal development in mice. Finally, facial effects inferred from the genome-wide association study, cell signaling interaction analysis and validation in mice revealed that peripheral nerves fine-tune maxilla shape during embryonic development. Together, these data offer new insights into the mechanisms underlying human phenotypic individuality and yield a multimodal atlas useful for studying craniofacial development and abnormalities.

## Abstract (web summary)

Single-cell transcriptomics, spatial transcriptomics and chromatin accessibility maps of the human craniofacial region in developing embryos provide insights into the mechanisms underlying facial variation and pathology.

## Main

The human species shows an astonishing tapestry of facial appearances, each uniquely crafted to fulfill a role in the theater of social interaction^1^. The genetic underpinnings of facial variation are evident through familial resemblance among relatives and the strikingly similar faces of monozygotic twins. While genome-wide association studies (GWASs)^2–9^ shed light on the genetic basis of facial diversity, the cellular processes linking genetic variants to specific facial morphologies remain elusive.

The human face develops primarily from cranial neural crest cells (CNCCs) and mesodermal progenitor cells that give rise to cartilage, bones, muscles, adipose tissue, dermis, vasculature and innervation^10,11^ through a conserved morphogenetic chronology^12–14^. After neural crest migration and differentiation into ectomesenchyme^15^, epithelial signaling centers, such as the frontonasal ectodermal zone^16^, and forebrain^17,18^ induce skeletogenic condensations in a species-specific pattern^19,20^. Skeletogenic condensations give rise to the cartilaginous chondrocranium, which helps define facial dimensions and jaw shape, and their spatial positioning influences overall cranial proportions, including brachycephalic and dolichocephalic aspects^21–25^. Later, the chondrocranium directly either ossifies or serves as a template for dermal bone^17,21,26^. Nearby tissues, such as muscle, adipose tissue and dermis, are also likely to influence facial skeletal shape^27^, in part due to reciprocal mechanical or paracrine interactions^28,29^. However, it remains unclear which cell states represent the major drivers of adult facial variation, given the substantial difference between humans and model organisms.

Here we profile spatiotemporal activities of genes, enhancers and cell types in the developing human face. Using single-cell multi-omics and spatial transcriptomic analysis, we characterize the transcriptional diversification of ectomesenchyme derivatives, map their locations and identify likely molecular regulators. We find that the influence of craniofacial cell types on human morphological variation diminishes over time, with the strongest impacts at the earliest stages of ectomesenchyme development. Integration with GWAS analysis and validation in a mouse model revealed that peripheral nerves regulate shaping of the mammalian maxilla. Using spatial autocorrelation analysis, we identify key genes that appear to contribute to the transcriptional patterning of mesenchymal cell states across facial regions. While these patterning genes appear to be highly related to facial diseases, overlapping with epigenomic data indicates that genetic variation within dedicated enhancers of patterning genes drives individuality in facial shaping. Furthermore, many of the identified candidate enhancers appear to be spatiotemporally regulated and cell-type specific. Targeted deletion of one such identified enhancer sequence, which was linked to expression of *PAX1* in ectomesenchyme progenitors and dermal mesenchyme in humans, led to skeletal defects in mice.

## Results

### Comprehensive single-cell atlas of human craniofacial development

**Fig. 1:**
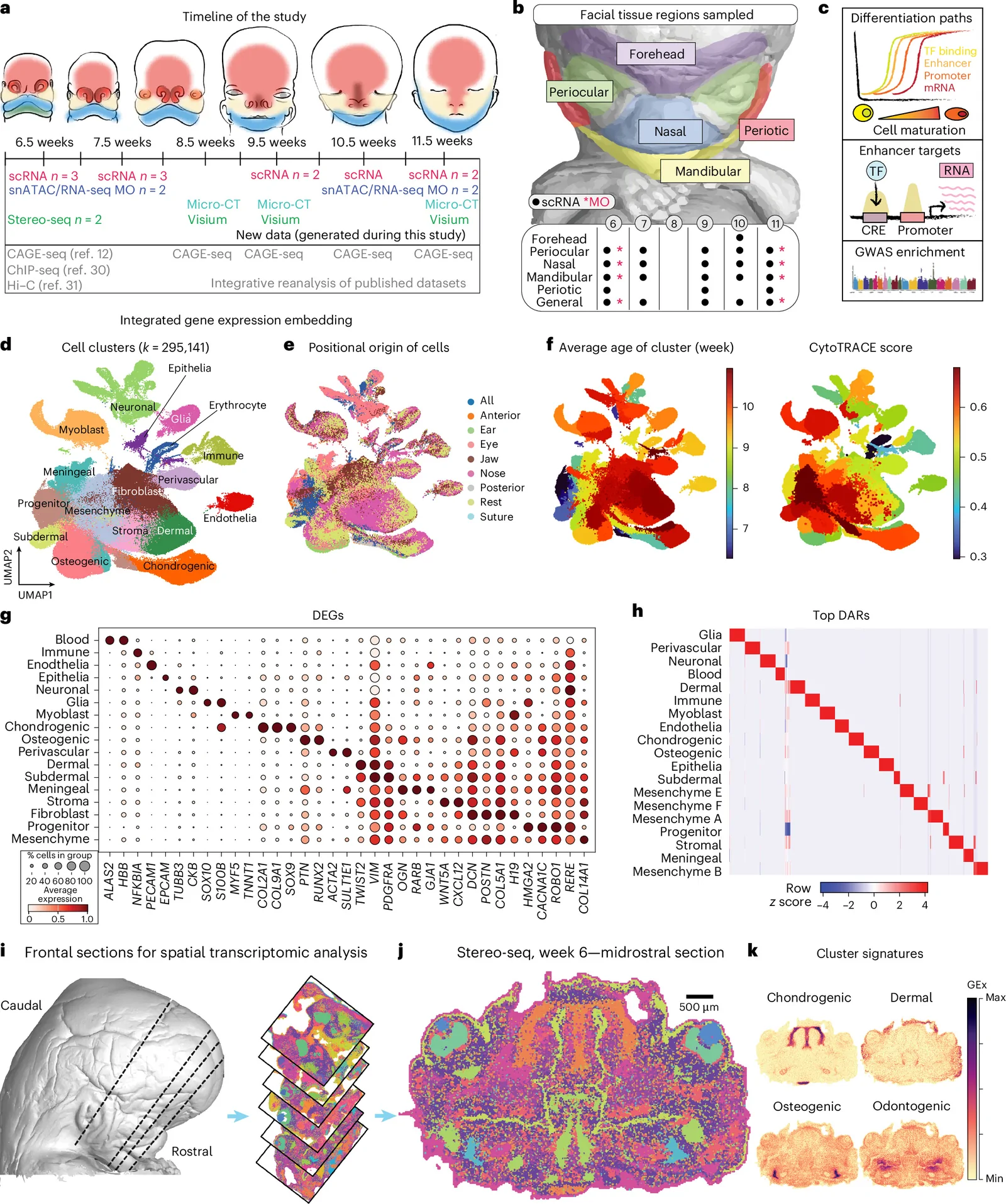
Spatially resolved, multi-omic, single-cell atlas of human craniofacial development. **a**, Above the timeline—cranial regions of human embryos are illustrated at the depicted stages. Frontonasal prominence is shown in pink, maxillary prominence is shown in yellow, mandibular prominence is shown in blue and the second branchial arch is shown in green. Middle: the assay modality used to analyze that particular stage is listed. Summary and ages of all embryos are used to generate datasets for the study and the modalities are used. Bottom: summary of published datasets is re-analyzed. **b**, Top: micro-CT scan of a PCW8 human embryo, anterior region, color coded by the strategy used to subdivide samples into regions. Bottom: breakdown of samples by age and region. Numbers represent embryo age in PCW. Black dots indicate the presence of scRNA-seq data for the given stage–region combination (not including biological replicates); red stars indicate the presence of multi-omics snRNA/ATAC–seq data for the given stage–region combination (not including biological replicates). Overall, for scRNA-seq, *n* = 30 samples were generated from *n* = 11 embryos; for the multi-omics, *n* = 12 samples were generated from four individuals. **c**, Illustration of the general aims of the human developmental atlas study—describe cellular trajectories, link enhancers to gene partners and determine cellular bases for GWAS traits. **d**, UMAP embedding of the combined scRNA-seq and snRNA/ATAC–seq datasets, calculated using gene expression data only, which will be referred to as the ‘general embedding’. The embedding is color coded by, and labeled with, the coarse-grained cell-type annotations (see Extended Data Fig. 2 for the fine-grained annotations). **e**, General embedding, color coded by the facial region each cell was isolated from. **f**, General embedding. Left: fine-grained clusters are color coded by the average age of the embryo the cells were isolated from (in weeks). Right: fine-grained clusters are color coded by their average CytoTRACE score, a measure of transcript diversity in cells (used as a proxy for cellular maturity; Methods). Pearson’s product-moment correlation test (two sided) was used to determine a significant anticorrelation between cluster averages in age and CytoTRACE score (*r* = −0.5867, *P* = 2.015 × 10^−6^); no multiple-comparison adjustment was applied. **g**, Dot plot showing enrichment of a few major cell-type-specific marker genes used to annotate the cell types in the dataset. Dot size indicates the fraction of cells in each cluster expressing the gene, and dot color shows the normalized expression levels. **h**, Heatmap of DARs across cell clusters. For each cluster, the top 40 peaks with the largest absolute log_2_(FC) were selected for visualization. Rows represent annotated cell clusters (coarse grained). Values are row-scaled log_2_(FC), with color indicating relative accessibility (blue = decreased, red = increased). **i**, Micro-CT scan of a PCW8 human embryo, anterior region, marked with dotted lines indicating relative positions of cutting planes used to generate frontal Stereo-seq sections at different rostrocaudal levels (*n* = 8). **j**, Midrostral section of PCW6 human embryo, analyzed by Stereo-seq and color coded by Leiden cluster (see Extended Data Figs. 3 and 4 for characterization of these sections). **k**, Example gene signatures of mesenchymal derivatives (bone, cartilage, dermis and teeth), plotted on the midrostral Stereo-seq section. Red indicates high expression, blue indicates medium expression and gray/white indicates low expression. MO, multi-omics; FC, fold change; DEGs, differentially expressed genes.

We investigated a developmental time series spanning critical periods of human development (from the 6th to the 12th postconceptional week or 6–12 PCW) using a battery of methods including single-cell RNA sequencing (scRNA-seq) transcriptomics, spatial transcriptomics and microcomputed tomography (micro-CT; Fig. 1a). To link gene expression to chromatin regulation, we performed paired measurements of gene expression and chromatin accessibility from single-nuclei RNA (snRNA)/ATAC–seq and further overlapped these with published epigenomic datasets^30–32^. Samples were dissected under a stereo-microscope into subregions and separately processed for scRNA-seq analysis, with resulting datasets being pooled later (Fig. 1b). With these data, we aimed to (1) study cell-type specificity and spatiotemporal activity of candidate *cis*-regulatory elements (referred to as ‘cCREs’), (2) assign putative cCREs to gene targets and (3) link the identified cCREs to facial traits and congenital malformations by overlapping them with GWAS variants (Fig. 1c).

**Extended Data Fig. 1:**
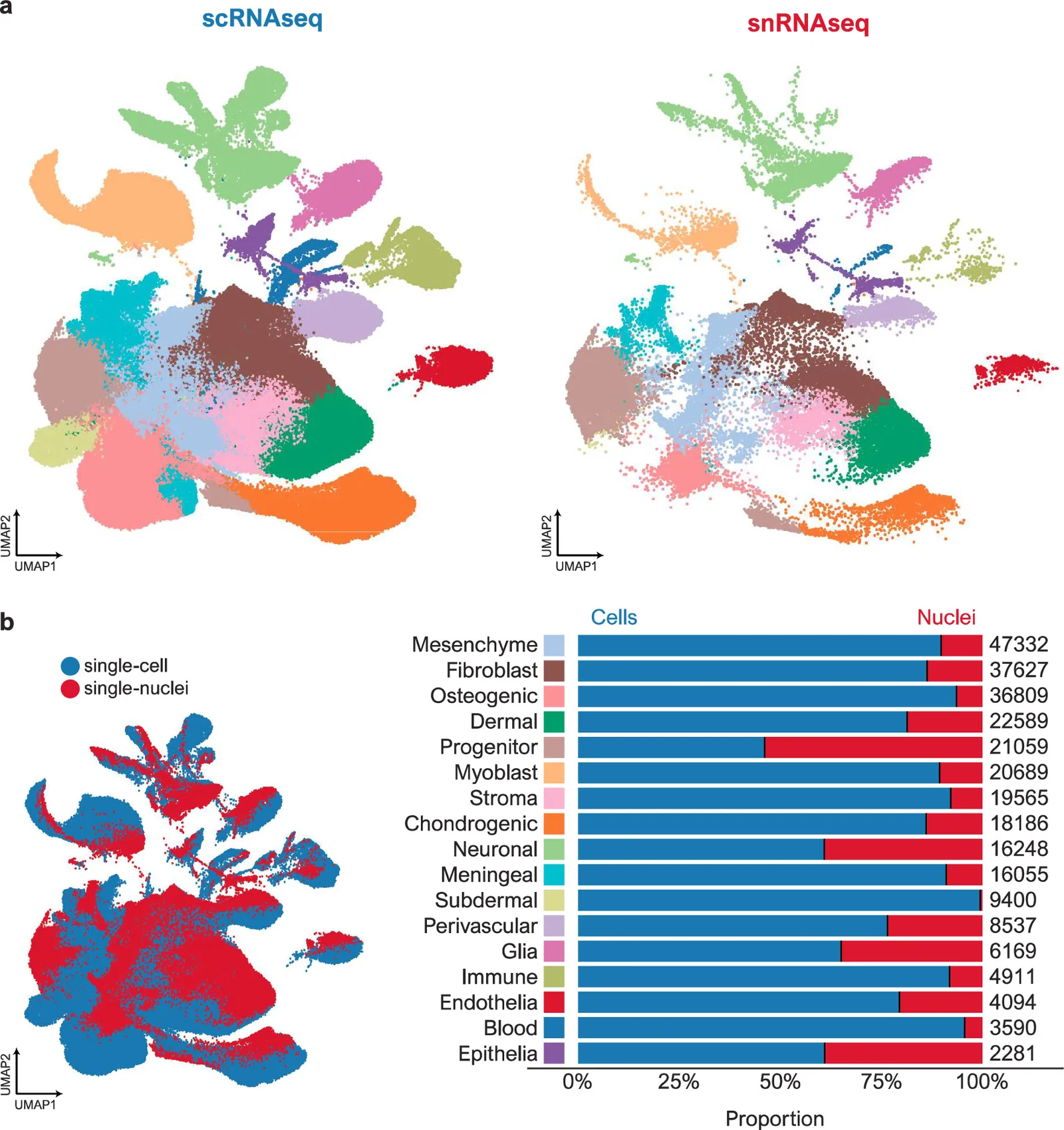
An integrated transcriptomic embedding from facial cells and nuclei. **a**, The general embedding is shown with cellular-only (left) or nuclei-only (right) fractions, color coded by coarse-grained annotations. **b**, Left, UMAP of the embedding color coded by sample type. Right, each coarse-grained cluster annotation is shown next to a stacked bar plot displaying the observed proportion of nuclei versus cells, and the total number of cells/nuclei recovered in each cluster.

**Extended Data Fig. 2:**
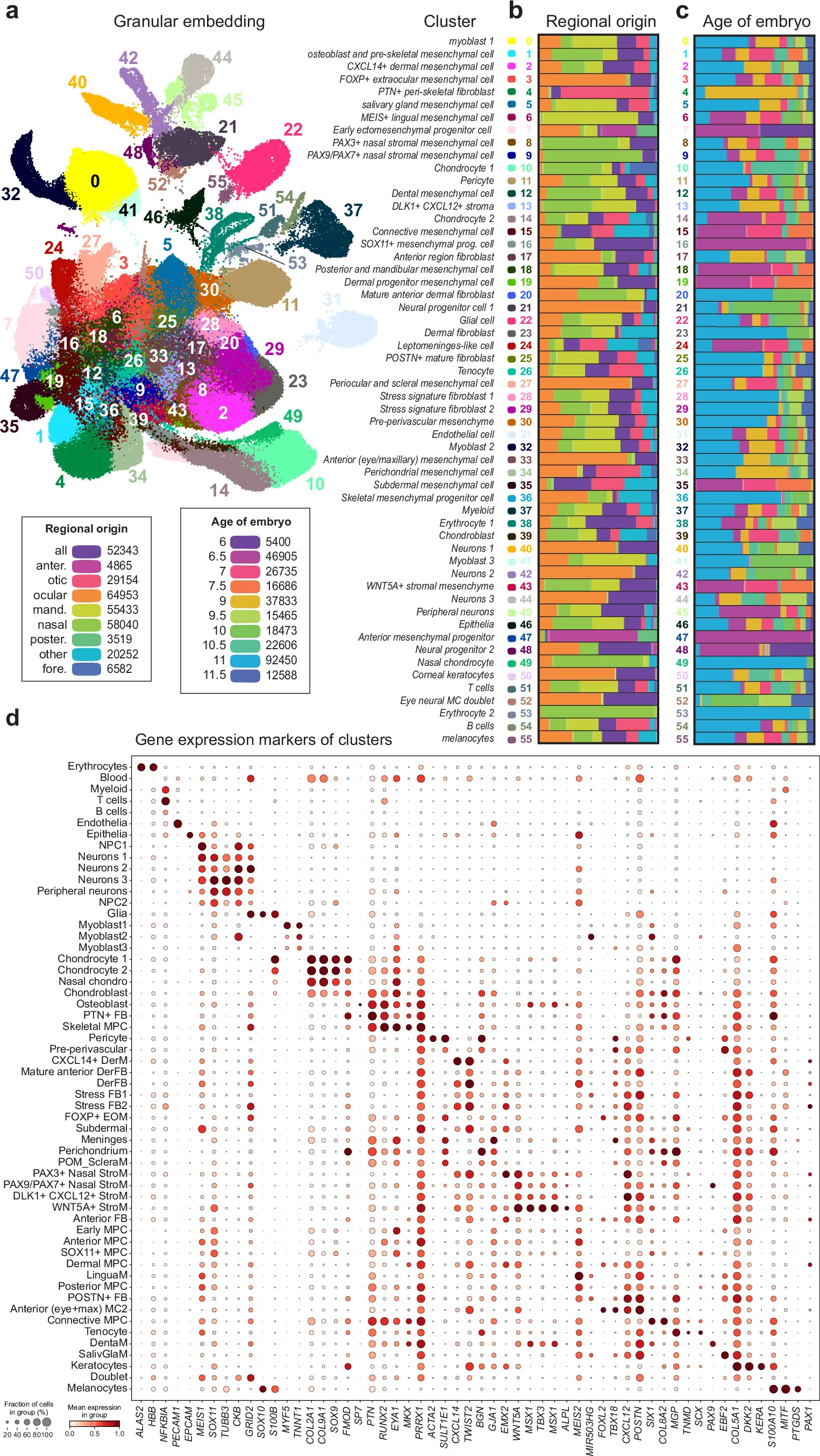
Spatiotemporal distribution of facial cell types and their markers. **a**, Granular cell clustering. The general embedding is shown, color coded and numerically labeled by granular cell-type annotations. Cell-type names are shown in the right. **b**, Stacked bar plots showing each cluster subdivided by facial regions that cells were isolated from. Color key below the embedding as shown in **a** (left). **c**, Stacked bar plots showing each cluster subdivided by age of embryo that cells were isolated from. Color key below the embedding as shown in **a** (right). **d**, Dot plot showing enrichment of some cell-type-specific marker genes used to annotate the granular cell types in the dataset. Dot size indicates the fraction of cells in each cluster expressing the gene, and dot color shows the normalized expression levels.

Uniform manifold approximation projection (UMAP) was used to visualize cell diversity in the dataset (Fig. 1d), their positional origins (Fig. 1e) and maturation stages (Fig. 1f). Both the single-nuclei and single-cell datasets captured predominantly ectomesenchyme derivatives, such as bone, cartilage, pericytes, meninges, dermal fibroblasts and tenocytes, and retrieved many other expected cell types (Extended Data Fig. 1). Using differentially expressed marker genes, we annotated 17 coarse-grain clusters (Fig. 1g) and 56 fine-grain clusters (Extended Data Fig. 2 and Supplementary Table 1), which showed numerous differentially accessible regions (DARs; Fig. 1h, Supplementary Fig. 1 and Supplementary Table 2).

Anatomical regions were unevenly represented in the integrated embedding, which may reflect both compositional effects and position-dependent expression within cell types. While some cell types were transcriptionally compact regardless of origin, most mesenchyme showed origin-dependent enrichment (Fig. 1e) and a left-to-right temporal progression as progenitors differentiated (Fig. 1f). Mean CytoTRACE score negatively correlated with cluster age, indicating a loss of multipotency over time (Fig. 1f). The primary ectomesenchyme derivatives included cartilage, bone, perivascular cells, meninges and dermis (tenocytes and dental clusters did not separate from the mesenchyme supercluster). Chondrocytes emerged first (Fig. 1f, dark blue and cyan region of chondrogenic trajectory), along with meninges-like cells, while other fates developed later, in line with reports that CNCCs engage the chondrogenic program early^33^. Exploration of the underlying transcriptional trajectories identified major phases for both chondrogenesis and myogenesis, and also identified potential positional effects in the mesenchymal cell fate decision hierarchy (Supplementary Note 1, Supplementary Figs. 2–4 and Supplementary Table 2).

### Spatial codes in human embryonic facial tissue

**Extended Data Fig. 3:**
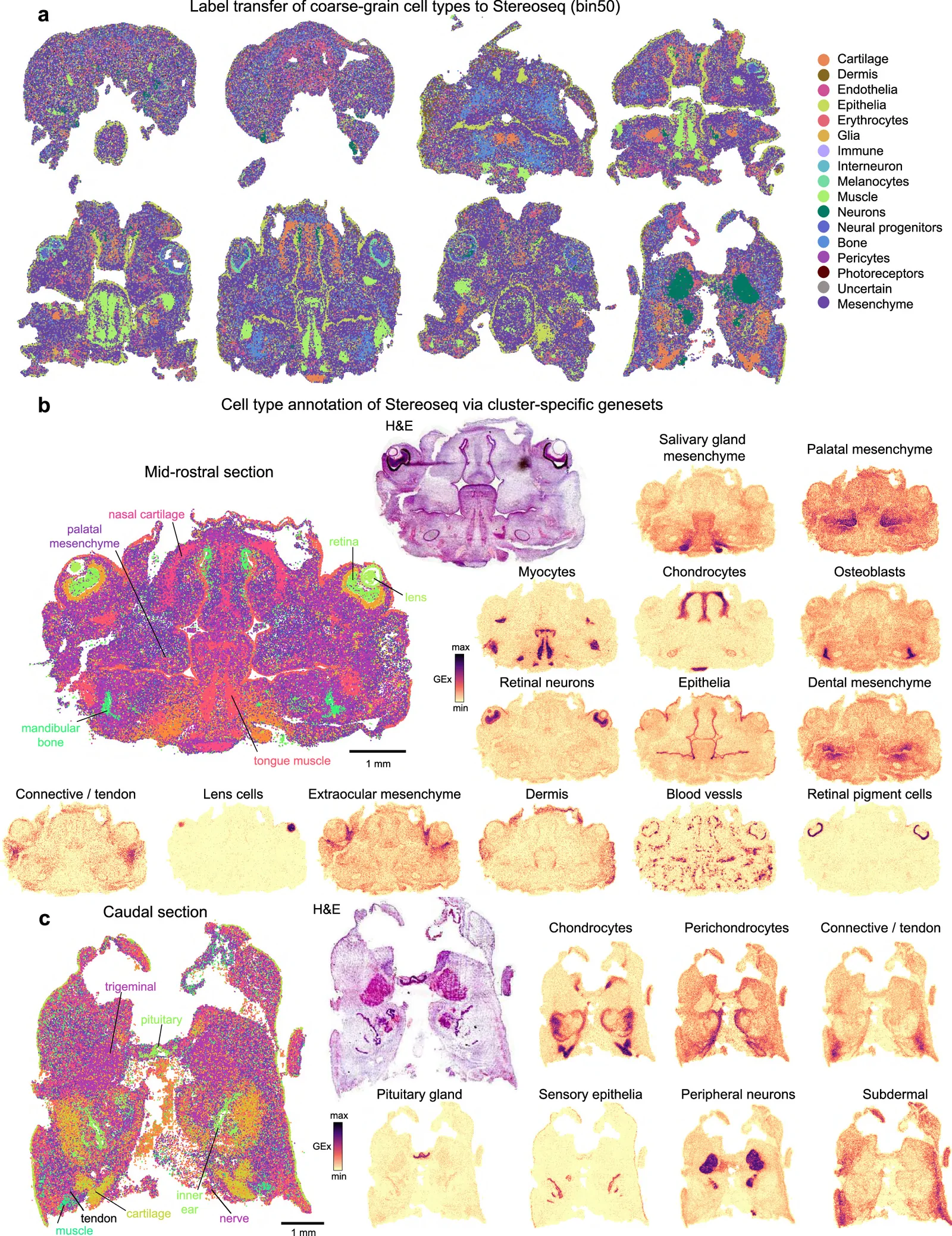
Stereoseq analysis of week 6 embryonic human face tissues. **a**, All eight Stereoseq sections (rendered at bin size 50), color coded by consensus cell-type assignments (labels to the right). **b**, Cell-type annotation using Stereoseq data. Left, the midrostral section is shown color coded according to the Leiden cluster. Hematoxylin and Eosin stain to the upper right. Signatures defining specific clusters are mapped on the right (bin 50), and can be explored in detail via Supplementary Table 1. Darker color indicates greater expression. **c**, Cell-type annotation using Stereoseq data. Left, the caudal section is shown color coded according to the Leiden cluster. Hematoxylin and Eosin stain to the upper right. Signatures defining specific clusters are mapped on the right (bin 50), and can be explored in detail via Supplementary Table 1. Darker color indicates greater expression.

**Extended Data Fig. 4:**
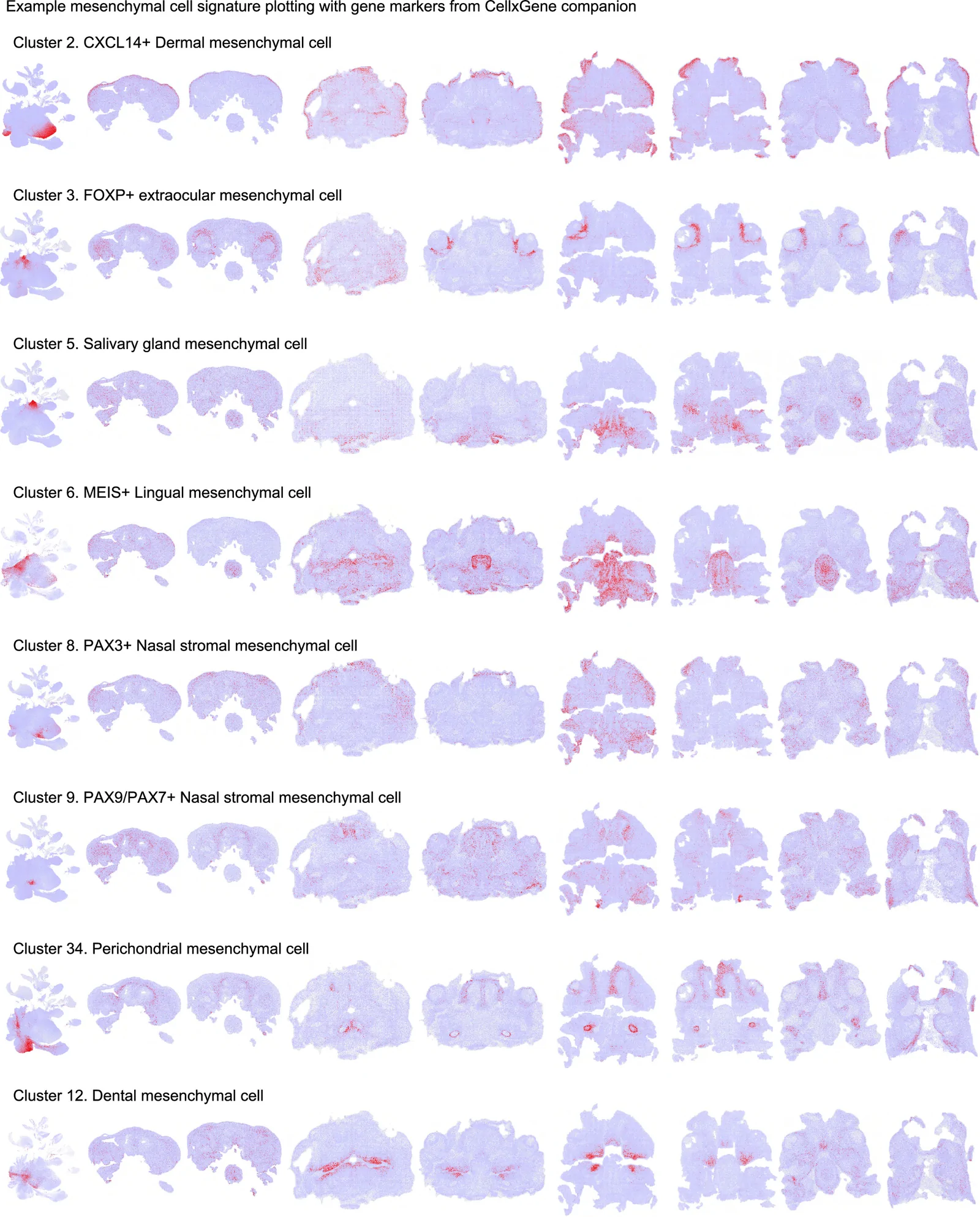
Example mesenchymal signatures. Feature plots using all eight Stereoseq sections to plot average gene expression levels of entire gene signatures (genes used can be found in the Supplementary Table 1).

Annotating facial cell heterogeneity was limited by the noisy, dynamic and overlapping gene expression profiles of mesenchymal subpopulations, which we suspected was caused by sampling cells from different anatomical regions (Fig. 1e and Supplementary Fig. 3). To resolve this, localization of observed cell clusters was achieved by spatial transcriptomics analysis (Stereo-seq^34^, *n* = 8 sections from *n* = 2 week 6.5 embryos) of frontal sections across different rostrocaudal levels (Fig. 1i), resulting in a high-resolution map of facial cells (Fig. 1j and Extended Data Figs. 3 and 4) suitable for visualizing scRNA-seq cluster marker signatures (Fig. 1k and Extended Data Figs. 3 and 4). The 10x Genomics Visium platform was also used to characterize tissues from sagittal and frontal sections of tissue near sensory organs (*n* = 3 embryos; Supplementary Fig. 5); however, due to its comparatively lower resolution, we treated the Visium results as confirmatory and instead primarily relied on the Stereo-seq data to identify cell types in a spatially resolved manner. Together, these data, spanning several craniofacial regions (Supplementary Note 2), facilitated deducing gene signatures that could be used for anatomy-aware categorization of mesenchymal cells, as well as identifying potential regulators of cell positional identity.

**Fig. 2:**
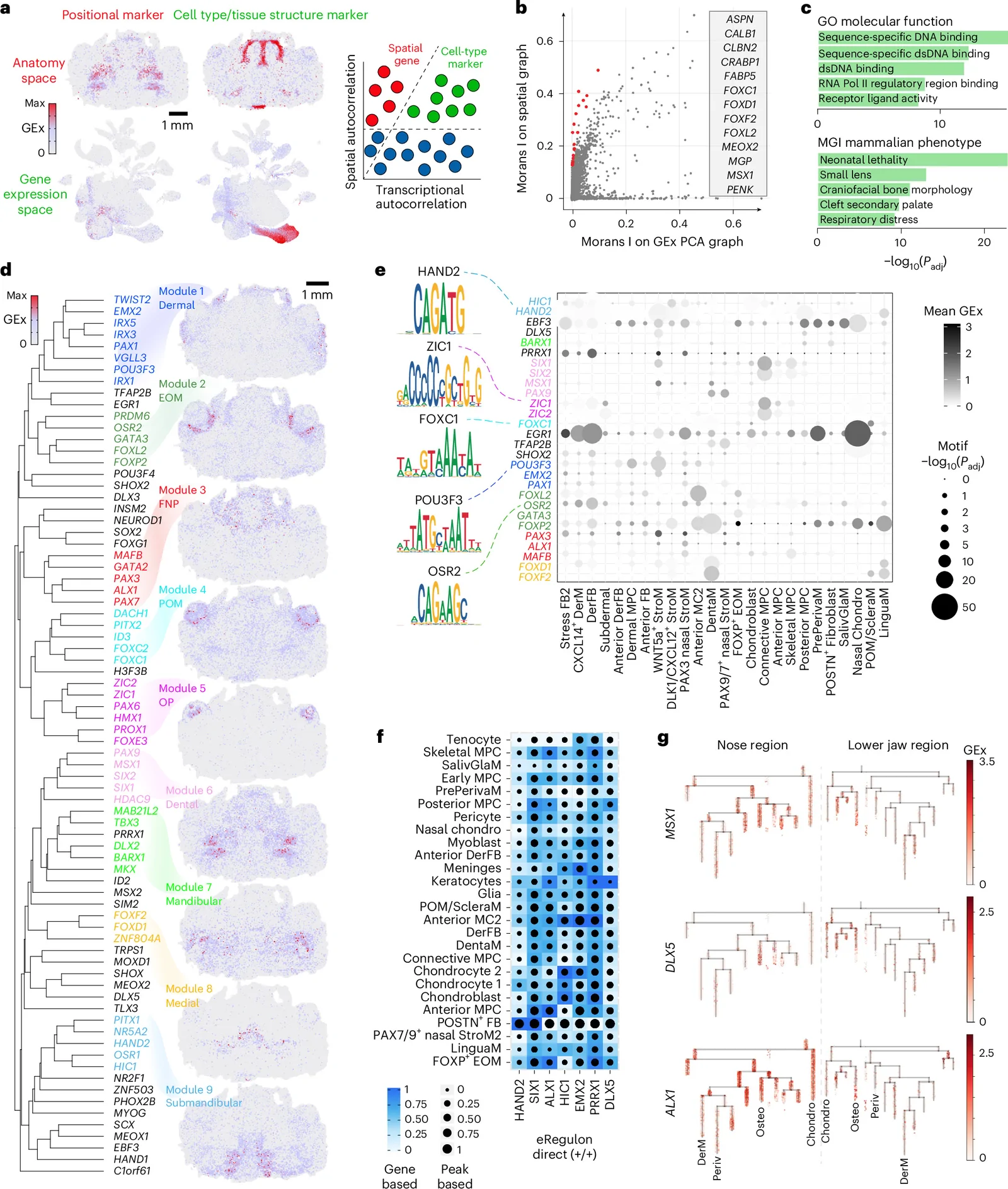
Positional codes in the developing human face. **a**, Unbiased classification of genes according to positional enrichment. Left: midrostral section (top, bin = 50) and general embedding (bottom) color coded with expression levels of *MSX1* (left) and *COL2A1* (right) genes, as representative examples of genes with regional enrichment, and with low (left) or high (right) transcriptional dispersal. Darker red color indicates greater expression. Right: schematic illustration of a scatterplot in which each gene’s anatomical and transcriptional Moran’s I are plotted. **b**, Scatterplot of spatial (*y* axis) and transcriptional (*x* axis) Moran’s I for all genes detected in the midrostral section. Genes with greater spatial value than 0.1 and spatial/GEx ratio of at least 5 were highlighted, of these a few selected gene symbols are shown in the box. Please explore the full gene list in the Supplementary Table 3. **c**, Bar plots showing significance levels of selected results from the top ten hits from a gene set enrichment analysis of spatial genes through Enrichr (GO molecular function 2025, top; MGI mammalian phenotype level 4 2024, bottom). Enrichment was assessed using Fisher’s exact test, with Benjamini–Hochberg correction for multiple testing. **d**, Top-left, hierarchical clustering of spatial TFs (transcriptional regulators found among the list of spatial genes) based on distance in a Pearson correlation matrix reflecting coexpression of genes in a downsampled midrostral section (bin = 500). Genes are color coded by module. Combined module gene signatures are plotted on the midrostral section (bin = 50); darker red color indicates greater expression. **e**, Motif-cell-type dot plot showing TF motif enrichment and expression. Motif enrichment was assessed using a hypergeometric test, with FDR calculated by Benjamini–Hochberg adjustment of *P* values. Motif–cluster pairs were retained if any pair met mean expression of >0.1 and FDR of <0.05. Dot size indicates −log_10_(FDR), and dot color indicates mean expression (white to black, clipped to 0–3). Pairs with FDR ≥ 0.05 were not plotted. **f**, Regulon enrichment calculated by SCENIC+. Color (gene based) refers to the expression level of the genes in the regulon, whereas dot size (peak based) refers to the percentage of cells with AUC above threshold. +/+ indicates that the TF is predicted to both open chromatin and activate gene expression. **g**, Expression of patterning genes *MSX1* (top), *DLX5* (middle) and *ALX1* (bottom), and on a dendrogram embedding generated through scFates for nose (left) and jaw (right) samples. Darker red color indicates greater expression. DerM, dermal mesenchyme; PerivM, perivascular mesenchyme; StroM, stromal mesenchymal cell; osteo; osteogenic mesenchyme; chondro, chondrogenic mesenchyme; GEx, gene expression; POM, periocular mesenchyme; EOM, extraocular mesenchyme; OP, optic progenitor cells; FNP, frontonasal prominence; FB, fibroblast; MPC mesenchymal progenitor cell; MC2, mesenchymal cell 2; FDR, false discovery rate; GO, Gene Ontology; MGI, Mouse Genome Informatics; AUC, area under the curve.

**Extended Data Fig. 5:**
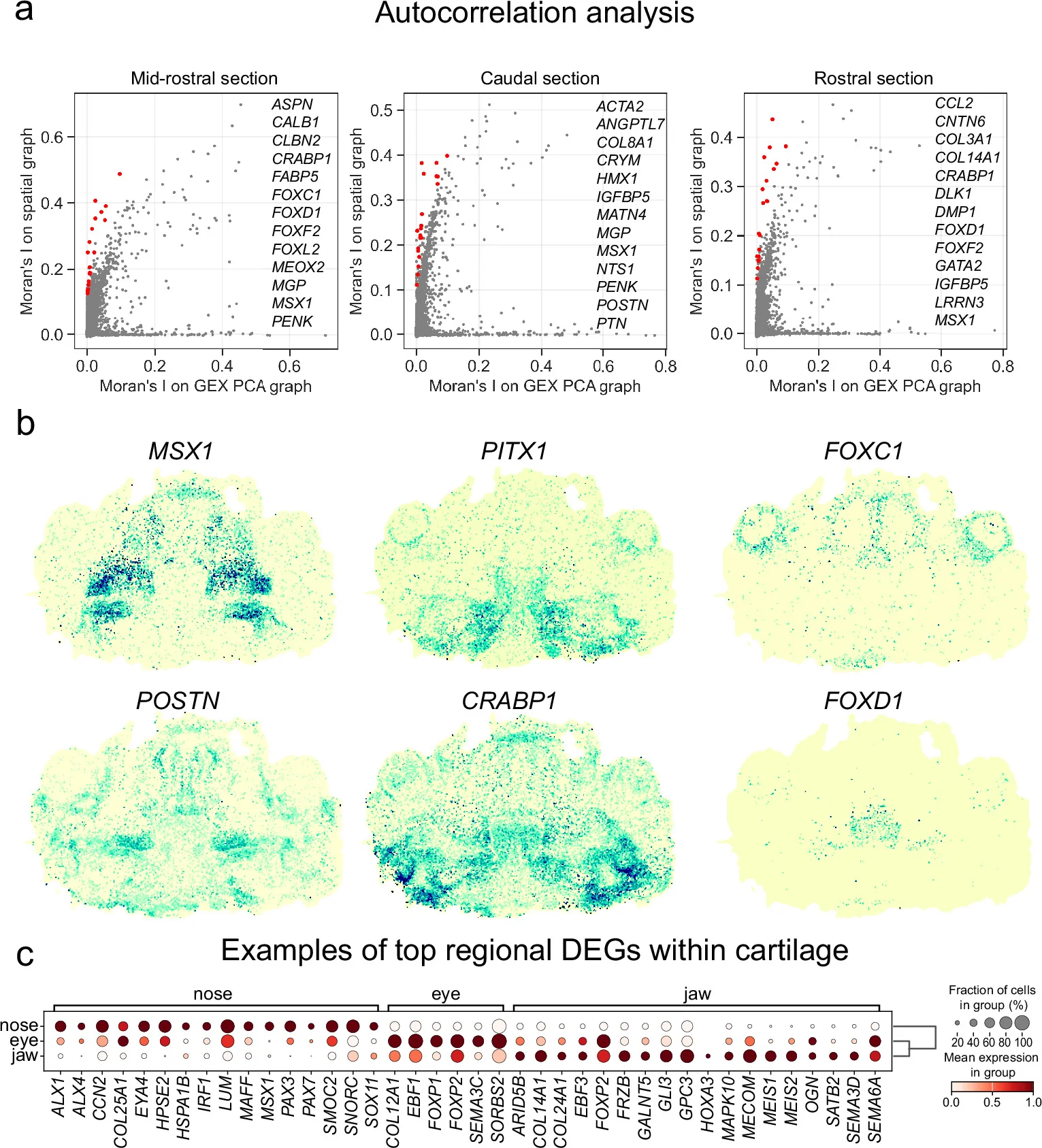
Autocorrelation analysis reveals spatial genes. **a**, Scatterplot of spatial (y-axis) and transcriptional (x-axis) Moran’s I for all genes detected in each of the Stereoseq sections. Genes with a spatial value greater than 0.1 and a spatial/GEX ratio of at least 5 were highlighted; of these, a few selected gene symbols are shown in the box. Explore the full gene list in the Supplementary Table 3. GEx, gene expression; PCA, principal components analysis. **b**, Feature plots showing gene expression of selected spatial genes in the midrostral section (bin 50). Darker color indicates higher expression levels. **c**, Dot plot showing genes that are differentially expressed in chondrocytes originating from different facial regions, compared to other chondrocytes.

Spatially heterogeneous mesenchyme gives rise to various cell types, but the factors driving this cellular patterning process in the embryonic human face are not well understood. The establishment of positional identity in progenitor cells often relies on nested patterns of transcription factor (TF) expression across different cell types, restricted to a specific body location. Thus, to discover genes that are highly associated with specific spatial regions (that is, spatial codes), we used Moran’s I metric to examine autocorrelation of genes in anatomical space^35^, as well as gene expression space such as principal component analysis (PCA) space or UMAP (Fig. 2a). Moran’s I metric asks whether neighboring locations (cells) are likely to have similar or different values for a given parameter (gene expression). Comparing positional versus transcriptional autocorrelation can allow for distinguishing genes that are location specific but not cell-type specific, which is a defining feature of a spatial code such as the Hox code^36^. Application of this approach to the Stereo-seq data revealed 396 so-called ‘spatial’ genes (examples in Fig. 2b and Extended Data Fig. 5, full list in Supplementary Table 3 and further discussion in Supplementary Note 2).

**Extended Data Fig. 6:**
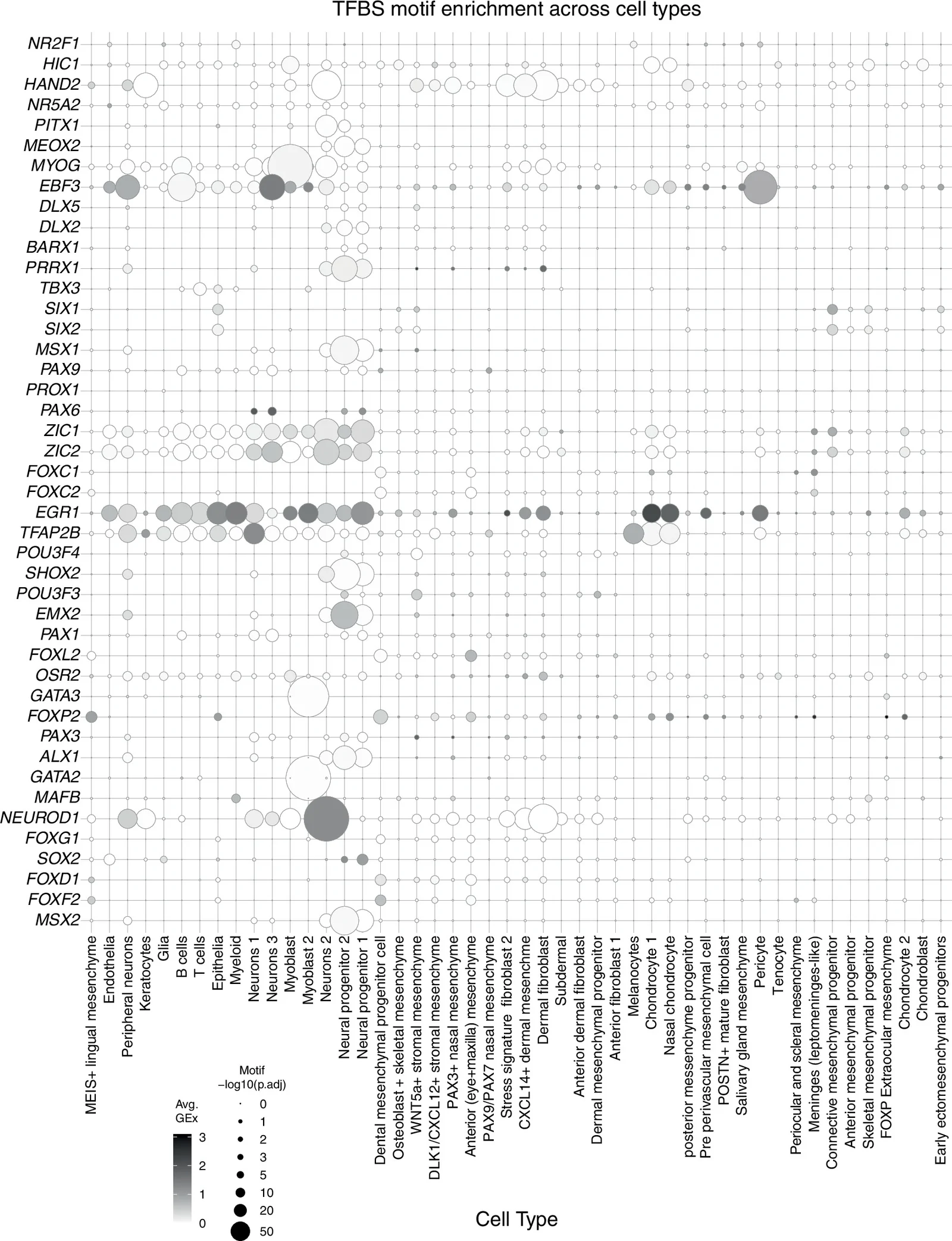
Motif enrichment of spatial transcription factors. Motif-cell-type dot plot showing TF motif enrichment and expression. Motifs were kept if any pair met mean expression > 0.1 and FDR < 0.05. Dot size encodes −log_10_(FDR); dot color encodes mean expression (white to black, clipped to 0–3). FDR = Benjamini–Hochberg-adjusted p-value.

Assessment of molecular function of the spatial gene list through Gene Ontology revealed TF activity as the top functional annotation, and craniofacial diseases were enriched in the top associated phenotypes (Fig. 2c and Supplementary Table 4). Hierarchical clustering based on the expression in downsampled Stereo-seq bins (Methods) revealed nine modules of correlated TFs that mark distinct facial domains, namely predermis, forming eyelids, nasal region, eye region, lens, dental region, lower face, surface of tongue and submandibular region (Fig. 2d). Binding sites for spatial TFs were significantly enriched in DARs for a multitude of mesenchymal cell types, with cell-type specificity partially overlapping per module (Fig. 2e and Extended Data Fig. 6). Enhancer-driven gene regulatory network (eGRN) inference using SCENIC+ revealed widespread activity of spatial TF networks, their candidate enhancers and downstream target genes in mesenchymal clusters (Fig. 2f and Supplementary Fig. 6). In addition, trajectory analysis of mesenchymal differentiation in nose and jaw showed expression of spatial TFs, including genes like *ALX1* and *DLX5* that encode known patterning regulators^37^, were pseudotime associated, with region-specific expression profiles (Fig. 2g and Supplementary Fig. 6). Thus, we identified groups (modules) of coexpressed TFs that are active in various spatial compartments of the early human face. The data suggest that, for each facial region and corresponding mesenchymal cell type, a different module of dedicated TFs influences the site-specific and cell-type-specific differentiation within that region.

### Enhancer screening reveals a new genomic region influencing facial growth

**Fig. 3:**
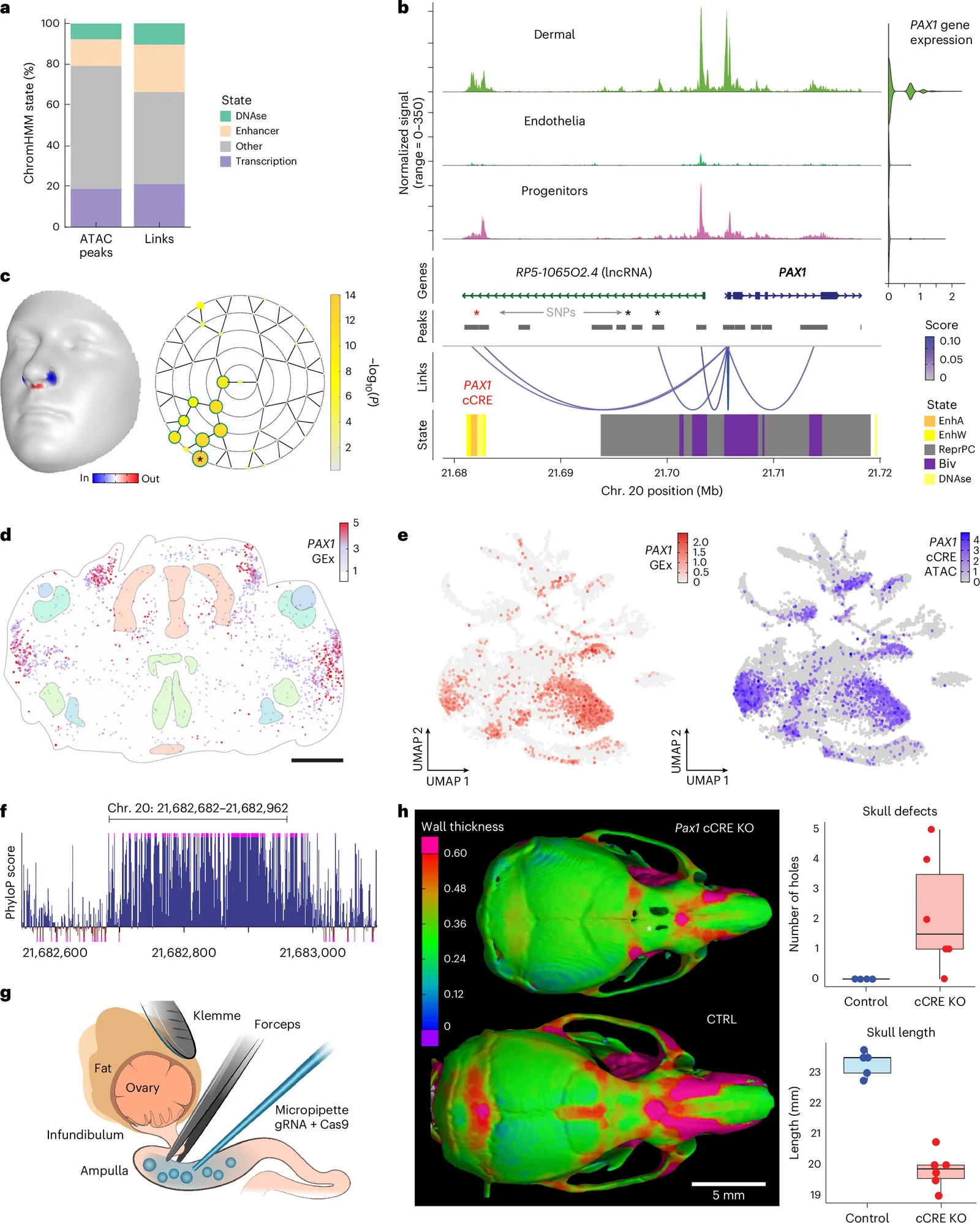
Linking enhancers to partner genes reveals a new driver of facial skeletogenesis. **a**, Stacked bar plot showing the proportions of chromatin states (ChromHMM, 25-state model) observed among ATAC peaks (left) and linked peaks (right). **b**, Chromatin accessibility and enhancer activity across dermal, endothelial and progenitor populations are shown (top; normalized signal). Gene models for *RP5-1065O2.4* and *PAX1* are displayed with SNP locations. cCREs and enhancer state annotations (chromHMM) are shown below, with significant predicted enhancer–gene links (arcs) connecting distal elements to *PAX1*. Right: violin plot showing *PAX1* expression across profiled cell types. **c**, Plot of facial effects for SNP at chr. 20: 21,682,649 (rs6047572). Left: normal map—normal distances between the shapes exaggerated in the direction of both alleles for the indicated facial segment. Red represents an outward protrusion, while blue represents an inward depression. Right: radial dendrogram corresponds to facial segmentsmentioned in ref. ^2^. A darker border indicates genome-wide significance. The asterisk indicates the plotted segment. **d**, Expression of *PAX1* on the midrostral section. Darker red color of dots indicates greater expression levels. The border of the tissue section is outlined. Cartilages, bone, tongue muscles and eyes are highlighted as reference landmarks. Scale bar, 1 mm. **e**, Expression levels of *PAX1* gene (left) and chromatin accessibility of *PAX1*-cCRE peak (right) among nuclei from the general embedding. Darker purple color of dots indicates greater value. Color scales indicate expression/openness values. **f**, Evolutionarily conserved region within the *PAX1*-cCRE. PhyloP scores across vertebrates are shown, with peaks indicating nucleotide-level conservation within a 278 bp portion of the *PAX1*-cCRE. **g**, Schematic of the iGONAD experimental procedure used for generating cCRE-KO mouse strain (Methods). **h**, Micro-CT analysis of the craniofacial skeleton in the *Pax1*-cCRE-edited mouse strain. The 3D reconstructions of skulls show craniofacial wall thickness (color scale) in the *Pax1*-cCRE-edited mice developing small bodies (top), compared to normal control littermates (CTRL, bottom). An asterisk indicates regions of reduced bone formation (apparent cranial defects). Box plots (right) quantify number of skull holes (top) and skull length (bottom) from *n* = 6 mutant and *n* = 5 littermate control mice (each measurement shown as a point; box shows median ± IQR; whiskers show most extreme points within 1.5× IQR). 3D, three dimensional. EnhA, active enhancer; EnhW, weak enhancer; ReprPC, polycomb represesion; Biv, bivalent; DNAse, DNAse accessible.

Spatiotemporal tuning of gene expression through enhancers is a hypothesized vehicle for noncoding variants’ influence on facial shape^38^. We sought to identify CREs (focusing on enhancers) that interact with key patterning genes during human facial development. While enhancer grammar and logic have not been fully elucidated, certain characteristics can be used to find them. First, enhancers physically associate with distant target promoters kilobases away (although exceptions have been noted, up to 1.5 Mbp^39^), driven by DNA looping or by the boundaries of topologically associating domains (TADs)^40^. Hierarchically nested sub-TADs are thought to promote further short-range enhancer interactions. Second, enhancers often display bidirectional transcription of capped enhancer RNAs (eRNA), which is detectable through the cap analysis of gene expression (CAGE)^41^. Third, enhancers show histone marks such as acetylation of lysine 27 on histone 3 (H3K27ac)^30,38^. Finally, chromatin accessibility of enhancers ought to correlate with expression of target genes, or upstream partners^42^. Using these molecular features, we explored the hypothesis that enhancers (potentially of spatial genes) drive facial shape variation in adults and/or pathologies in newborns. To link promising cCREs to target genes, we used the LinkPeaks function from the Signac package, which screens for correlations between open chromatin and gene expression (gene–peak pairs referred to as ‘links’), and overlapped the result with published facial enhancer and GWAS datasets, which revealed numerous predicted gene–enhancer interactions (Fig. 3a, Supplementary Note 3, Supplementary Figs. 7 and 8 and Supplementary Table 2).

To validate cCRE importance in vivo, a proof-of-principle experiment was performed (Fig. 3b,h), to target a peak conserved in mouse that is linked to a spatial TF and contains genome-wide significant SNPs identified by previous GWASs^2,7^. This peak resides inside of a long noncoding RNA, roughly 20 kilobases (kb) upstream of its linked gene *PAX1*, which is the gene responsible for otofaciocervical syndrome OTFCS2 (Fig. 3b). *PAX1* is expressed in facial mesenchyme, in sclerotome and in the pharyngeal pouches^43,44^. *Pax1* knockout (KO) mice were reported to develop characteristically long noses (among other symptoms), and OTFCS2 patients with a combination of symptoms and facial dysmorphisms, including a narrow nasal bridge^43,45^. The *PAX1*-cCRE bears enhancer marks (Fig. 3b) and a variant associated with nasal width (Fig. 3c; Methods). *PAX1* was observed in early mesenchymal progenitors and dermal mesenchyme in the general scRNA-seq embedding, and is specifically found in the predermal domain in our Stereo-seq analysis (Fig. 3d), notably in predermal cells adjacent to the forming nasal cartilage. Similarly, *PAX1*-cCRE showed cell-type-specific accessibility (Fig. 3b) in both progenitor cells and differentiated dermis (Fig. 3e). Because lineage tracing studies in mice have suggested that neural crest cells are bipotent across skeletal and dermal tissues, and given that PAX1 is known to engage in transcriptional competition with cartilage master regulator SOX9 (ref. ^46^), *cis*-regulatory influences on the *PAX1* expression domain would be hypothesized to affect development of the frontonasal structures. We identified a 276 bp region within the *PAX1*-cCRE that was 94% conserved between mouse and human and highly conserved across other vertebrates (Fig. 3f). We took advantage of the iGONAD system to use CRISPR editing to delete this conserved region (Fig. 3g). Crossing *Pax1*-cCRE-edited mice consistently produced litters containing mice with smaller bodies, shorter heads and apparent calvarial defects (Fig. 3h). Interestingly, these mice lacked some characteristic features of *Pax1* KO mice, such as scoliosis or a kinked tail^47^. These results provide evidence that the targeted conserved enhancer region is co-activated with the gene *PAX1*, and has importance for mammalian skeletal morphology, but further studies should determine whether this sequence directly interacts with *PAX1*, because LinkPeaks analysis alone cannot effectively distinguish this possibility.

### Associating embryonic facial cell types to facial traits through GWAS signatures

**Fig. 4:**
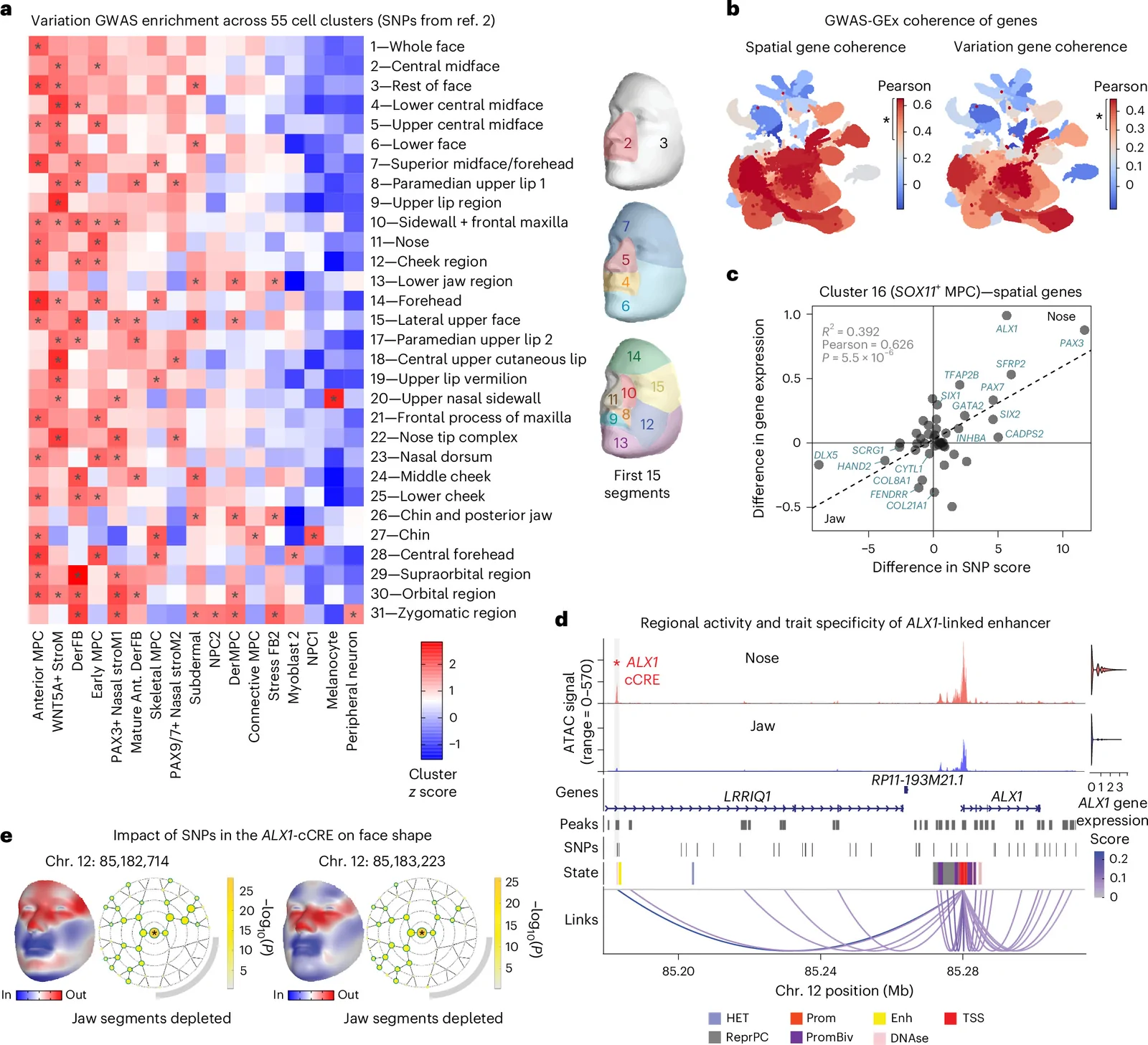
Cellular basis for facial trait specificity. **a**, Left: heatmap showing distance-weighted enrichment *z* scores (Supplementary Methods) for the top 30 traits mentioned in ref. ^2^, across facial cell types (granular clustering). Asterisks indicate significance at *P* < 0.05, based on a one-sided *z* test (uncorrected for multiple comparisons). Clusters not passing *P* < 0.05 for at least one trait from the top 30 were excluded. Trait 16, for which no significant enrichment was observed for any cluster, was also excluded; see full heatmap in Extended Data Fig. 8. Right: illustrated schematic of the top 15 segments from ref. ^2^. **b**, General embedding, where cell types (granular clustering) are color coded by Pearson’s correlation between nose–jaw specificity in SNP significance and nose–jaw differential gene expression (for trait specificity and expression specificity scores, see Methods). Darker red color indicates that the cluster has a greater agreement between localization of gene expression and impact of nearby SNPs, for the spatial genes (left) and genes linked to peaks containing variation SNPs (right). Clusters colored within the indicated starred region are significant (FDR < 0.05). **c**, Scatterplot of trait specificity (*x* axis) and expression specificity (*y* axis) in nose versus jaw for all spatial genes within 500 kb of a lead SNP in ref. ^2^, measured only from cluster 16 (*SOX11*^+^ mesenchyme progenitors). Correlation was assessed using Pearson’s product-moment correlation (two sided; *R*^2^ = 0.392, *r* = 0.626, *P* = 5.5 × 10^−^^6^). **d**, Regional activity of an *ALX1*-linked enhancer. ATAC–seq signal across nose and jaw populations. The cCRE (red asterisk) is highlighted in gray. Gene models for *LRRIQ1*, *RP11-193M21.1* and *ALX1* are shown. Face-related SNPs are indicated in black. Chromatin state annotations (ChromHMM) are shown with predicted enhancers indicated in yellow. Peak-gene links (arcs) connect the cCRE to *ALX1*. Right: violin plot shows *ALX1* expression across the nuclei profiled from different facial regions. **e**, Plot of facial effects for SNPs rs11609649 (chr. 12: 85,182,714) and rs7966105 (chr. 12: 85,183,223) within the *ALX1*-cCRE. Left: normal map—normal distances between the shapes exaggerated in the direction of both alleles for the indicated facial segment. Red represents an outward protrusion, while blue represents an inward depression. Right: radial dendrogram corresponds to facial segments^2^. A dark border indicates genome-wide significance. The asterisk indicates the plotted segment. NPC, neural progenitor cell; HET, heterozygous; TSS, transcriptional start site; Prom, promoter.

**Extended Data Fig. 7:**
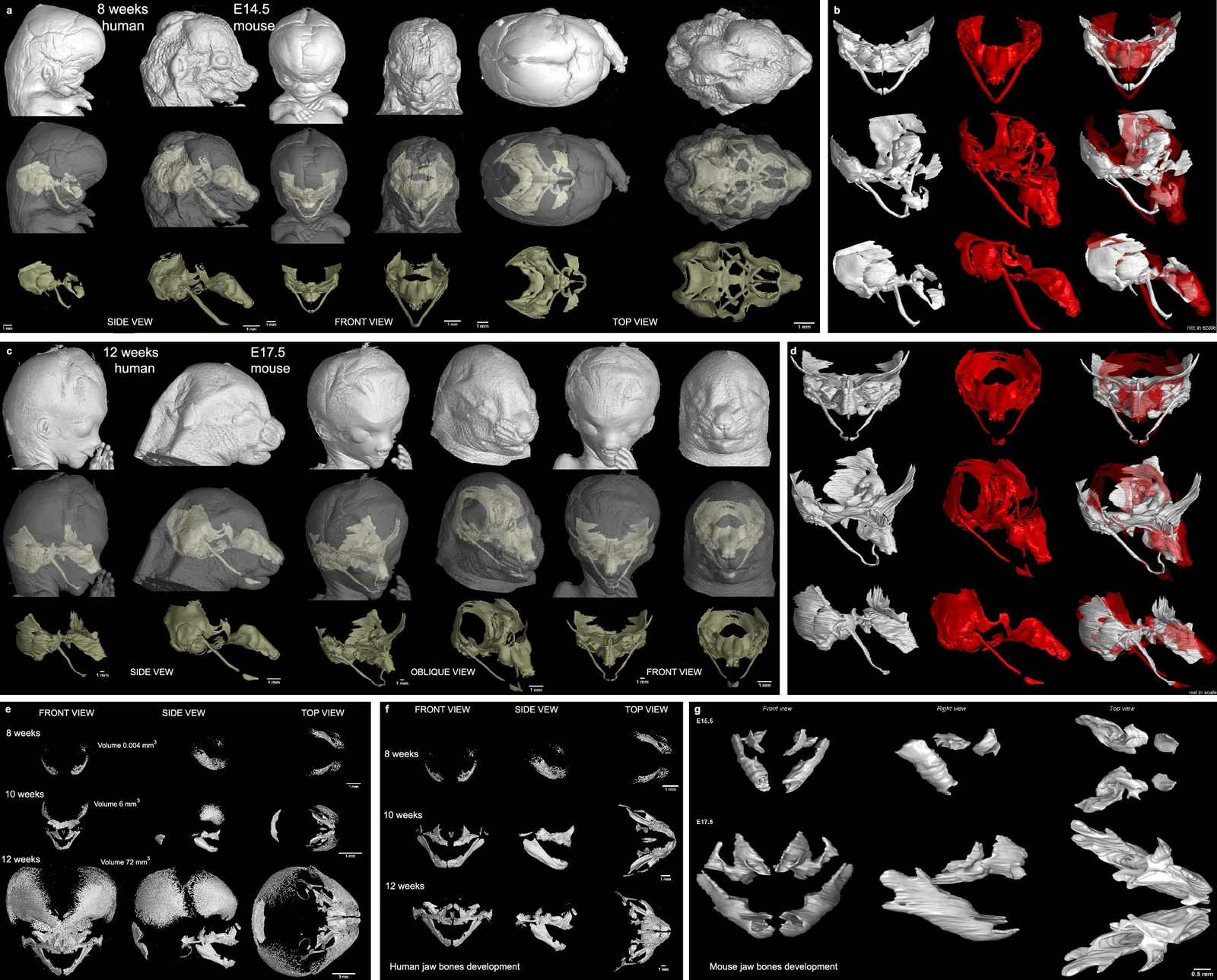
Human and mouse facial skeletogenesis. **a**, Comparison of PCW8 human and E14.5 mouse cartilage growth, shown from different angles (side, front, and top). **b**, Shape overlay of the chondrocrania from **a**. Red = mouse; white = human. **c**, Full panel sequence of the comparison of PCW12 human versus E17.5 mouse cartilage, shown in Fig. 2. **d**, Shape overlay of the chondrocrania from **c**. Red = mouse; white = human. **e**, Full developmental sequence of the bone growth segmented from PCW8, PCW10, and PCW12. **f**, Focus on the development of the human jaw bones at the given stages. **g**, Mouse jaw bone development, shown for stages E15.5 and E17.5.

**Extended Data Fig. 8:**
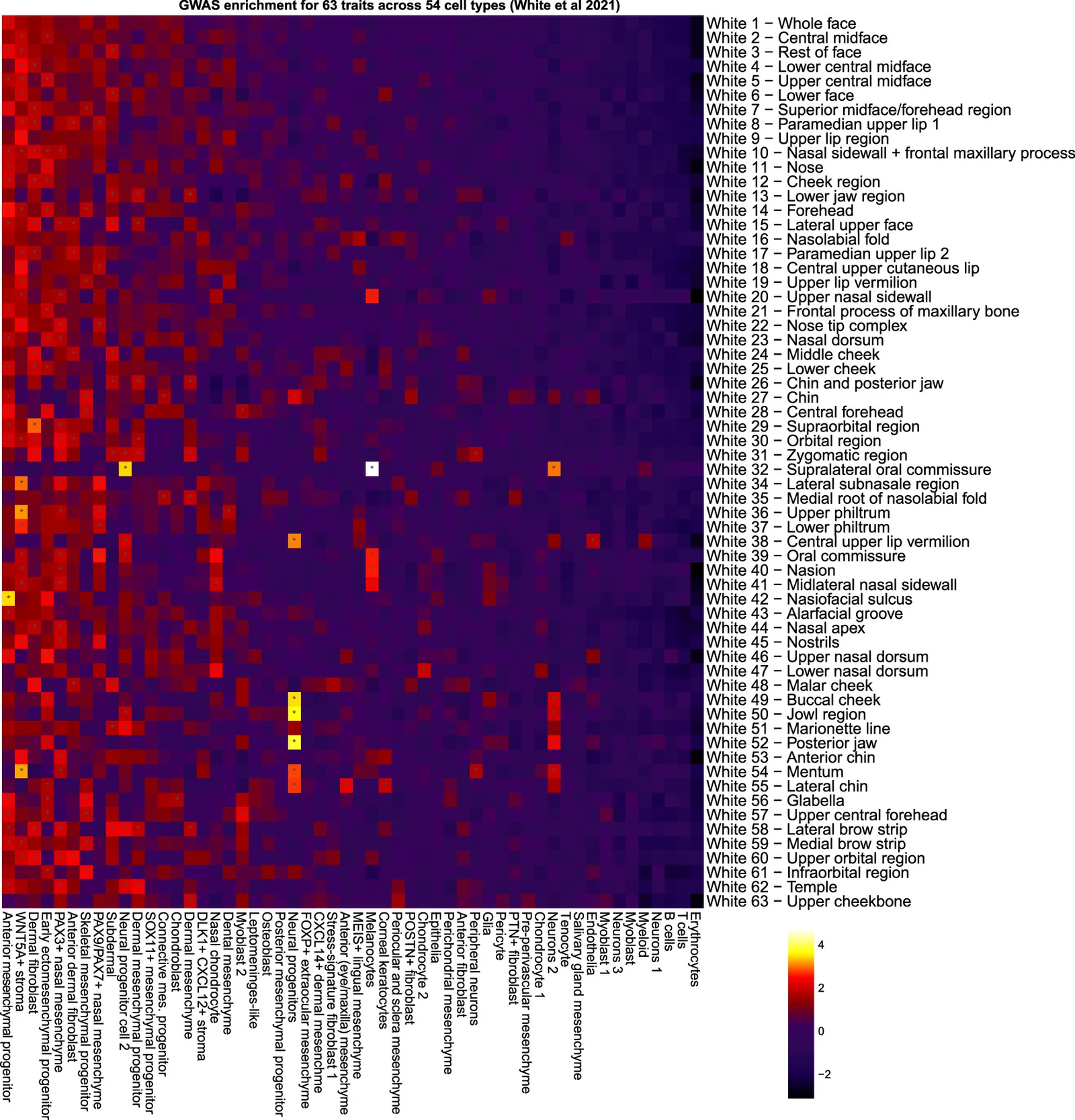
Cellular specificity of enriched facial traits. Heatmap showing distance-weighted enrichment z-scores (Methods) for all traits in ref. ^2^, across all facial cell types (granular clustering). Stars indicate one-sided p-values computed from z-scores < 0.05 (nominal, not corrected for multiple comparisons).

In humans, as well as other vertebrates such as mice, musculoskeletal tissue shapes are generally established early and appear to scale faithfully during development, as indicated by micro-CT scans (Supplementary Fig. 2 and Extended Data Fig. 7), but how cell states influence specific traits remains unclear. To understand the contribution of each cell type to natural variation in facial shaping during embryogenesis, we set out to overlap our datasets with a large facial GWAS^2^, which recently identified thousands of highly significant variants associated with >60 distinct facial traits ordered by size (Fig. 4a and Extended Data Fig. 8; overlaps with several other GWASs can be found in Supplementary Fig. 9). Our aim was to connect SNPs with particular cell populations using the following rationale: (1) most SNPs are located outside of coding regions; thus, a common explanation for their influence is that variants affect enhancer activity. (2) Enhancer-based gene activation occurs within nested TADs, resulting in an inverse relationship between genomic distance and the likelihood of regulatory interactions. To achieve this, a distance-weighted metric of trait enrichment was calculated for each cell type by aggregating gene expression nearby highly significant trait-associated variants (Supplementary Methods). Mesenchymal progenitor cell types were significantly enriched with larger global (top 19) traits compared to all other cell types, consistent with a foundational role in shaping (Fig. 4a). Comparably minor or local traits were enriched in more mature cells (Fig. 4a and Extended Data Fig. 8), suggesting a diminishing influence of cells on facial traits over time. This can be explained in part by how the initial shape of mesenchymal condensations and cartilages provides a template for early endochondral and intramembranous bone deposition (Extended Data Fig. 7), thereby influencing bone morphology even long after the chondrocranium recedes^17^.

We noticed that the highest-ranking mesenchyme population, originating from frontonasal and maxillary prominences, was preferentially enriched in anterior traits such as the upper face (Fig. 4a). Because the pattern of gene expression in the human embryo is expected to influence the shape of the adult face, we tested whether expression localization could explain trait specificity of their genomically proximal variants. A nose–jaw score was calculated by contrasting significance of all SNPs and expression of nearby genes (Methods). Although only weak to no correlation was found when considering all genes, nearly all cell types showed strongly positive correlation between trait expression and trait specificity when considering the 396 ‘spatial’ genes only, as well as when considering genes linked to peaks bearing face-variation SNPs (Fig. 4b). This correlation was well-exemplified by mesenchyme progenitors, notably the PCW6.5-enriched *SOX11*^+^ cell cluster 16 (Extended Data Fig. 2a), as well as some early chondrogenic progenitor clusters (Fig. 4c).

Trait-expression coherence was driven in part by genes encoding spatial TFs with known patterning roles, such as *ALX1*, which is restricted to upper face regions (Fig. 4c). *ALX1*, the gene responsible for autosomal recessive frontonasal dysplasia^48^, marks the upper midface spatial domain (Fig. 2d) and resides near genomic variants with strong upper face specificity, suggesting a functional link. Indeed, we observed an *ALX1*-linked cCRE 80 kbp upstream (residing in an intron of the gene *LRRIQ1*) showing nose-specific accessibility and putative enhancer activity (Fig. 4d), bearing upper face variants (Fig. 4e). Generally, region-specific DARs were found linked to more than 100 of the identified spatial genes (Supplementary Table 2). Gene set enrichment analysis and exploration of the 1000 Genomes project revealed a significant overlap between genes driving variation and congenital disorders, and suggested that variants in enhancers targeting key developmental genes are likely hotspots for generating facial diversity in humans (Supplementary Note 4 and Supplementary Figs. 10–12). Together, these findings reveal a potential basis for variant trait specificity involving localized activation of enhancers in specific populations of progenitor cells.

### Distinct facial traits associated with differentially active enhancers linked to the *MSX1* gene

**Fig. 5:**
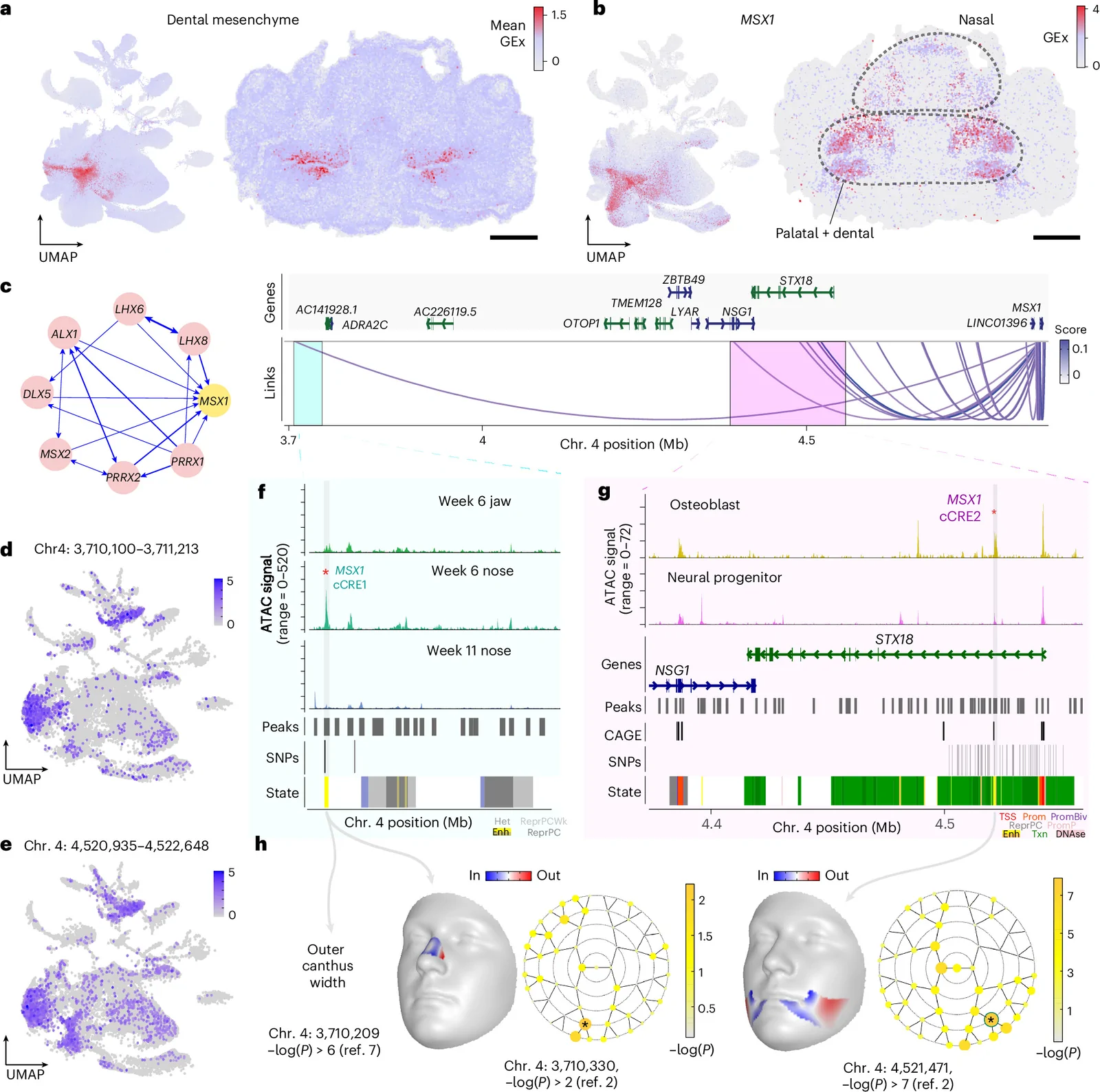
Spatiotemporal and cell-type specificity of multiple MSX1-linked enhancer regions. **a**, Feature plots of the dental mesenchymal cell cluster marker gene signature (Supplementary Table 1) on the general embedding (left) and midrostral section (right). Expression levels are shown using the scale—gray, none; blue, low; red, high. **b**, Plot of *MSX1* gene expression on the general embedding (left) and midrostral section (right). Expression levels are shown using the scale—gray, none; blue, low; red, high. **c**, Predicted eGRN upstream of *MSX1*. Directed edges connect TFs to their targets. Edge color encodes predicted regulation type (blue, activation), and edge width is proportional to the interaction strength (importance × correlation). To the right, long-range candidate enhancer interactions for *MSX1* on chr. 4. Gene models for several genes are shown, along with peak-gene links (arcs) connected to *MSX1*. Two regions are highlighted—one peak around 3.7 Mb (cyan), and five peaks around 4.5 Mb (purple). **d**, Chromatin accessibility of *MSX1*-cCRE1 (chr. 4: 3,710,100–3,711,213) within nuclei in the general embedding. **e**, Chromatin accessibility of *MSX1*-cCRE2 (chr. 4: 4,520,935–4,522,648) within nuclei in the general embedding. **f**, Regional activity of an *MSX1*-linked cCRE1. Normalized ATAC–seq signal across nuclei from PCW6 nose, PCW6 jaw and PCW11 nose samples. The cCRE (red asterisk) is highlighted in gray. Face-related SNPs are indicated in black. Chromatin state annotations (ChromHMM 15-state model) are shown with predicted enhancers indicated in yellow. **g**, Cell-type specificity of *MSX1*-linked cCRE2. Normalized ATAC–seq signal across nuclei from osteoblast and NPC clusters. Gene models for *STX18* and *NSG1* are shown. Below, all ATAC peaks in the region are shown, including the cCRE (red asterisk, highlighted in gray). Bottom: CAGE signal and facial SNPs are shown in black. Chromatin state annotations (ChromHMM 25-state model) are shown with predicted enhancers indicated in yellow. **h**, Predicted impact of rs115587413 (chr. 4: 3,710,209) and rs16835830 (chr. 4: 4,521,471), the SNPs overlapping *MSX1*-cCREs. Left: cCRE1 contains a variant associated with outer canthus width (illustrated) with PV6 (near genome-wide significance, result from ref. ^7^), as well as a variant associated with nasal bridge (PV2, not genome-wide significant, from ref. ^2^). Right: cCRE2 contains an SNP significantly associated with lower jaw shape (from ref. ^2^). Normal distances between the shapes exaggerated in the direction of both alleles for the indicated facial segment. Red represents an outward protrusion, while blue represents an inward depression. Radial dendrogram corresponds to facial segments^2^. A dark border indicates genome-wide significance. Asterisk indicates the plotted segment. Scale bars, 1 mm.

Gene expression patterns are thought to emerge compositely through activity of multiple enhancers with distinct activation profiles, which is supported by the LinkPeaks analysis (Supplementary Fig. 7). We found a striking example of this for *MSX1*, the gene associated with facial anomalies such as oligodontia, cleft palate and Witkop (tooth and nail) syndrome, and is sometimes missing in patients with Wolf–Hirschhorn syndrome (WHS), caused by a partial deletion in the short arm of chr. 4 (refs. ^49,50^). WHS craniofacial severity correlates with deletion size, even in patients where the *MSX1* gene is undisturbed^49^. *MSX1* marks the dental mesenchymal cell cluster (Fig. 5a) and predental spatial domain (Fig. 2d), in line with its role as an important factor during tooth development. However, we also observe *MSX1* expression in the upper midface and nasal region (Fig. 5b). Indeed, *MSX1* expression is highly specific to nasal cartilage compared to other facial cartilages (Extended Data Fig. 5c). To investigate gene regulation of *MSX1*, we consulted our SCENIC results, which suggested that both anterior and posterior facial patterning factors lie upstream in the *MSX1* GRN, possibly representing a mixture of region-specific regulatory interactions (Fig. 5c). Referring to our list of cCREs, we observed multiple *MSX1*-linked cCREs (Fig. 5c–h) accessible in progenitor cells (Fig. 5d,e), showing putative enhancer activity (Fig. 5f,g) and bearing trait-specific variants (Fig. 5h), in agreement with differences in accessibility across regions (Fig. 5f) and cell types (Fig. 5g). This example illustrates how multiple enhancers across different facial regions may compositely establish important gene expression domains during development, having consequences for facial shaping and potentially pathologies such as WHS.

### Cleft-related epithelial gene expression signature reveals pleiotropic enhancer–gene link

**Fig. 6:**
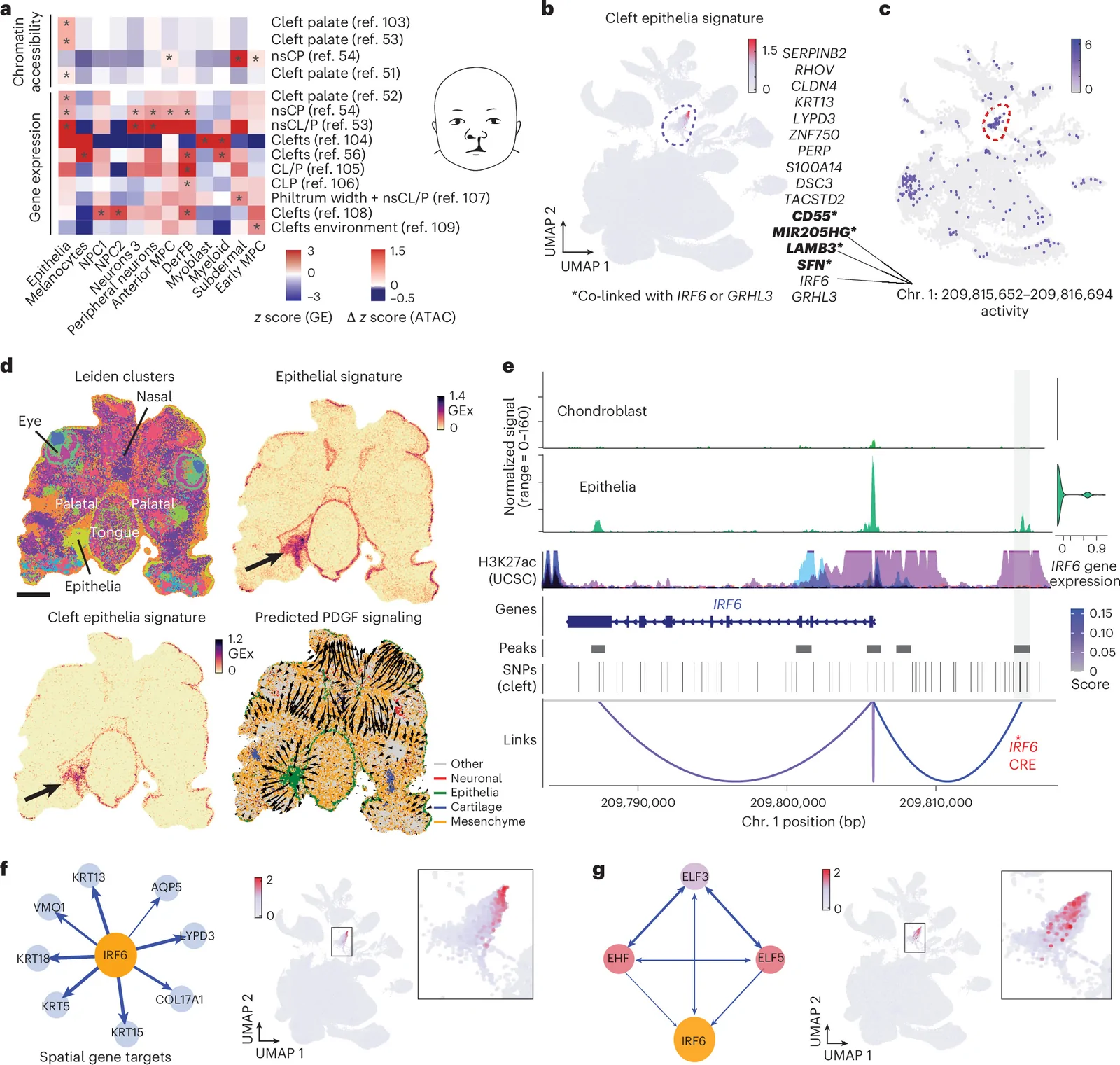
Cleft-associated epithelial-specific gene signature and linked enhancer. **a**, Left: heatmap showing enrichment (*z* scores) for orofacial cleft GWAS SNPs (refs. ^53^^,54^^,71–77^, previously aggregated in ref. ^38^), across facial cell types (granular clustering), for chromatin accessibility (top) and for distance-weighted gene expression (bottom). Asterisks indicate significance at *P* < 0.05 based on *P* values derived from a one-sided *z* test; no multiple-comparison correction applied. See full heatmap in Supplementary Fig. 9. **b**, Left: feature plot of the average expression of genes coexpressed with *IRF6*/*GRHL3* in the general embedding. Right: list of the coexpressed genes. Bolded and asterisk indicate a single peak linked to multiple genes in the signature. **c**, Feature plot of the chromatin accessibility of an IRF6 enhancer within nuclei in the general embedding. **d**, Cleft-associated epithelial gene expression in orofacial tissues in the midcaudal section. Top-left: Leiden clusters are shown as morphological reference. Top-right: feature plot showing average expression of epithelial gene markers (see Supplementary Table 1 for full marker list). Bottom-left: feature plot showing average expression of the cleft-associated epithelial gene signature from **b**. Red, high expression; blue, low; gray, none. Bottom-right: predicted COMMOT signaling activity for the PDGF pathway, color coded by selected cell types. Arrows indicate the directionality and magnitude of signaling scores. Scale bar, 1 mm. **e**, Cell-type specificity of an *IRF6*-linked enhancer on chromosome 1. Normalized ATAC–seq signal across nuclei from epithelial and chondroblast cell clusters. The H3K27ac signal shown is from the UCSC Genome Browser. Bottom: a gene model for *IRF6* is shown. ATAC peaks, including *IRF6*-linked peaks, are shown in gray. Cleft-related SNPs (rs76145088, rs642961, rs77542756, rs11119348) are shown as vertical lines. *IRF6*-cCRE (chr1: 209,815,652–209,816,694) is highlighted in red and marked with the asterisk. Top-right: violin plot of *IRF6* gene expression in epithelia and chondroblasts. **f**, Left: eGRN downstream of *IRF6*. Directed edges connect *IRF6* to its predicted targets (edge width ~ interaction strength). Right: feature plot showing average expression of the same targets in the general embedding. Inset: a magnified view of the epithelial cluster. **g**, Left: eGRN upstream of *IRF6*. Directed edges connect TFs to their predicted targets (edge width ~ interaction strength). Right: feature plot showing average expression of the same genes in the general embedding. Inset: a magnified view of the epithelial cluster. nsCP, non-syndromic cleft palete; nsCL/P, non-syndromic cleft lip or with or without cleft plate.

We wondered if variants underlying craniofacial pathology would also be most strongly enriched in mesenchymal progenitor cell types, but this was not the case when examining GWAS datasets focused on orofacial clefts, as epithelial cells were the most cleft enriched through expression and accessibility across multiple studies^51–54^ (Fig. 6a). We found a population of epithelial cells in our dataset expressing both known cleft palate-related genes *IRF6* (ref. ^55^) and *GRHL3* (ref. ^56^), which act together in a gene circuit that is essential for timely periderm differentiation and clearance during palatogenesis. A gene signature was identified to mark this cleft-related epithelial subpopulation (Fig. 6b), as well as a cCRE linked to multiple genes within this signature—*IRF6*, *MIR205HG* and *LAMB3* (Fig. 6c). The cleft-epithelial signature was found to be expressed in the epithelium overlying the forming palates and in salivary gland epithelia (Fig. 6d), at PCW6 (when periderm formation is beginning). This gene signature partly colocalized with a predicted center for the platelet-derived growth factor (PDGF) signaling pathway based on COMMOT^57^ analysis (Fig. 6d; Methods). PDGF signaling is required for salivary gland morphogenesis^58^ and palatogenesis in mice^59^ and may be broadly deployed across craniofacial epithelial niches. The *IRF6*-cCRE, which overlaps enhancer markings and cleft palate-associated SNPs (Fig. 6e), has previously been described as an *IRF6* enhancer^30^, but to our knowledge has not been linked to *LAMB3* or *MIR205HG*. Accessibility for this pleiotropic cCRE was specific to epithelial cells (Fig. 6e). Finally, SCENIC+ analysis reconstructed a partial *IRF6* GRN, identifying numerous potential targets including *LAMB3*, as well as several spatial genes (including both basal and periderm keratins; these spatial gene targets shown in Fig. 6f) and a restricted set of upstream regulators including genes encoding *ELF5*, *ELF3* and *EHF* (Fig. 6g). This analyzes a glimpse into the potential regulation of cleft-related epithelial markers during human development, as an example that the generated datasets can be used to discover new targets and regulators underlying facial pathologies.

### Peripheral neurons fine-tune the facial skeleton

**Fig. 7:**
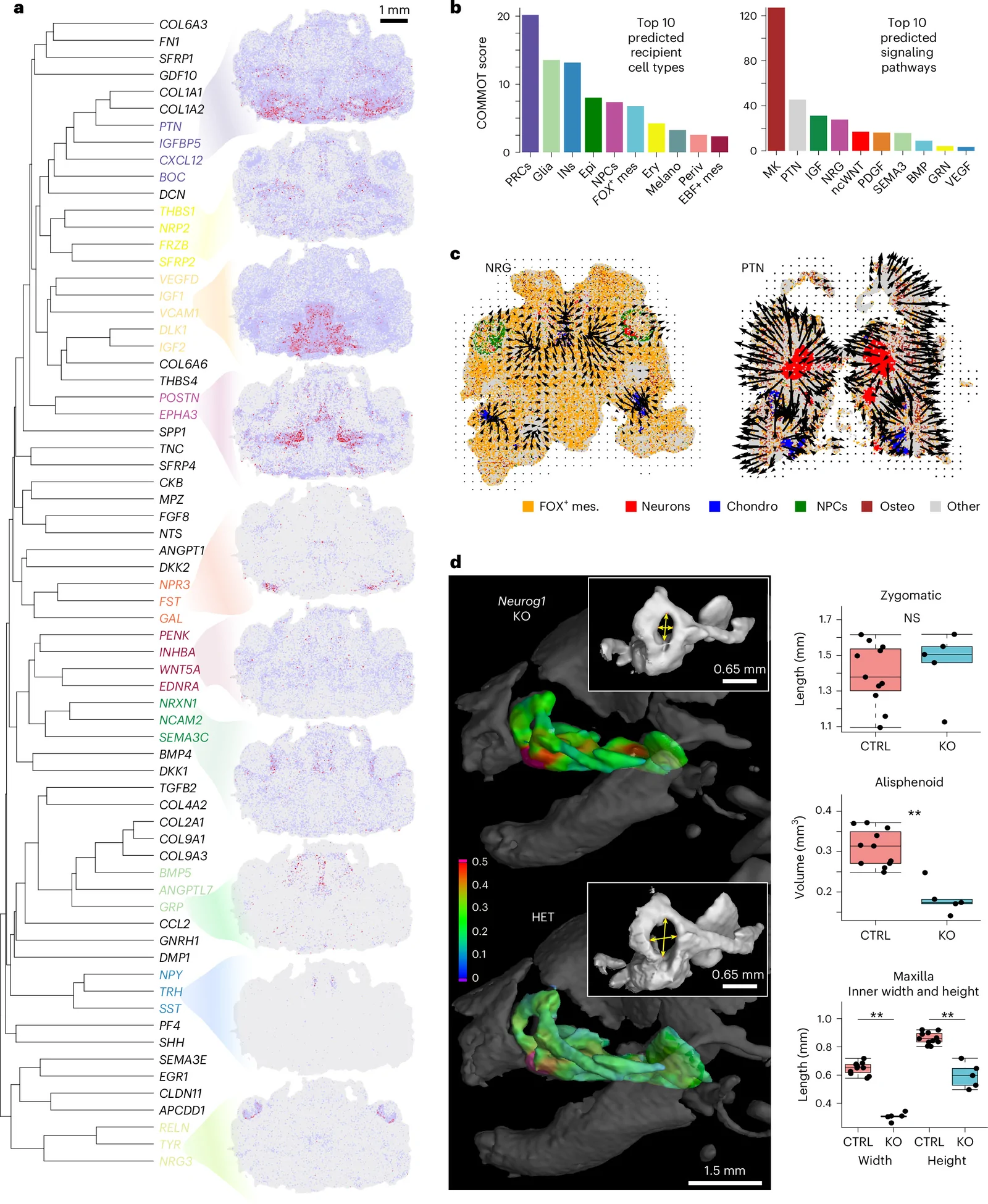
Peripheral neurons may fine-tune craniofacial bone shaping. **a**, Left: hierarchical clustering of spatial signaling genes (secreted molecules or receptors found among the list of spatial genes) based on the distance in a Pearson correlation matrix reflecting coexpression of genes in a downsampled midrostral section (bin = 500). Genes are color coded by module, and highly expressed ECM molecules were excluded from modules to prevent single genes from dominating signatures. Right: combined module gene signatures (averaged) are plotted on the midrostral section (bin = 50) with darker red color indicating higher expression. **b**, Bar plots showing COMMOT scores of the top ten predicted target cell populations (left) and pathways (right) of predicted receptor-ligand signaling activity from neurons. **c**, Predicted COMMOT signaling activity for the NRG (left) and PTN (right) pathways, color coded by selected cell types. Arrows indicate the directionality and magnitude of signaling scores. **d**, Micro-CT analysis of craniofacial skeleton in Neurogenin 1 KO mouse embryos. Left: 3D reconstructions of skulls in *Neurog1*-KO (top) and HET (bottom) control littermates. Maxilla is highlighted and color coded by wall thickness (color scale), and displayed from a slightly rotated angle in the insets. Arrows indicate differences in maxilla shape. Scale bar, 1.5 mm; inset scale bars, 0.65 mm. Right: box plots quantify the length of zygomatic, volume of alisphenoid and maxilla inner width/height (box shows median ± IQR; whiskers show most extreme points within 1.5× IQR). Statistical comparisons between *n* = 5 mutant embryos and *n* = 11 littermate controls were performed using two-sided Wilcoxon rank-sum tests. Significance is indicated as **P = 0.002; not significant (NS), *P* = 0.497. PRCs, photoreceptor cells; INs, inhibitory neurons; Epi, epithelial cells; Ery, erythrocytes; Periv, perivascular cells; Melano, melanocytes; MK, Midkine; PTN, Pleiotrophin; NRG, Neuregulin; BMP, bone morphogenetic protein; VEGF, vascular endothelial growth factor; ECM, extracellular matrix; mes, mesenchymal; EBF, mesenchymal expressing EBF genes; SEMA3, Class III Semaphorin signaling pathway.

Facial domains of localized CRE and/or TF activity are thought to be established in part by secreted signaling molecules. Noticing that receptor-ligand signaling ranked highly among the molecular functions of spatial genes (Fig. 2c), we calculated domains of coexpressed signaling factor modules (Fig. 7a), resulting in similar expression profiles when compared to TF domains (Fig. 2d). Plotting spatial gene cell-type distributions revealed that several of the identified signaling factors were expressed primarily in peripheral neurons (Supplementary Fig. 13). Based on the trait enrichment of peripheral neurons both in variation in zygomatic shape (Fig. 4a) and in cleft palate (Fig. 6a), we grew curious about the role of peripheral neurons in facial growth. COMMOT^57^ was used to investigate the signaling landscape of the developing human face (overview of top pathways and their predicted target genes summarized, Supplementary Figs. 14 and 15). As expected, pathways such as noncanonical Wnt signaling, endothelin, insulin growth factor, PDGF and other established craniofacial-associated pathways showed high levels of activation at PCW6, and COMMOT analysis predicted known target gene associations such as the activation of *DLX5* by endothelin signaling (Supplementary Fig. 15b). We then focused on pathways where neurons are predicted senders (Fig. 7b) and identified cell types that are likely receivers of neuronal secreted signaling (Fig. 7b). This analysis predicted that Neuregulin and Pleiotrophin signaling interactions occur between peripheral neurons and multiple mesenchyme subtypes (Fig. 7c). Incoming Pleiotrophin signaling correlated with activation of numerous spatial genes, as well as important pro-skeletal markers such as *RUNX2* and *ZFHX3* (Supplementary Fig. 15b and Supplementary Table 1). To explore a potential role for peripheral neurons in fine-tuning the facial skeleton, we analyzed craniofacial morphology of mouse embryos lacking cranial sensory ganglia due to genetic KO of Neurogenin 1 (*Neurog1*)^60,61^, and discovered specific facial anomalies (Fig. 7d). Although we did not observe differences in the length of the zygomatic, we found significant and clear differences in the shape of maxilla and alisphenoid (Fig. 7d), suggesting that peripheral neurons influence craniofacial skeletal shaping in mammalian embryos. Further efforts should be aimed at determining whether defects in *Neurog1*-KO bones were caused by altered cell signaling or by other mechanisms^62^.

## Discussion

What cell types contribute most to facial diversity? Although postnatal growth contributes most to changes in a person’s facial appearance throughout life, prenatal development drives variation among faces through a mix of genetic and environmental factors (for example, diet)^32,63^. In agreement with previous embryological work in model organisms, as well as previous epigenomic measurements in humans, our trait enrichment findings point to early ectomesenchyme progenitors as the principal substrates for variants shaping broad facial dimensions, while more localized traits become enriched in progressively differentiated cells, including an unexpected association between peripheral neurons and maxillofacial aspects. As expected, the impact of SNPs strongly correlated with patterns of ‘spatial’ gene expression in mesenchymal cells, suggesting that genetic associations to facial trait variation in adults can be partly explained by effects on patterning of ectomesenchyme cells. When visualizing coexpression among signaling molecules and TFs within this gene list, we observed regional domains that partly correspond to what is known from model organisms (see further discussion in Supplementary Note 5). Whether the identified domains represent gene modules actively competing for cellular real estate at their mutual borders, or are indicative of the outcomes of even earlier patterning decisions, will be interesting to pursue.

Although the human face differs substantially from that of mice and other model organisms^64^, and likely relies on key human-specific regulatory elements^65,66^, human-specific aspects of chondrocranial development remain relatively understudied^67^. In this work, to identify gene targets of human noncoding regulatory elements, we assembled a region-resolved and cell-type-resolved map of 50,000 cCREs whose peak accessibility mirrors gene expression (that is, ‘links’). Building on previous publications^2,30–32,38^, we show that many of these new links overlap genomic regions showing loop-based enhancer logic, as well as numerous eRNAs and validated craniofacial enhancers, collectively indicating that the LinkPeaks approach enriched for bona fide developmental enhancers active in human facial tissues. Overall, these data supported a multitude of evolutionarily conserved and a number of human-specific enhancer interactions. Following this result with massively multiplexed activity assay experiments to define the spatiotemporal windows of enhancer function should help accelerate a resolution to the problem of identifying enhanceropathies, which is a still-growing category of diseases^31,39,68–70^.

## Methods

### Ethical approval

The Swedish Ethical Research Authority and the National Board of Health and Welfare approved all procedures for acquiring human prenatal tissue for research (ethical references 2018/769-31, 2019-04595, 2020-03045 and 2024/00280-02). The use of fixed embryos for micro-CT analysis was approved by the Ethics Board of Masaryk University (ethical reference is 79/2022). All animal experiments were approved by the Swedish Animal Welfare Body, Jordbruksverket and the regional animal ethics committees, Stockholms djurförsöksetiska nämnd and Göteborgs djurförsöksetiska nämnd (permits N15907-2019, 18314-2021 and 004225)

### Collection of human embryonic tissue

Human prenatal tissue was obtained from elective terminations of pregnancy (induced abortions) with informed oral and written consent from the patient, and pseudonymized. The material was donated for general nonprofit research purposes, especially with a focus on embryonic tissue development. Sample staging involved clinical ultrasound, using crown-rump length and anatomical landmarks. The chondrocranial region and surrounding tissue were separated and placed in ice-cold PBS before tissue dissociation. This study analyzed 20 embryos using transcriptomics-based methods (between 6 and 11.5 weeks of gestation). For morphological analysis by microcomputed tomography, human embryos from the repository of the Department of Anatomy, Faculty of Medicine, Masaryk University, Brno, were fixed in formaldehyde solution. They were sourced from the Maternity Hospital of the University Hospital Brno on Obilní trh, collected in the second half of the 20th century. Five embryos were used for micro-CT in the study (at 8–12 weeks of gestation).

### Phosphotungstic acid contrasting

To enhance soft tissue contrast for micro-CT scanning, we used a staining protocol adapted from Brian Metscher’s laboratory (University of Vienna). This involves fixing embryos in a formaldehyde solution, followed by a dehydration process using varying ethanol concentrations. For enhanced scanning results, we used phosphotungstic acid for contrasting the samples as described^21^. Post-treatment, the embryos were rehydrated and embedded in agarose gel for stability during X-ray CT scanning.

### Micro-CT scanning of human embryos

The micro-CT analysis of human embryos was executed using a GE phoenix v|tome|x L 240 system, equipped with a nanofocus X-ray tube and a high-contrast flat panel detector as described^21^. The acceleration voltage of X-ray tube was 80 kV with 200 µA current. Exposure time was 500 ms and three projections were acquired and averaged in each position (2,100–2,300 projections depending on the sample width) over 360 °C. The voxel size of the generated volumes varied depending on the embryo size in the range between 11 and 20 µm, and tomographic reconstructions were performed with specialized GE phoenix datos|x software. For the cartilage segmentation, Avizo software was used, and the data were transformed into a polygonal mesh. Surface smoothing of this mesh was done using VGSTUDIO MAX 2.2 software.

### Animal experimentation

Animals from nongenetically modified mouse lines CD-1 and C57BL/6 were purchased from Charles River Laboratories and Janvier. Neurogenin 1 KO (‘*Neurog1Tm2and*’) mice were purchased from Jax Lab (RRID: IMSR_JAX:017306). Animals were kept in an SPF animal facility with standardized conditions (24 °C, 12-h light/12-h dark cycle, normal humidity, food and water ad libitum). After producing the *Pax1*-cCRE strain using iGONAD, heterozygote mice were crossed to generate experimental and control animals distinguished by size.

### Micro-CT analysis of mouse bones

For scanning of mouse bones during functional experiments for the role of peripheral nerves (*n* = 5 E18.5 *Neurog1*-KO mouse embryos, *n* = 11 littermate heterozygote/wild-type mouse embryos, from *n* = 3 litters) and the role of the predicted Pax1 enhancer (*n* = 6 ‘small’ mice from the *Pax1*-cCRE line and *n* = 5 littermates, from *n* = 2 litters at 2 months of age), mice were killed, fixed in 4% paraformaldehyde for 24–48 h at 4 °C, stored in 70% ethanol and subsequently analyzed by micro-CT scans at the Karolinska Experimental Research and Imaging Center within the Department of Comparative Medicine at Karolinska University Hospital. These CT scans were performed using the Mediso nanoScan PET/CT system, operated through Nucline software. The acquisition parameters were as follows: 720 projections per rotation, helical scanning mode with a pitch of 1, Ultra Zoom setting, exposure time of 170 ms, binning ratio of 1:4 and an X-ray tube voltage of 35 kVp. Image reconstruction was conducted using Fourier back projection within the same software environment. The resulting volumetric dataset consists of isotropic voxels with an edge length of 49 µm. Downstream image analysis was performed at the Central European Institute for Technology using Avizo software and VGSTUDIO MAX 2.2 software as above.

### Tissue dissociation and single-cell suspension preparation

Tissue digestion buffer was prepared in advance of the dissection—1 ml of the 20 mg ml^−1^ Collagenase P (Sigma-Aldrich), 1 ml of 25 U ml^−1^ dispase (Gibco/Invitrogen), 1 ml of 10x TrypLE Select and 7 ml of Hank’s buffered salt solution (HBSS; HyClone, with Ca^2^^+^ and Mg^2+^, without phenol red). Whole craniofacial complex samples, and external ear samples, were received from the Human Developmental Tissue Biobank. Then, dissection of different regions was performed under a stereoscope with darkfield illumination to enrich samples for cells originating from a specific facial region (see Supplementary Methods for regional dissection details). Each dissected tissue (lower jaw region, nasal region, ears region, eyes region and forehead region) was chopped into 1 mm pieces with a scalpel and placed into 15 ml Falcon tubes containing 2.5 ml tissue digestion buffer. Then, tissue was gently triturated using a transfer pipette and briefly vortexed. The samples were placed in a 150 rpm shaking incubator at 37 °C and digested for four periods of 5 min each, agitating each sample in between each incubation period. Agitation consisted of tapping the tube, vortexing 5 s, triturating 10x with a transfer pipette, and triturating 10x with a 1 ml pipette. After a final agitation following digestion, the remaining tissue chunks were allowed to gravitate to the bottom. Supernatant was collected after 30 s. The supernatant was transferred to a new 15 ml Falcon tube and 2% FBS in HBSS (without Ca^2+^, without Mg^2+^, without phenol red) was added on ice to each tube until they were filled up to 10 ml, and from then on samples were kept on ice. Samples were centrifuged at 500*g* for 15 min at 4 °C, and each pellet was resuspended in 2 ml of 2% FBS/HBSS and subsequently passed through a 40-µm filter. Cell suspensions were placed into 2 ml Eppendorf tubes and centrifuged at 500*g* for 5 min at 4 °C on a tabletop centrifuge. Supernatant was removed and cell pellets were resuspended in 100 ml of PBS/0.05% BSA. For samples that would be used to make gene expression libraries, 400 ml cold methanol was added dropwise to the cell suspension, and the tubes were equilibrated to a −80 °C freezer using a Mr. Frosty freezing container. For samples that would be used to make 10x multi-omics (RNA + ATAC) libraries, pellets were instead suspended in 20% FBS (HyClone), 10% dimethyl sulfoxide (Sigma-Aldrich), in RPMI media. On the day of library preparation, the cells were brought to +4 °C and centrifuged at 1,000 RCF for 10 min at 4 °C. Supernatant was removed and 500 μl of rehydration buffer (1x DPBS (Gibco, 14190144) containing 1.0% BSA (Sigma-Aldrich, B4287) and 0.5 U μl^−1^ RNAse out (Thermo Fisher Scientific, 10777019)) was added to cell pellet, resuspended and centrifuged at 1,000 RCF for 10 min at 4 °C. The cell suspension was sorted and debris was removed by FACS using a BD FACS Aria Fusion instrument equipped with 100 μm nozzle (BD Biosciences). FACS-sorted cells were pelleted again and resuspended in rehydration buffer, and concentrated until the sample concentration was roughly 10,000 cells in 50 μl. Nuclear isolation for multi-omics experiments was performed in accordance with the 10x Genomics Chromium Single Cell Multiome ATAC + gene expression kit.

### RNA-seq library preparation and sequencing

Library preparation was done in accordance with 10x Genomics Chromium Single Cell 3′ protocol for Reagent Kits (v3.1), and the 10x Genomics Chromium Single Cell Multiome ATAC + gene expression kit. For human fetal cells, we aimed to recover 10,000 cells or nuclei per chip with the Illumina NovaSeq 6000 Sequencing System (NovaSeq 6000 S1 Reagent Kit or NovaSeq 6000 S2 Reagent Kit was used). The read setup was the standard recommended from 10x Genomics—read 1 = 28 cycles (cell barcode and UMI), i7 index = 8 cycles (sample index) and read 2 = 91 cycles.

### Processing of scRNA-seq

A total of 30 scRNA-seq and 12 snRNA-seq datasets from human embryo samples between 6 and 11 weeks were analyzed by the Scanpy package (v1.9.3). We kept only cells with a number of detected genes of >200 and cells with mitochondrial gene expression percentages <4.5. Genes expressed in <3 cells were removed. To detect doublets, Scrublet was performed on each sample by setting the parameter ‘threshold = 0.15’ (ref. ^78^). Sex-related genes were removed from the gene list to avoid batch effects. We merged the count matrices from all the samples using an inner join of genes across sample batches resulting in a merged count matrix containing 295,141 cells and 21,237 genes. Next, the gene expression matrix was normalized and log transformed. Highly variable genes were calculated and the PCA was performed. The cell cycle was regressed out using Scanpy’s ‘regress_out’ function. For nose and jaw embeddings, batch effect correction was conducted on the principal components (PCs) with Harmony algorithm^79^. The neighborhood graph computed by the pp.neighbors function was used for clustering performed by the Leiden algorithm^80^.

### Developmental trajectory inference from scRNA-seq data

We used the Python command-line velocity tool to generate a loom file for each sample based on the BAM files of the samples^81,82^ to determine amounts of spliced versus unspliced transcripts. The spliced counts, unspliced counts and highly variable genes were used to calculate velocity values. To aid trajectory inference during the analysis of the count matrix, we used the scFates Python packages^83^. PCA was performed on the count matrix subset of the 1,500 highly variable genes. Palantir was used to obtain multiscale diffusion space and then embedding from the multiscale diffusion space was generated^84^. The trajectory inference was performed in two stages. For nose and jaw harmonized embeddings, a 200-node tree was fitted, using SimplePPT approach on the UMAP space using tl.tree function (scFates, method = ‘ppt’ sigma = 0.5, lambda = 100, metric = ‘euclidean’). For individual samples, a 3,000-node tree was fitted, using SimplePPT approach on the diffusion space using tl.tree function (scFates, method = ‘ppt’ sigma = 0.0005, lambda = 200, metric = ‘euclidean’). The tree is composed of a soft assignment matrix R, where each node of the tree contains a value for all cells; each cell has an assignment strength to a node between 0 and 1, with 1 being the closest to the node. Pseudotime value was then generated as a distance on the tree from the selected roots and projected to cells using the R assignment matrix. For analysis using CytoTRACE, the subset count matrix was used as input for the CytoTRACE pipeline using default parameters^85^.

### Visium and Stereo-seq sample preparation and data analysis

Human tissue samples were snap frozen in OCT using liquid nitrogen and stored at −80 °C. Frozen tissue blocks were sectioned on a Leica NX70 cryostat and placed on a 10x Visium capture slide. RNA quality of all samples was determined to be RIN > 9. Tissue optimization slides were used to determine the timing of tissue permeabilization. Library preparation was performed according to the user manuals for Stereo-seq (https://en.stomics.tech/) and Visium (https://www.10xgenomics.com/). The detailed notebooks used to analyze the data are available at https://github.com/TBouderlique/human_face_development. Briefly, Visium reads were processed with Space Ranger (10x Genomics). To account for dropouts, we denoised the datasets. The denoised and raw counts were then processed with the Scanpy pipeline^86^. The denoised count tables were used only to compute the neighborhood graph and cluster the Visium spots. The GWAS enrichment scores were calculated on the normalized raw counts. We calculated the cell-type scores with Tangram^87^, using a reference scRNA-seq corresponding to the section, coming from the same sample when possible. For Stereo-seq analysis, the read mapping, counting and cell segmentation were performed with SAW pipeline^88^. After that the conversion to h5ad was performed with Stereopy (v0.12.1)^89^. Annotation of Stereo-seq data was done using label transfer from the scRNA-seq data with CellTypist (v1.6.3)^90^. Inference of cell signaling communication in all spatial datasets was performed using COMMOT^57^. To analyze target genes, the top global gene activation from pathways was retrieved from the COMMOT results. COMMOT outputs the significantly differentially expressed genes in response to a pathway using associationTest() from tradeSeq^91^. It also fits a smooth curve to each pathway, and with this, we filtered for positive associations with gene expression to retrieve gene activation. For each sample, the top 50 gene–pathway activations were selected. Genes that contribute to the pathway activation score (for example, receptors) were removed. Among these genes, across all samples, the greatest strength of association score for each pathway–gene pair was selected.

### Spatial codes analysis

For cell and bin embedding creation, log1p transformation followed by PCA was performed with Scanpy^86^. In the case of cells’ embedding from Stereo-seq data, the first PC was excluded from further analysis as it captures only differences in cells’ sequencing depth. *K*-nearest neighbor graphs were built based on the embeddings and were further used for clustering with the Leiden algorithm^80^. Spatially restricted and cluster nonrestricted cells were detected as a trade-off between Moran’s I of spatial graph and Moran’s I of gene expression graph. More precisely, Squidpy (v1.2.2)^92^ was used to calculate Moran’s I^35^ on both spatial and gene expression graphs. Per tissue section, the top 100 genes with the highest difference between spatial Moran’s I and squared root gene expression Moran’s I were selected as potential genes of interest. This resulted in 396 unique spatial genes saved from at least one section (Supplementary Table 3 shows averaged scores) and 134 unique genes saved across both embryos. For downstream analysis, we proceeded with the 396 genes. These genes were curated by known molecular functions into lists such as transcriptional regulators or secreted signaling or extracellular matrix factors. To assess coexpression profiles, Stereo-seq sections were downsampled into roughly 400 bins (bin size = 500) and Pearson’s correlation was calculated across gene pairs based on expression per bin. Hierarchical agglomerative clustering with average linkage (UPGMA) on a correlation-distance matrix followed by module assignment through dendrogram cutting using the ‘cutree’ package (*k* = 25) was used to define gene modules, with some adjacent genes added or excluded based on expression profile similarity. A consensus tree was constructed using all spatial genes and all Stereo-seq sections, but spatial TF and signaling modules were analyzed using the midrostral section as it shows the most regions of interest and above-median agreement with the consensus tree (Supplementary Table 3).

### Preprocessing and normalization of joint snRNA/ATAC–seq data

Cell Ranger ARC with default parameters was used on all samples to obtain count matrices for both modalities by aligning reads to the human genome and transcriptome (hg38). Resulting samples included count matrices from 6.5 weeks (first embryo face sectioned into eyes, nose, jaw regions; second one with anterior and posterior regions) and 10 and 11 weeks (divided into eyes, nose, jaw regions and a sample with remaining tissues). The resulting gene expression, ATAC–seq count matrices and fragment files were preprocessed using Seurat (v5.0.0) and Signac (v1.14.0) packages on R (v4.4.2)^93–95^.

To merge multiple single-cell ATAC–seq datasets, we first created a unified set of peaks using the ‘reduce()’ function from output bed files. Next, we quantified peaks in each dataset using features from a unified set of peaks and fragment files and created a feature matrix with ‘FeatureMatrix()’. Then we created objects for each dataset using ‘CreateChromatinAssay()’ and ‘CreateSeuratObject()’. The resulting 12 objects were merged using the merge() function. For quality-control metrics, we computed ‘NucleosomeSignal()’ and transcription start site (TSS) enrichment score ‘TSSEnrichment()’ and used ‘subset()’ to filter out the low-quality peaks using the following thresholds: TSS.enrichment > 4 and <10, nucleosome_signal < 2 and for the total number of ATAC–seq fragment counts per cell nCount_ATAC < 250,000 and nCount_ATAC > 1,300. We removed peaks on nonstandard chromosomes and in genomic blacklist regions with ‘keepStandardChromosomes()’ and ‘subsetByOverlaps()’. GC content for each peak was computed using ‘RegionStats()’ (EnsDb.Hsapiens.v86 (v2.99.0), GenomeInfoDb (v1.42.0))^96^. We further processed DNA accessibility data by performing latent semantic indexing by finding top features for peak assay ‘FindTopFeatures()’, performing term frequency inverse document frequency normalization ‘RunTFIDF()’ and performing partial singular value decomposition with RunSVD().

Gene expression count matrices were merged using the ‘merge()’ function following ‘JoinLayers()’. The result was added to the existing ATAC–seq object using ‘CreateAssayObject()’. RNA assay was filtered using subset() function for percentage of mitochondrial genes percent.mt < 10; for the total number of molecules detected within a cell nCount_RNA < 20,000 and nCount_RNA > 2,000; for the number of genes detected in each cell nFeature_RNA < 1,000 and nFeature_RNA < 5,000. Then, gene expression data for each dataset were normalized using ‘NormalizeData()’ for the total number of counts per cell, multiplied by a scaling factor 10,000 and natural-log transformed. A total of 2,000 variable features (genes) were found using ‘FindVariableFeatures()’ with the ‘vst’ selection method. RNA assay was scaled and centered using ‘ScaleData()’ for all genes. Furthermore, the gene expression assay was normalized with ‘SCTransform()’ and we reduced dimensionality with ‘RunPCA()’.

### Downstream multi-omic analysis and visualizations

To find a set of peaks that may regulate gene expression, we computed the corrected correlation between gene expression and accessibility of nearby peaks using the ‘LinkPeaks()’ function^42^. We used 3 Mbp distance from the genes as threshold. Results for particular genes and peaks were visualized using ‘CoveragePlot()’. SNPs associated with facial features or craniofacial diseases were overlapped using peak coordinates. GWAS SNPs were reused from published studies^2–7,38,97^. Minimum *P* values for each SNP/segment pair from ref. ^2^ were collected by comparing UK and US cohorts. A total of 25-state model coordinates based on ChIP–seq from Carnegie stage 17 (ref. ^30^; ~6 weeks) and H3K27ac ChIP–seq data^30,38^ from weeks 4, 5, 6, 7 and 8 were downloaded from GEO. CAGE^+^ regions were re-analyzed^32^ as described above. Vista enhancers were downloaded on 19 September 2025 from the VISTA Enhancer browser tool^98,99^. LiftOver (rtracklayer) was used to ensure coordinates across all datasets matched the hg38 reference genome. Overlap between peaks or linked peaks with other datasets was identified using subsetByOverlaps() and findOverlaps() functions from the GenomicRanges (v1.58.0 and v1.60.0) and IRanges (v2.40.0) packages in R (v2025.09.0 + 387)^96^. A total of 572 links had SNP overlap, and together, links overlapped 874 SNPs in total. The 12,699 CAGE regions overlapped with 8,762 links. Thus, 6,874 links were found to contain an H3K27ac^+^ region. Cell-type annotations, both the general coarse-grained and more fine-grained annotations (56 cell types), were made on the merged dataset based on gene expression for all experiments containing both snRNA-seq and scRNA-seq data (filtered altogether; ‘Processing of scRNA-seq’). For the ATAC–seq assay, differentially accessible peaks between cell types, sectioned regions and collected time points were found using ‘FindAllMarkers()’ and ‘FindMarkers(min.pct = 0.1)’ functions using the Wilcoxon test. The resulting DAR activity for various cell types and spatial–temporal groups of cells was visualized using pheatmap (v1.0.12). For this, the average openness of cells was calculated using ‘AverageExpression()’, and resulting values were *z* scaled before plotting. Cell types were hierarchically clustered based on correlation distances and the Ward.D2 linkage method, resulting in an order applied to both the columns and rows of the heatmap. We added motif information to the Seurat object using the ‘AddMotifs()’ function and a list of motif position frequency matrices from the JASPAR database (JASPAR2020, v0.99.10)^100^. To find enriched motifs in a given set of cell-type-specific DARs compared to a GC-content-matched set of peaks, we applied the ‘FindMotifs()’ function. To examine disease association of peaks, we took the various sets of peaks that contained trait-associated SNPs and calculated the average peak accessibility for this set on a single-cell level, subtracted by the aggregated accessibility of control feature sets using AddModuleScore() from Seurat. All the tables and visualizations were handled using dplyr(v1.1.4), tydir(v1.3.1) and ggplot2(v3.5.1) libraries in R.

### SCENIC+

To build eGRNs using SCENIC+ (refs. ^101,102^), we first created metacells using SEACells (v0.3.3) on Python (v3.11)^103^. We used latent semantic indexing dimensionality reduction of the scATAC-seq assay to create metacells, aiming for 75 cells per metacell. Fifteen eigenvalues were used for initializing the metacells. Created ATAC–seq metacells were then used to summarize gene expression values for cells in each metacell and to assign the probable granular cell type. The resulting metacell ATAC–seq object was converted into cisTopic objects with create_cistopic_object() and then used for downstream analysis with pycistopic (v2.0a0) for enhancer candidate identification and topic modeling^104^. Next, topics were binarized and annotated by cell type, and DARs and genes were found across cell types (find_diff_features()). For the RNA-seq part of the metacells, we preprocessed the data using Scanpy (v1.8.2)^86^. Count matrix was normalized (sc.pp.normalize_total()), log tranfromed (sc.pp.log1p()) and scaled (sc.pp.scale()). Because we had human data, we used precomputed cisTarget ranking, score databases and SCENIC+ motif collection, cisTopic object and preprocessed GE matrix to run the SCENIC+ pipeline with default parameters with the 2 Mbp search space upstream and downstream of TSS of a gene to create eGRNs.

### Visualization of SNP impact

We investigated the facial effect of SNPs using summary statistics from a GWAS meta-analysis focused on 63 hierarchical facial regions in 4,680 participants from the United States and 3,566 from the United Kingdom^2^. For each SNP, we obtained the multivariate effect coefficients corresponding to the facial region and cohort with the strongest association (that is, lowest *P* value). Briefly, these effect sizes were derived through canonical correlation analysis, applied to PCs that capture facial shape variation. The PCs were adjusted for covariates including sex, age, age squared, height, weight, facial size, the first four axes of genomic ancestry and the imaging system used^2^. The multivariate effect coefficients yielded by canonical correlation analysis can be interpreted as the axis of shape variation most strongly correlated with the SNP. To visualize the phenotypic effects, we computed normal distances between facial shapes positioned at ±3 s.d. along the identified axis, effectively exaggerating the effect of both alleles. These distances were rendered as heatmaps, with blue and red indicating and outward displacements, respectively. To better understand the span of an SNP’s effect on the face, its *P* values across all 63 facial regions were plotted on a polar dendrogram structured according to facial regions defined in ref. ^2^—the central node represents the full face, while the surrounding quadrants, arranged clockwise from the top-right, correspond to the upper face, lower face, nose and upper lip. The MeshMonk (v0.0.6) spatially dense facial mapping software is free to use for academic purposes (https://github.com/TheWebMonks/meshmonk). MATLAB 2017b implementations of the hierarchical spectral clustering are available from an earlier publication^105^.

### CRISPR–Cas9

Target sites for gRNAs of the human homolog sequence were identified using RGEN Tools, Cas-Designer 2023.05.05 (BAE Labs, Seoul National University College of Medicine). All genome editing reagents were purchased from Integrated DNA Technologies. CRISPR RNAs (crRNAs) for candidate sites and tracrRNA were complexed to form a crRNA–tracrRNA complex (called ‘gRNA’) and incubated with Cas9 for RNP formation. The design of long ssDNA was based on the details mentioned in ref. ^106^, taking into account (1) the polarity of the repair template, (2) asymmetry of the homology arms and (3) the removal of putative internal homology between the Cas9 cleavage and insertion sites by introducing (silent) mutations in the repair template. The replacement for the human homolog was designed by choosing a non-native random nucleotide sequence of 274 bp length. Long ssDNA was purchased from Integrated DNA Technologies. Human homologous sequence in mouse chr2 was replaced by randomized nucleotide sequence bearing no similarities to original and none of the found TF binding sites. Guide RNAs were chosen 80 bp upstream and 67 bp downstream of the region to be replaced. Homologous arms were chosen 50 bp above and below each guide’s PAM. See Supplementary Table 6 for sequences used. Details of the iGONAD and downstream genotyping can be found in the Supplementary Methods.

### Reporting summary

Further information on research design is available in the Nature Portfolio Reporting Summary linked to this article.

## Online content

Any methods, additional references, Nature Portfolio reporting summaries, source data, extended data, supplementary information, acknowledgements, peer review information; details of author contributions and competing interests; and statements of data and code availability are available at https://doi.org/10.1038/s41588-026-02748-y.

## Supplementary information

Supplementary Information Supplementary Notes 1–5, Supplementary Methods and Supplementary Figs. 1–15.

Reporting Summary

Peer Review file

Supplementary Tables 1–7 Supplementary Table 1: Cell-type markers and gene signatures. Left, each Leiden cluster (fine-grained annotation) is listed according to both shorthand and extended descriptive format. Right, gene signatures that can be used to identify the cluster in the CELLxGENE browser are listed. Supplementary Table 2: A facial link dictionary. Each linked peak and linked gene are shown, as well as the status of the peak, as far as overlapping SNPs, chromatin states, enhancers, DAR status, human-specific enhancers, Hi–C category and evolutionary conservation. Supplementary Table 3: Spatial genes. From left to right, list of genes that were top ranked in at least one section, and their corresponding score (Methods); the list of unique genes top ranked within each of two biological replicates; the list of genes that are differentially expressed across chondrocytes from different regions; information about the consensus tree, in terms of alignment of individual samples with the consensus (top) and consensus module assignment of each spatial gene (bottom). Supplementary Table 4: Enrichr-based gene set enrichment analysis of the spatial gene list. Supplementary Table 5: Gene sets underlying Supplementary Fig. 10. Supplementary Table 6: Guide RNAs, donor sequence and primers for the Pax1-cCRE experiment. Supplementary Table 7: CAGE peaks used in the study.

## Data Availability

Please explore the dataset on CELLxGENE at https://adameykolab.hifo.meduniwien.ac.at/cellxgene_public/. CAGE data have been made available in the Supplementary Table 7. The raw CAGE data have also been analyzed in a different context by our team as part of a different published paper^32^ (accessible at https://zenodo.org/records/10363659 (ref. ^107^)). Micro-CT scans are available at Zenodo via https://doi.org/10.5281/zenodo.17347142 (ref. ^108^). Count matrix data have been submitted to Dryad, and is accessible under the following https://doi.org/10.5061/dryad.7m0cfxq8t. All raw FASTQ files have been deposited to the European Genome-Phenome Archive and are available upon request from the accession EGAS50000002001.

## Code availability

Original code has been archived at Zenodo via https://doi.org/10.5281/zenodo.21825224 (ref. ^109^) and is available on GitHub at https://github.com/TBouderlique/human_face_development.
