# Supplementary material for: Atlas of cell types and regulatory elements underlying human facial diversity
Source: Nat Genet. 2026 Sep 24;58(10):2645–59. doi: 10.1038/s41588-026-02748-y (PMC13649600; doi:10.1038/s41588-026-02748-y)
Supplement: Supplementary file 1 [file 41588_2026_2748_MOESM1_ESM.pdf]

nature genetics

Supplementary information https://doi.org/10.1038/s41588-026-02748-y

Atlas of cell types and regulatory elements underlying human facial diversity

In the format provided by the authors and unedited

Supplementary Note 1: Exploration of the underlying transcriptional trajectories and mesenchymal cell fate decision hierarchy

To explore morphological correlates of chondrogenic and myogenic differentiation, we reconstructed anatomical structures emerging as cells traverse from early to late gene expression states (**Supp. Fig. 2a-h**). Cartilage and muscle form in concert and are readily distinguishable from CT scans already at PCW8 (**Supp. Fig. 2a**)[1]. The musculoskeletal tissues established general shapes prior to PCW8 and scaled up until PCW12 (**Supp. Fig. 2e** and **4g**) with minor tuning of relative proportions over these weeks, similar to observations in mice[2]. Pseudotime analysis was used to identify molecular cascades underlying early and late stages of chondrocyte maturation (**Supp. Fig. 2b-c, f**). We observed a pre-chondral stage involving downregulation of fibroblast-related (LOX, COL5A1) and perichondrium-related ECM genes (THBS2, COL14A1); followed by a module featuring signaling activation (BMP5, SEMA6A, PDGFRA, SEMA3A) and transcriptional regulation (CDH3, SOX11, GLI3, NFIB) likely corresponding to condensation stages; third, a chondroblast pro-ECM module in which many canonical cartilage markers emerge (SOX9, COL11A1, ACAN, COL9A3, EPYC); and finally a mature phase featuring markers found in specific growth plate zones (UCMA and PRELP, **Supp. Fig. 2f**). Trajectory analysis of myoblasts similarly identified four stages of maturation (**Supp. Fig. 2h**).

To investigate mesenchyme fate decisions, we reduced positional gene expression effects by analysing sample subsets. Tree reconstruction of individual PCW6.5 samples, using RNA velocity to determine the 'root' when possible, produced variable tree topologies (**Supp. Fig. 3a-p**), suggesting region-specific fate decisions among skeletal progenitor cells. In most cases the root was situated nearby cells expressing markers of early mesenchymal progenitors, and other neural crest derivatives. However, trajectory inference in mesenchyme is highly sensitive to input parameters, and can lead to artifacts in which either unlikely cell state transitions are predicted, or that low-quality cells are considered as root; thus, more detailed cell subsets and trajectory inference, paired with results from lineage tracing data, may support more authoritative tree reconstructions in future studies. Nonetheless, reconstructing trees from region-specific datasets with pooled timepoints, using the average age of clusters to identify the root, yielded fairly stable topologies (**Supp. Fig. 3q-t**). In most trees observed, the decision to make skeletal versus non-skeletal cells occurred relatively early, with dermal, perivascular and other stromal cells predicted to arise from a dedicated non-skeletal oligopotent mesenchyme progenitor cell (**Supp. Fig. 3r-t**). However, in the frontonasal tree, a chondrogenic decision was prioritized (**Supp. Fig. 3r-t**). Though these cell samples were not depleted of other germ layers, which can influence root selection and thereby trajectory robustness, these results are consistent with a positional influence on the structure of mesenchyme fate choices. Examining the potential regulatory basis for position-driven cell behavior, we observed numerous DARs specific to nose, eyes, or jaw regions (**Supp. Fig. 4, Supp. Table 2**). Together, these results suggest that human embryonic facial mesenchyme exhibits complex and pattern-dependent transcriptional trajectories during the sampled stages, and that aspects of these spatiotemporal patterns are mirrored in the chromatin accessibility landscape.

Supplementary Note 2: Tissues and structures captured in the spatial atlas

The spatial transcriptomics dataset spans several craniofacial regions with a full view of ears, eyes, nose, tongue (**Extended Data Fig. 3b**), as well as all major mesenchymal tissues such as cartilages and pre-skeletal tissue corresponding to mandible, maxilla, palatal shelves, and the forming teeth with near single-cell resolution (for example, see the fine patterns of vasculature in panel under "blood vessel"). In the caudal section, a clear view of ear cartilage just inferior to trigeminal ganglia and forming pituitary gland was found (**Extended Data Fig. 3c**), and even individual cross-sections of a single nerve fiber could be resolved from gene expression patterns (**Extended Data Fig. 3c**). These data facilitated deducing gene signatures that could be used for anatomy-aware categorization of fibroblast-like mesenchymal cells involved in, for example, development of the palate, eyelids, salivary gland, tongue, or perichondrium (**Extended Data Fig. 4**). Indeed, adjacent mesenchymal clusters within the general embedding mapped to widely distinct facial regions, supporting the notion that positional signature is intertwined with observed transcriptional states (compare extraocular versus salivary mesenchyme, and see overlapping gene signatures marking dental versus lingual mesenchyme, **Extended Data Fig. 4**). Thus, we reasoned that the resolution of the Stereo-seq dataset is suitable for applying an unbiased screening approach to identify potential regulators of positional identity. The resulting list of genes would be hypothesized to be implicated in the generation of human facial pattern and form.

396 spatial genes were identified by comparing Moran's I metric within both transcriptional and anatomical spaces (**Extended Data Fig. 5a-b**, for technical details see *Spatial codes analysis* in the main Methods). While this spatial autocorrelation approach enriches for cell-type independence, a small subset of cell type markers (for instance, COL2A1) were also included due to their extremely high autocorrelation in anatomical space. As an alternate way to screen for cell-type-independent spatial DEGs, we compared chondrocytes obtained from different facial regions (**Extended Data Fig. 5c**), which yielded a list of highly significant nose-specific (ALX1, PAX3, PAX7, SOX11), eye-specific (FOXP1, EBF1), and jaw- specific (MEIS1, MEIS2, SATB2) genes within cartilage cells. Roughly a quarter of the Moran's I spatially autocorrelating genes were found in this list (**Supp. Table 3**), suggesting that the autocorrelation analysis revealed unique region-specific transcriptional identities that emerge from an early stage (at latest PCW6) and that persist throughout critical stages of facial skeletogenesis.

Mapping gene expression patterns across UMAP and anatomical spaces helped classify mesenchymal cells more accurately, but not all signatures were mapped equally well to the Stereo-seq or Visium datasets, or gave ambiguous results. In particular, the gene expression signature from the meningeal cell cluster showed similarities with some nasal and peri-skeletal mesenchyme populations, and many fibroblasts or mesenchymal cell populations generally expressed many of the same marker genes. Thus, the reader is cautioned not to expect all clusters to be found in all of the Stereo-seq sections, and to be wary of signatures with generally low average expression.

Supplementary Note 3: Assigning candidate cis-regulatory elements (CCREs) to target genes

To link promising candidate CREs (cCREs) to target genes, we employed the LinkPeaks function from the Signac package, which screens for correlations between open chromatin and gene expression, using a distance threshold set at 3 Mbp to capture short as well as long-distance cCREs[3]. We found 50,289 unique peaks linked to expression of 6095 specific genes (5759 autosomal protein-coding genes). The vast majority of these linked peaks were classified as distal or intergenic. We refer to the gene-peak pairs having correlated activity patterns across cell populations, as 'links'. Genomic distances from genes to their linked peaks followed a power law distribution with a large tail until the distance cutoff. Most peaks (>94%) were more than 5 kb away from their linked gene (**Supp. Fig. 7a**), with most genes being several hundred kb away from linked peaks on average (**Supp. Fig. 7b**), which is compatible with long-range enhancer interactions. Within intergenic peaks, gene-linked loci showed higher evolutionary conservation than the class-matched background (Wilcoxon rank-sum, BH $q < 5x10^{-5}$; **Supp. Fig. 7c**), which would be expected of regulatory regions. Of note, we observed many peaks linked to multiple genes, which is consistent with simultaneous enhancer regulation of gene groups. Conversely, we observed genes linked to dozens of distinct ATAC peaks (the most highly-linked gene being TWIST2 with hundreds of distinct linked peaks); many of these genes with dozens of linked peaks (including MSX1, PAX1, ALX1, PRRX1, RUNX2, SOX9) have well established roles in craniofacial development and disease in multiple species (labels in **Supp. Fig. 7d**).

Encouraged to find out whether links show molecular features of active enhancers, we performed a series of overlaps with other published datasets. Chromatin peaks were annotated using the 25-state model of chromatin developed by Justin Cotney's lab, based on ChIP-seq measurements of histone modifications from human embryonic craniofacial tissue[4]. The proportion of ChromHMM-annotated enhancers amongst linked peaks was double compared to that of all ATAC peaks, indicating that LinkPeaks enriches for active enhancer states (**Figure 3a, Supp. Fig. 7e**). Further, 442 of 1237 strongly positive VISTA-validated enhancers (35.7%), and 6874 of 13,983 H3K27ac+ craniofacial enhancers previously screened by Axel Visel's lab, overlapped with linked peaks (**Supp. Fig. 7e**). Finally, CAGE-seq data was re-analyzed[5] as an alternate method for annotating cCREs, because CAGE is very reliable for detecting eRNAs, although enhancer prediction by signal directionality alone may give lower sensitivity compared to ATAC-seq and ChIP-seq approaches[6]. In total, 59,260 CAGE+ regions were annotated, and CAGE+ regions overlapped with 8762 links (**Supp. Fig. 7e**). We were also curious whether some of the identified enhancers were human-specific. Comparing the linked peaks with the top 5000 enhancers in primate neural crest cells, and with the top 1000 human-biased enhancers in neural crest cells[7] revealed dozens of overlaps, some of which were targeting genes specifically enriched in musculoskeletal tissues (**Supp. Table 2**). Prominent examples of genes linked to these enhancers include *MXRA8* and *UST* (found in facial bones), *ZNF106* (enriched in muscle), *COL24A1*, *SEMA3A*, *CALD1*, and *SULF1* (perichondral and some periocular mesenchymal regions) and *SGCD* (nasal cartilage). Notably, many linked peaks identified to overlap with enhancers were also region-specific DARs (**Supp.Table 2**), and 'spatial' genes of the nose region demonstrated significant enrichment of enhancers (hypergeometric test for CAGE FDR 1.37e-4,

H3K27ac+ FDR 1.99e-5). Together, these overlaps suggest that our cCRE list represents active developmental enhancers in facial tissues.

Mapping these link distances per chromosome revealed a clustering of links stacking onto specific loci (**Supp. Fig. 7**), with many links with similar distances clustering together. The link clustering organization resembled chromatin looping described to occur during enhancer interactions, suggesting that links represent regulatory interactions occurring on the scale of TADs. Overlap with published chromatin conformation capture (Hi-C) measurements of human embryonic craniofacial tissue[8] revealed that distal linked peaks were primarily found on the borders of TADs, and identified over 3000 intergenic links for which looping between linked peak and target gene was observed. However, most links crossed TAD boundaries instead of residing within a single TAD, and as such, TAD directionality was not predictive of link directionality (**Supp. Fig. 8** and Methods for details). Together these results provide provisional support for thousands of cCREs, and motivates future studies of how genome compartmentalization dynamics influences facial enhancer function.

To discover meaningful cis-regulatory interactions that influence human facial shape, we then overlapped links with craniofacial-associated genetic variants. Following the approach of a recent study[9], roughly 29,000 lead SNPs and proxy variants in linkage disequilibrium (LD) from lead SNPs were gathered from > 40 GWAS studies pertaining to different aspects of craniofacial variation and pathology. We found > 500 unique linked peaks contained face-related SNPs (**Supp. Table 2**), and of these, > 200 additionally overlapped with enhancers according to CAGE and/or CHIP data (**Supp. Table 2**). Among these face-associated putative enhancers, we observed links to ‘spatial TFs’ such as ALX1, PAX1, PAX3, PAX7, PAX9, EMX2, HAND2, PRRX1, MAFB, MSX1, MSX2, OSR1, OSR2, TWIST2, PITX2, and BARX1. Overall, multiple unbiased large-scale screens were overlapped to generate a high-confidence list of cCREs and predicted interacting genes (either as targets or as regulators). The data points to a model in which variation in enhancer sequences, in particular those linked to spatial TFs, might drive human facial individuality by participating in the gene networks that drive embryonic facial patterning. Future studies should focus on comprehensively validating the regulatory logic of these human cCREs *in vivo* using vertebrate models.

Supplementary Note 4: Variants in enhancers of key developmental disease genes may drive facial diversity

There are hundreds of genes for which the connection to heritable craniofacial pathologies have been established[10-14]. Previous studies also found an overlap in the genetic architectures for normal-range philtrum width and cleft palate, suggesting that similar gene sets may drive disease and variation[10,15]. In line with this, we found significant disease enrichment in the lists of a) genes within 500 kb of, and b) genes linked to, SNPs associated with normal variation (**Supp. Fig. 10a-b, Supp. Table 5**), partly driven by an enrichment of 'spatial' genes in both disease and variation lists (**Supp. Fig. 10**, **Supp. Table 5**). This result suggests that enhancers targeting key loci serve as a vehicle for facial diversity. Supporting this, link status, enhancer annotation, and variant burden synergistically elevates heterozygosity ratio[16] of distal intergenic peaks, in a manner partly corresponding with Neanderthal introgression (**Supp. Fig. 10c-d**). Overall, these specific regions appear to be hotspots for generating genetic diversity in modern human populations, despite stronger evolutionary conservation for enhancers and linked peaks. Notably, key disease/variation genes[10] exhibit diverse expression profiles in facial cells, including many genes that are highly expressed in most or all mesenchymal clusters (**Supp. Fig. 11**).

As a complementary mechanism, gene imprinting can in principle create parent-of-origin dosage differences that modulate additive effects at facial-variation loci. We examined the expression profile of maternal and paternal imprinted genes (**Supp. Fig. 12**), which have an established link to sexual dimorphism but also craniofacial anomalies (Silver-Russell syndrome, Temple syndrome, among others). As many imprinted genes reside near facial variation SNPs and are expressed broadly in craniofacial mesenchymal cells, complete and partial allele-specific silencing may provide an additional layer of epigenetic diversification, which can shape cell identity and contribute to the heritability of facial traits[17-19].

Thus, the present study presents evidence that, in agreement with previous studies, enhancers seem to be critical for human facial variation. Further, enhancer multiplicity around key positional genes might explain pleiotropy via combinatorial regulatory inputs. At MSX1, distinct cCREs with different accessibility profiles harbor trait-specific variants, indicating that composite enhancers parcel a gene's influence across tissues and facial subregions. Such modular regulation could predispose a coding gene to distinct quantitative effects (normal shape variation) and qualitative defects (tooth agenesis, cleft palate) depending on which cell state, time, and place is affected by genetic variation. While enhancers via cell type and region-specific activity profiles subtly influence gene expression patterns of a protein, coding mutations can simultaneously disrupt protein functions body-wide to manifest as pathology. We speculate that evolutionary exploration of facial form may have occurred through modular and redundant enhancer systems, where certain levels of variation can be tolerated and even favored.

Supplementary Note 5: Supplementary Discussion

Human facial form arises from a cascade in which genetic variation shapes enhancer activity, gene expression, and cell fate decisions within specific embryonic niches. This multi-layered process results in a species-specific morphology that also defines personal identity. Finding out why certain variants cause disease, generate individuality, or, in some cases, are associated with both pathology as well as variation, requires an integrative framework to connect these layers. Transcriptomics identifies active genes but fails to identify regulatory logic; chromatin maps highlight enhancers but not their context; spatial profiling reveals positional codes but lacks genetic resolution; and GWAS provides statistical associations but not cellular mechanisms. As a consequence, accurate modeling of faces from DNA remains a challenge[20]. In this study we use a multimodal atlas approach, integrating single-cell transcriptomics, multi-omics (snRNA/ATACseq), spatial transcriptomics, anatomical reconstructions, and epigenomic assays between the key stages of weeks 6 and 11 of human embryos, a critical time window when facial structure progressively emerges[21]. This intersectional approach facilitates assigning variants to enhancers, linking enhancers to genes, and situating gene activity across defined cell populations and spatial niches, which will help uncover general cis-regulatory grammars driving facial individuality and disease [22-24]. These results provide a foundation for the identification of novel enhanceropathies from unsolved craniofacial anomalies lacking coding mutations.

Our biology cannot always be inferred from animal models, particularly for traits that may be shaped by human-specific selection. Phenotypic variation can carry social information, and certain facial traits may have been specifically selected for in humans[25]. Also, many congenital craniofacial disorders are replicated poorly in model organisms[26-30]. This may partly reflect species differences in developmental tempo[31]. For example, CNCCs migrate during the fourth week in humans[32], between days 8-10.5 in mice[33], around 2 days in chick[34], and between 15-19 hours in zebrafish[35]. Furthermore, the large human brain supplies unique biomechanical and signaling inputs to influence facial growth[36]. While it is apparent that mesenchyme, cartilage and bone play conserved roles in establishing facial morphology [37,38], the relative contributions of various cell states to human facial traits have not been well-characterized. Understanding this could explain why certain syndromes affect some parts of the skeleton while sparing others[39]. To further understand human-specific biology, it will be important to compare this dataset with similar atlases from mice and other model organisms.

A central challenge that remains is deciphering how the anterior face encodes positional identity of mesenchymal cells. Unlike the trunk and hindbrain, which rely on Hox codes, the upper and midface develop without a canonical Hox system. Only a handful of factors such as ALX, DLX, MSX, HAND, and endothelin signaling were previously known to act regionally based on studies in mice. However, high-resolution profiling had not yet been performed to generate a comprehensive list of candidate human facial patterning regulators. Using unbiased spatial clustering approaches [40], applied to highly resolved spatial transcriptomics data, we identified hundreds of ‘spatial’ genes, including dozens of secreted factors and TFs. In addition to the above-mentioned factors, the gene list includes established patterning TFs with proposed spatial codes in vertebrates, such as PAX domains along the anterior-posterior axis of cranial neuroectoderm[41], and FOX domains that contribute to diversification of ectomesenchyme in

pharyngeal arches[42]. In addition, spatial TFs were identified that have not been previously associated with roles in patterning, such as ZNF503 and ZNF804a, as well as patterning factors that have not been implicated in craniofacial development, such as PROX1 and HES6. Among the spatial TFs, many 1) were co-expressed in specific regional domains, 2) exhibited binding site motif enrichment and regulon activity within multiple mesenchymal cell types, and 3) were highly enriched for variation and disease traits. Thus, even slight shifts in their expression provide a plausible basis for fine-scale human variation, and lend credence to the idea that pathological and natural variation exist on a shared spectrum of developmental outcomes, based on underlying genetic architectures that converge on similar gene sets and/or cell types.

The intersection between normal-range individuality and pathology raises fundamental questions: to what extent does evolution reuse the same loci and genes both to generate variation and to underlie disease, and how often are these pools distinct? Given the many genes linked to SNP-bearing peaks, enhancer regulation seems a likely explanation for why syndrome-associated genes are located near common noncoding variants that tune facial measurements in the general population. Our results suggest that facial trait specificity can be explained in part by expression profiles of patterning genes in craniofacial mesenchymal cells, offering a mechanistic rationale for how noncoding variants translate into region-specific adult features. ALX1 locus exemplified this logic well, as an upper face biased TF sitting near upper-face variants in a cCRE with specific accessibility in nasal cells.

Overall, the data presented in this study is anticipated to be useful for research across numerous fields. We hope to facilitate comparative studies by integrating this atlas with data from mouse, chick, zebrafish and other models, as this may uncover mechanisms linking facial phenotypic evolvability with adaptation to diverse ecological niches[43], either via gene duplications[44] or mutations [45]. Because organoid and tissue engineering approaches are still emerging and will develop increasingly sophisticated human facial organoids, we anticipate our atlas will be useful for benchmarking engineered cells, both in vitro and in regeneration settings[46,47]. Furthermore, the regulatory elements identified herein should be compared with genomic data from Neanderthal and Denisovan populations to explore the evolution of facial traits in extinct hominids[48]. Finally, this atlas has been organized as an accessible online resource with intuitive navigation and analysis tools (via CELLXGENE, see data availability statement), enabling use by non-computational specialists such as experimental biologists, geneticists, paleoanthropologists, clinicians, and the broader research community.

Supplementary Methods

Regional dissections

Mandibular prominence was isolated by an incision from the oral commissure to the temporomandibular joint, with connecting soft tissue removed to separate the lower jaw and tongue from the upper face. Frontonasal prominence region was defined by excising protruding nasal tissue between alar-facial grooves. Forehead region was obtained by removing flaps of cranial tissue superior to the eyes and nasion. The eye region consisted of the globes with surrounding orbital mesenchyme and adjacent maxillary tissue. For one PCW6 MO specimen only posterior (lower jaw and tongue) versus anterior (upper face) regions were distinguished. For Visium samples, craniofacial samples were bisected along the midline, with one half frozen for spatial analysis, and the other processed for cell isolation. A subset of samples was processed directly without regional separation.

Cage-seq library preparation and sequencing

The raw CAGE data generated during this study has now been published [5]. RNA was extracted from the frozen samples using RNeasy mini kits (Qiagen, Germany). 20mg of tissue was homogenized with liquid nitrogen and mixed with a lysis buffer. Lysate samples were collected in clean tubes and incubated with a proteinase K solution for 10 minutes at 55°C followed by centrifugation at 14 000rpm. Then an equal volume of 96% ethanol was added to the supernatant in order to preserve short RNA fragments and applied to a flow column. Next, the samples were washed with RPE wash buffer two times, and the final isolation steps (drying and elution) were carried out according to the manufacturer's standard protocol. The RNA concentration and integrity were evaluated by a fluorometer (Qubit 3.0; Thermo Scientific) and by capillary electrophoresis (Bioanalyzer 2100, Agilent, USA), respectively. All samples had RIN value ≥ 7. Libraries were constructed from 5 µg of RNA according to nAnT-iCAGE (non-Amplified non-Tagging Illumina Cap Analysis of Gene Expression) protocol [49] using nAnT671 iCAGE Library Preparation kit (DNAform, Japan) and SuperScript III Reverse Transcriptase (Invitrogen, USA). The concentration of the obtained libraries was measured on a Qubit 3.0 fluorometer (Thermo Scientific, USA) and then validated using real-time PCR (KAPA Library Quantification Kits Illumina, KAPA Biosystems, South Africa) before sequenced on the HiSeq 2500 platform (Illumina, USA) in single end mode with a length of 50 bp. The data analysis was slightly modified to more sensitively detect eRNA. Instead of localization based masking[5], we relied on the filtering of CAGE reads by unaligned G on the 5' end. This gives better resolution, while the calling formula (by expression directionality) remains the same. A 100 bp buffer was added to the upper and lower bounds of all CAGEs to increase sensitivity when overlapping other genomic elements (as in previous studies[50]).

Distance weighted trait enrichment scores

We selected a significance threshold of -log10(p) > 8.43 by scanning cutoffs to maximize the fraction of variants mapping to a single trait. For each trait, SNPs passing this threshold were expanded to ±5, ±50, and ±500 kb windows to collect proximal genes, yielding trait-specific gene panels per distance. Distance-weighted trait enrichment across cell types was calculated by 1) average gene expression across cells per cluster 2) sum cluster averages across the genes in a

panel 3) sum the results across distance thresholds. Gene-by-cluster values were row-z-scored, converted to one-sided p-values, FDR-adjusted across the matrix, and significance marked at FDR < 0.05.

Neanderthal introgression, evolutionary conservation, and heterozygosity ratios

Heterozygosity ratios, or R, in modern human superpopulations, was found using data from the 1000 Genomes project, by calculating the number of heterozygous sites divided with the number of homozygous non-reference sites. Evolutionary conservation was tested via PhastCons100way (hg38) conservation scores imported from UCSC, calculating the mean score across each peak interval. Neanderthal scores were calculated by overlapping peaks with published Neanderthal introgression intervals. For each 1000 Genomes superpopulation (AFR, AMR, EAS, EUR, SAS), the number of intersecting base pairs with peaks per individual were computed, and base-pair frequency was calculated as the sum of each individual's overlap score divided by the number of individuals, where each individual's score was the total overlapping base pairs divided by peak length. Results were merged across superpopulations, with missing values set to zero, and a global Neanderthal introgression score derived as the mean base-pair frequency across non-AFR populations. To test for differences in the computed scores, distal intergenic peaks within ±3 Mbp of a TSS were stratified into linked enhancers, all linked distal intergenic peaks, and all distal intergenic peaks. Conservation scores (PhastCons), Heterozygosity ratios, and Neanderthal scores were summarized for each group, and linked versus non-linked distal intergenic regions were compared using Welch's t-test, Wilcoxon rank-sum with Hodges–Lehmann location shift, and Cohen's d. A three-group comparison (A vs B vs C) was performed with Kruskal–Wallis (ε^2 effect size), followed by pairwise Wilcoxon tests with BH correction and Hodges–Lehmann estimates.

Gene set enrichment

Testing of molecular functions and phenotypes associated with spatial genes in Figure 2 was performed using the online tool Enrichr. However, for comparing various gene lists for their disease enrichment, the Human Phenotype Ontology (HPO) database was used. Genes associated with abnormalities of the head and neck (HP:0000152 and all descendents) were extracted from the HPO to generate the list of craniofacial pathology genes. Protein-coding autosomal genes were defined from Ensembl GRCh38 (EnsDb v86) by selecting entries with biotype = "protein_coding" on chromosomes 1–22 (excluding X, Y, MT) and collapsing to unique gene identifiers. Enrichment among disease, spatial, linked, and variation-associated gene lists was tested using two-sided Fisher's exact tests on 2×2 contingency tables, performed separately within two background sets: all protein-coding autosomal genes and the subset of genes linked to ATAC peaks in our multi-omics dataset.

Mapping nose-jaw SNP specificity to gene expression localization

Lead SNPs from White 2021[51] were used. Per SNP, geometric means of -log10(p) values were aggregated across segments affecting the nose or jaw regions, and the difference was computed to yield a nose-jaw contrast score per SNP. SNPs were mapped to genes within ±5, 50, and 500 kb, and for each gene the mean SNP nose–jaw contrast was calculated. Scores from the three window sizes were combined into a single per-gene average. Per gene, and per cell cluster, the

precomputed nose–jaw SNP score (score_avg) was plotted against differential expression levels in nose versus jaw samples. For each cluster (columns 2–57), a linear model was fitted (expression difference ~ SNP score), recording R2, p-value, slope, and the Pearson correlation between variables. P-values were FDR-adjusted (Benjamini–Hochberg). Cluster-wise p-values were tested for following U(0,1) using a one-sample Kolmogorov–Smirnov test and directional bias was assessed with a binomial test on the sign of correlations (noting clusters are not strictly independent).

HIC analysis

Previously identified links were overlapped with the publicly available Hi-C data of human embryonic craniofacial tissue from [8]. Already precomputed Hi-C matrix was obtained and further analysed using cooler (v 0.10.2) and cooltools (v 0.7.1) [52,53]. Average contact frequency was computed with cooltools.expected_cis with default parameters without smoothing. Links and genes coordinates were coarsed to Hi-C bins with bioframe (v 0.7.2)[54]. Links closer than 200kb to the gene were called “proximal”, and were called “distal” otherwise. For proximal links on-diagonal link-centered 200kb-wide observed-over-expected pileups were computed using cooltools.pileup. For distal links off-diagnonal observed-over-expected pileups with 200kb-window were computed. For both proximal and distal links, pileups containing more than 20% missing values of Hi-C matrix were excluded from the analysis. Pileups of proximal links were clustered using the k-means algorithm with k=6 from scikit-learn (v 1.3.2). Pileups of distal links were classified into two groups, with or without loop, based on the following criteria. Links were considered loop-containing when for their N*x*N pileup the average observed-over-expected ratio in the central 2N/3 region (“inner region”) is higher than 1.2 and “inner region” mean divided by the mean of N/4-wide corner regions is higher than 1. TAD boundaries were identified using cooltools.insulation with 100kb window size. Links were considered crossing TAD boundary if there is at least one strong TAD boundary located between link end and gene start, or *vice versa* for the links in (-)-strand. PhastCons score was compared for each links cluster between links crossing and not crossing TAD boundary with the Mann-Whitney U Test from the pingouin (v 0.5.5) [55]. Hi-C maps and links for selected example regions were visualised using pyGenomeTracks (v 3.9)[56].

i-GONAD

All mouse experiments were carried out in compliance with the Swedish Animal Welfare Act and approved by the respective government offices in Gothenburg, Sweden using approximately 10-week old RjOrl:SWISS mice from Janvier labs. i-GONAD was carried out as described by Gurumurthy *et al*[57]. Briefly, the estrous cycle of CD1 mice was determined by visual inspection [58] and chosen animals were mated at 4 pm on the day before surgery. The next morning, mice were checked for vaginal plugs and i-GONAD was carried out at 3 pm on the same day. The ampullae of the oviducts containing the one-cell-stage embryos were injected with genome editing mix, containing 30 µM gRNA, 0.8 µg/µL ssDNA, 0.005% (w/v) Fast Green FCF (Roth), and 6.1 µM Cas9 diluted in Opti-MEM (Gibco). This was followed by in situ electroporation to facilitate uptake of the components. One founder male was successfully created using i-Gonad. Backcrossing revealed germline transmission of the inserted sequence, albeit transmission rate did not follow

mendelian distribution and only 7.8% of offspring carried the mutation. Crossing the founder with heterozygous offspring showed mendelian transmission of mutation in backcrossed animals.

Genotyping and Sanger Sequencing

Genotyping primers chosen to flank the entire insert as well as one reverse primer for the wildtype sequence and one for the scrambled insert. Two more primer sets S1 and S2 were designed to confirm proper insertion of the entire scrambled sequence. Primers were ordered from Eurofins Genomics (Gothenburg, Sweden). In addition, the reverse transcript of the genotyping mutant primer and the mutant primer were used for Sanger sequencing. Confirmation of germline transmission was performed via S1 and S2 primers. Confirmation of complete mutant template insertion in founder through Sanger sequencing. Genotyping was performed by PCR using primer pairs that used two different reverse primers specific for wildtype and scrambled sequence and GoTaq® Hot Start Polymerase (Promega, Madison, Wisconsin, USA). PCR conditions were optimized for each allele by gradient PCR. Sequences of genotyping primers are shown in Supplementary Table 6. For Sanger sequencing, PCR was performed using primer pairs that flanked the homology arms of the knock-in DNA sequences and GoTaq® Hot Start Polymerase (Promega, Madison, Wisconsin, USA). The PCR product was purified using PCR purification kit (Qiagen, Hilden, Germany) and sent in using two primers from the middle of the scrambled sequence out. Primers and purified PCR were mixed and sequenced at Eurofins Genomics (Gothenburg, Sweden).

Supplementary Note References

1. Blain, R. *et al.* A tridimensional atlas of the developing human head. *Cell* **186**, 5910-5924 e17 (2023).
2. Kaucka, M. *et al.* Oriented clonal cell dynamics enables accurate growth and shaping of vertebrate cartilage. *eLife* **6**, e25902 (2017).
3. Ma, S. *et al.* Chromatin Potential Identified by Shared Single-Cell Profiling of RNA and Chromatin. *Cell* **183**, 1103-1116.e20 (2020).
4. Wilderman, A., VanOudenhove, J., Kron, J., Noonan, J.P. & Cotney, J. High-Resolution Epigenomic Atlas of Human Embryonic Craniofacial Development. *Cell Rep* **23**, 1581-1597 (2018).
5. Xie, M. *et al.* The level of protein in the maternal murine diet modulates the facial appearance of the offspring via mTORC1 signaling. *Nature Communications* **15**, 2367 (2024).
6. Agrawal, S., Kanamaru, E., Saito, Y., Ishikawa, F. & de Hoon, M. Cell type-dependent directional transcription at enhancers. *NAR Genomics and Bioinformatics* **7**, lqaf007 (2025).
7. Prescott, Sara L. *et al.* Enhancer Divergence and cis-Regulatory Evolution in the Human and Chimp Neural Crest. *Cell* **163**, 68-83 (2015).
8. Wilderman, A. *et al.* A distant global control region is essential for normal expression of anterior HOXA genes during mouse and human craniofacial development. *Nature Communications* **15**, 136 (2024).
9. Rajderkar, S.S. *et al.* Dynamic enhancer landscapes in human craniofacial development. *Nature Communications* **15**, 2030 (2024).
10. Naqvi, S. *et al.* Decoding the Human Face: Progress and Challenges in Understanding the Genetics of Craniofacial Morphology. *Annual Review of Genomics and Human Genetics* **23**, 383-412 (2022).
11. Bartzela, T.N., Carels, C. & Maltha, J.C. Update on 13 Syndromes Affecting Craniofacial and Dental Structures. *Frontiers in Physiology* **8**(2017).
12. Cha, S. *et al.* Identification of five novel genetic loci related to facial morphology by genome-wide association studies. *BMC Genomics* **19**, 481 (2018).
13. Zhang, Y.-B. *et al.* Genome-wide association study identifies multiple susceptibility loci for craniofacial microsomia. *Nature Communications* **7**, 10605 (2016).
14. Mukhopadhyay, N. *et al.* Genome-Wide Association Study of Non-syndromic Orofacial Clefts in a Multiethnic Sample of Families and Controls Identifies Novel Regions. *Front Cell Dev Biol* **9**, 621482 (2021).
15. Indencleef, K. *et al.* The Intersection of the Genetic Architectures of Orofacial Clefts and Normal Facial Variation. *Frontiers in Genetics* **12**(2021).
16. Samuels, D.C. *et al.* Heterozygosity Ratio, a Robust Global Genomic Measure of Autozygosity and Its Association with Height and Disease Risk. *Genetics* **204**, 893-904 (2016).
17. Eggermann, T. *et al.* Imprinting disorders: a group of congenital disorders with overlapping patterns of molecular changes affecting imprinted loci. *Clinical Epigenetics* **7**, 123 (2015).
18. Tanaka, K. *et al.* Paternally expressed gene 3 (Pw1/Peg3) promotes sexual dimorphism in metabolism and behavior.
19. Carli, D., Riberi, E., Ferrero, G.B. & Mussa, A. Syndromic Disorders Caused by Disturbed Human Imprinting. *J Clin Res Pediatr Endocrinol* **12**, 1-16 (2020).
20. Sero, D. *et al.* Facial recognition from DNA using face-to-DNA classifiers. *Nature Communications* **10**, 2557 (2019).

21. Som, P.M. & Naidich, T.P. Illustrated Review of the Embryology and Development of the Facial Region, Part 2: Late Development of the Fetal Face and Changes in the Face from the Newborn to Adulthood. *American Journal of Neuroradiology* **35**, 10 (2014).
22. Zhang, B. *et al.* A human embryonic limb cell atlas resolved in space and time. *Nature* (2023).
23. Bonetti, A., Kwon, A.T., Arner, E. & Carninci, P. Analysis of Enhancer-Promoter Interactions using CAGE and RADICL-Seq Technologies. *Methods Mol Biol* **2351**, 201-210 (2021).
24. Hao, Y. *et al.* Integrated analysis of multimodal single-cell data. *Cell* **184**, 3573-3587 e29 (2021).
25. Sheehan, M.J. & Nachman, M.W. Morphological and population genomic evidence that human faces have evolved to signal individual identity. *Nature Communications* **5**, 4800 (2014).
26. Ng, C.M. *et al.* TGF-beta-dependent pathogenesis of mitral valve prolapse in a mouse model of Marfan syndrome. *J Clin Invest* **114**, 1586-92 (2004).
27. Carta, L. *et al.* Fibrillins 1 and 2 perform partially overlapping functions during aortic development.
28. Gardiner, K., Fortna, A., Bechtel, L. & Davisson, M.T. Mouse models of Down syndrome: how useful can they be? Comparison of the gene content of human chromosome 21 with orthologous mouse genomic regions. *Gene* **318**, 137-147 (2003).
29. Ishii, M. *et al.* Combined deficiencies of Msx1 and Msx2 cause impaired patterning and survival of the cranial neural crest. *Development* **132**, 4937-4950 (2005).
30. Goodwin, A.F., Kim, R., Bush, J.O. & Klein, O.D. Chapter Seventeen - From Bench to Bedside and Back: Improving Diagnosis and Treatment of Craniofacial Malformations Utilizing Animal Models. in *Current Topics in Developmental Biology*, Vol. 115 (ed. Chai, Y.) 459-492 (Academic Press, 2015).
31. Houpt, M.I. Growth of the craniofacial complex of the human fetus. *American Journal of Orthodontics* **58**, 373-383 (1970).
32. Betters, E., Liu, Y., Kjaeldgaard, A., Sundström, E. & García-Castro, M.I. Analysis of early human neural crest development. *Dev Biol* **344**, 578-92 (2010).
33. Adameyko, I. *et al.* Sox2 and Mitf cross-regulatory interactions consolidate progenitor and melanocyte lineages in the cranial neural crest. *Development* **139**, 397-410 (2012).
34. Pettway, Z., Guillory, G. & Bronner-Fraser, M. Absence of neural crest cells from the region surrounding implanted notochords in situ. *Dev Biol* **142**, 335-45 (1990).
35. Kamenev, D. *et al.* Schwann cell precursors generate sympathoadrenal system during zebrafish development. *Journal of Neuroscience Research* **99**, 2540-2557 (2021).
36. Marcucio, R., Hallgrimsson, B. & Young, N.M. Facial Morphogenesis: Physical and Molecular Interactions Between the Brain and the Face. *Curr Top Dev Biol* **115**, 299-320 (2015).
37. Damas, S., Cordón, O. & Ibáñez, O. Relationships Between the Skull and the Face for Forensic Craniofacial Superimposition. in *Handbook on Craniofacial Superimposition: The MEPROCS Project* (eds. Damas, S., Cordón, O. & Ibáñez, O.) 11-50 (Springer International Publishing, Cham, 2020).
38. Gietzen, T. *et al.* A method for automatic forensic facial reconstruction based on dense statistics of soft tissue thickness. *PLOS ONE* **14**, e0210257 (2019).
39. Iyyanar, P.P.R., Wu, Z., Lan, Y., Hu, Y.-C. & Jiang, R. Alx1 Deficient Mice Recapitulate Craniofacial Phenotype and Reveal Developmental Basis of ALX1-Related Frontonasal Dysplasia. *Frontiers in Cell and Developmental Biology* **10**(2022).
40. Long, Y. *et al.* Spatially informed clustering, integration, and deconvolution of spatial transcriptomics with GraphST. *Nature Communications* **14**, 1155 (2023).

41. Edens, B.M. & Bronner, M.E. Making sense of vertebrate senses from a neural crest and cranial placode evo-devo perspective. *Trends in Neurosciences* **48**, 213-226 (2025).
42. Xu, P. *et al.* Fox proteins are modular competency factors for facial cartilage and tooth specification. *Development* **145**(2018).
43. Usui, K. & Tokita, M. Creating diversity in mammalian facial morphology: a review of potential developmental mechanisms. *EvoDevo* **9**, 15 (2018).
44. Sears, K.E., Goswami, A., Flynn, J.J. & Niswander, L.A. The correlated evolution of Runx2 tandem repeats, transcriptional activity, and facial length in carnivora. *Evol Dev* **9**, 555-65 (2007).
45. Schoenebeck, J.J. *et al.* Variation of BMP3 contributes to dog breed skull diversity. *PLoS Genet* **8**, e1002849 (2012).
46. Lin, W., Wang, M., Xu, L., Tortorella, M. & Li, G. Cartilage organoids for cartilage development and cartilage-associated disease modeling. *Frontiers in Cell and Developmental Biology* **11**(2023).
47. Borrelli, M.R., Hu, M.S., Longaker, M.T. & Lorenz, H.P. Tissue Engineering and Regenerative Medicine in Craniofacial Reconstruction and Facial Aesthetics. *J Craniofac Surg* **31**, 15-27 (2020).
48. Wroe, S. *et al.* Computer simulations show that Neanderthal facial morphology represents adaptation to cold and high energy demands, but not heavy biting. *Proceedings of the Royal Society B: Biological Sciences* **285**, 20180085 (2018).
49. Murata, M. *et al.* Detecting expressed genes using CAGE. *Methods Mol Biol* **1164**, 67-85 (2014).
50. Andersson, R. *et al.* An atlas of active enhancers across human cell types and tissues. *Nature* **507**, 455-461 (2014).
51. White, J.D. *et al.* Insights into the genetic architecture of the human face. *Nature Genetics* **53**, 45-53 (2021).
52. Abdennur, N. & Mirny, L.A. Cooler: scalable storage for Hi-C data and other genomically labeled arrays. *Bioinformatics* **36**, 311-316 (2020).
53. Open2C *et al.* Cooltools: enabling high-resolution Hi-C analysis in Python. *bioRxiv*, 2022.10.31.514564 (2022).
54. Open2C *et al.* Bioframe: operations on genomic intervals in Pandas dataframes. *Bioinformatics* **40**, btae088 (2024).
55. Vallat. Pingouin: statistics in Python. Vol. 3 1026 (Journal of Open Source Software, 2018).
56. Lopez-Delisle, L. *et al.* pyGenomeTracks: reproducible plots for multivariate genomic datasets. *Bioinformatics* **37**, 422-423 (2021).
57. Gurumurthy, C.B. *et al.* Creation of CRISPR-based germline-genome-engineered mice without ex vivo handling of zygotes by i-GONAD. *Nat Protoc* **14**, 2452-2482 (2019).
58. Byers, S.L., Wiles, M.V., Dunn, S.L. & Taft, R.A. Mouse estrous cycle identification tool and images. *PLoS One* **7**, e35538 (2012).

Supplementary Figure 1 - *Cell type specific accessibility of chromatin*

Heatmap of differentially accessible regions (DARs) across cell clusters. Columns represent annotated cell clusters (fine-grained). Values are row-scaled $\log_2$FC, with color indicating relative accessibility (blue = decreased, red = increased).

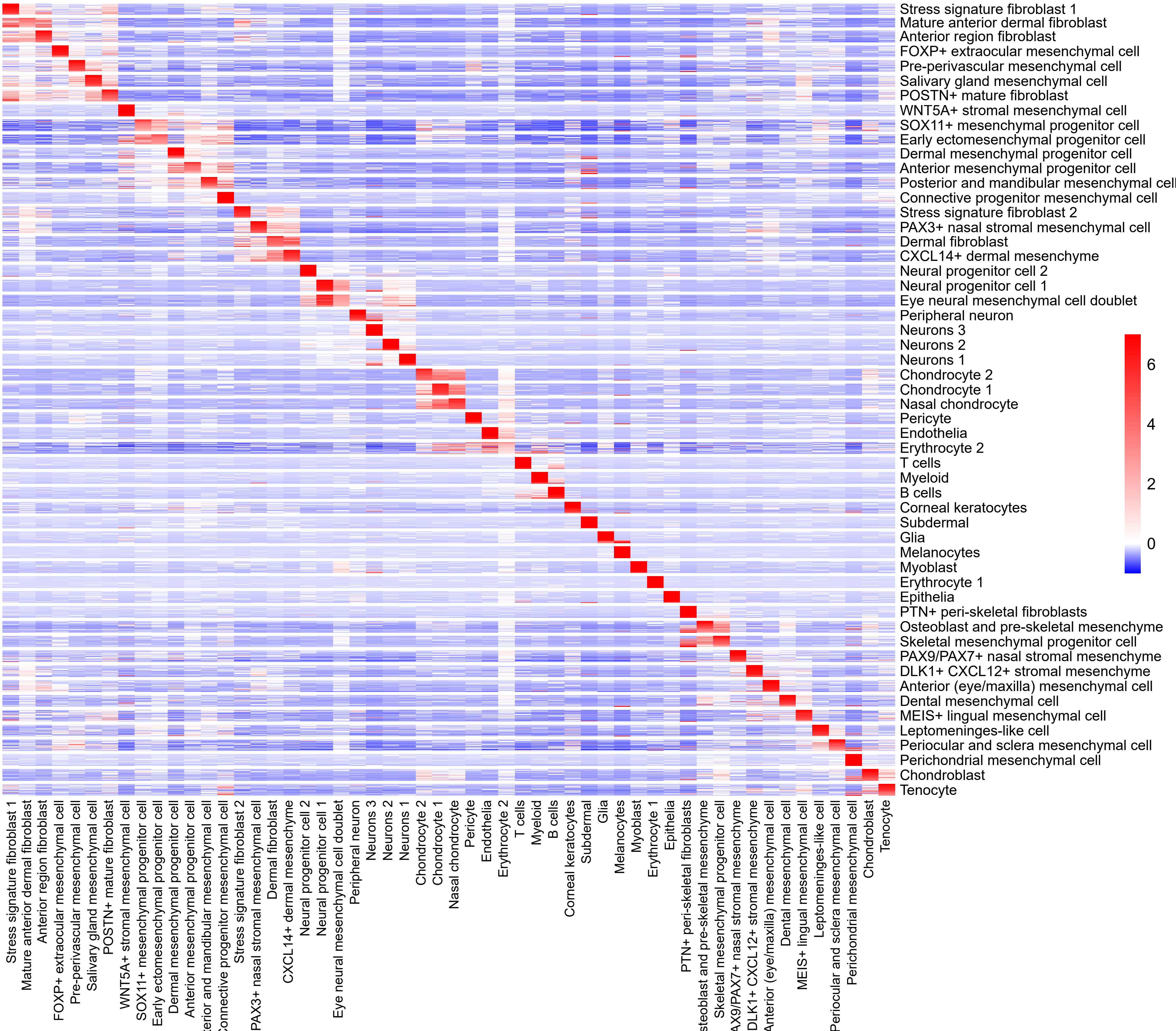

Supplementary Figure 1

Supplementary Figure 2 - *Growth and developmental trajectories of musculoskeletal tissues in the human embryonic face*

- **a.** Micro-CT scans of human face at week PCW8, PCW9, and PCW12 with musculoskeletal structures volumetrically segmented.
- **b.** General embedding.
- **c.** Gene signature for isolating pre-chondral cells and chondrocytes.
- **d.** Gene signature for isolating myoblasts and myocytes.
- **e.** Front, side, and top view for each cartilage element at the stages PCW8-PCW12.
- **f.** SCFATES trajectory of the timeline of chondrocyte differentiation from prechondrogenic mesenchyme.
- **g.** Front, side, and top view for each muscle at the stages PCW8-PCW12.
- **h.** SCFATES trajectory of the timeline of myocyte differentiation from mesenchyme.

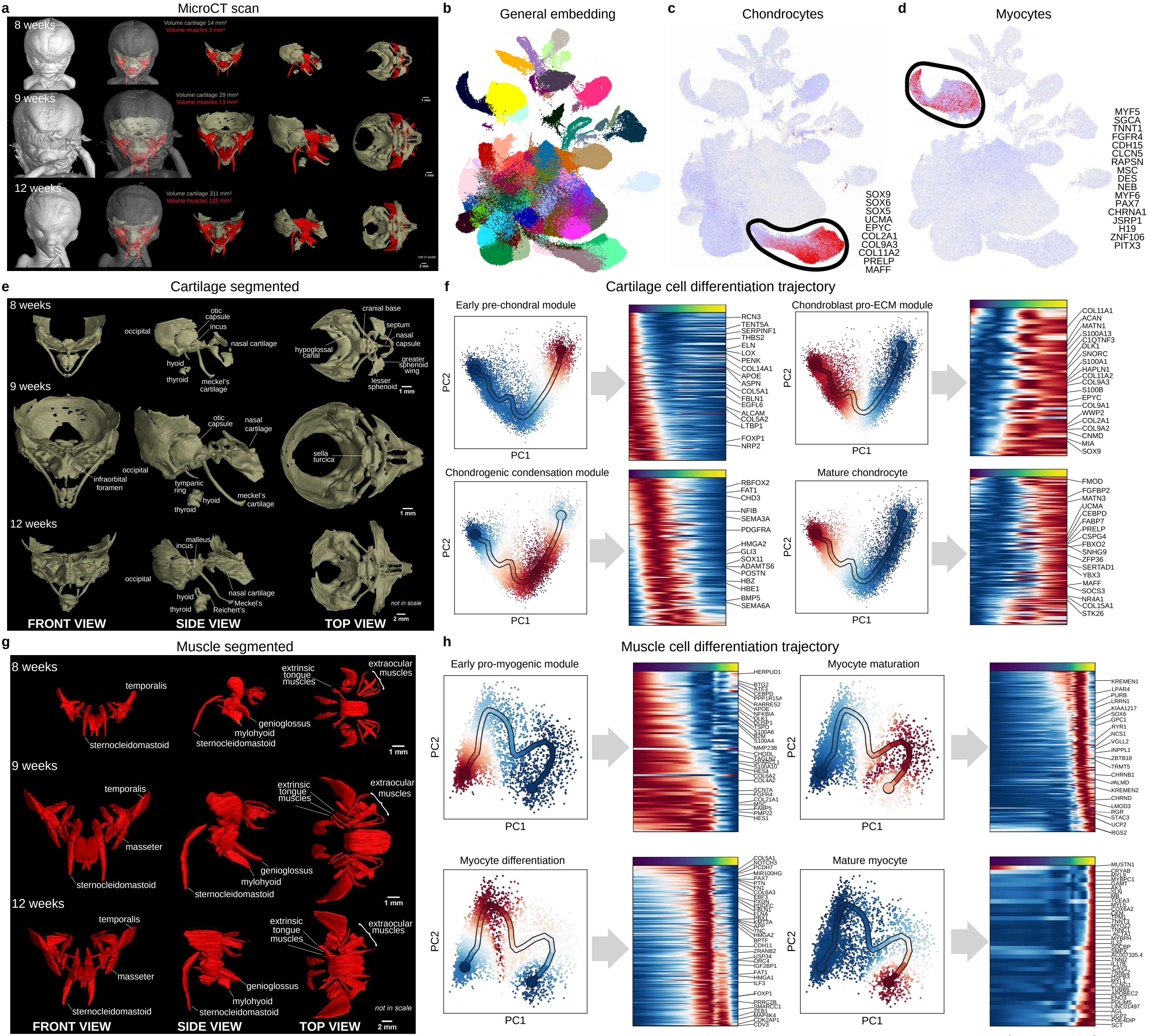

Supplementary Figure 2

Supplementary Figure 3 - *Region-specific hierarchies of skeletal cell fate decisions in human embryonic facial tissue* **(a-d)** RNA velocity was performed on mesenchymal subsets from individual samples; PCW6 frontonasal prominence (a), PCW6 mandibular region (b), PCW6 periocular region (c), and PCW6 periotic region (d). Cells are color-coded by Leiden clusters with adjacent annotations. Arrows indicate predicted transcriptional flow. **(e-h)** Force atlas embedding of the frontonasal (e), mandibular (f), periocular (g), and periotic (h) mesenchymal subsets. Terminal nodes are labeled according to the annotations in the panels above. **(i-l)** Transcriptional inferred trajectories according to latent time. Root nodes were selected with a combination of Velocity and CYTOTRACE. Trajectory is shown for frontonasal (i), mandibular (j), periocular (k), and periotic (l) mesenchymal subsets. **(m-p)** The trajectory was rearranged into tree format according to the defined root and terminal nodes for frontonasal (m), mandibular (n), periocular (o), and periotic (p) mesenchymal subsets. Terminal nodes are labeled according to the annotations in the panels above. **(q-t)** Trajectory (q,s) and dendrogram (r,t) for nose (q,r) and jaw (s,t) merged samples.

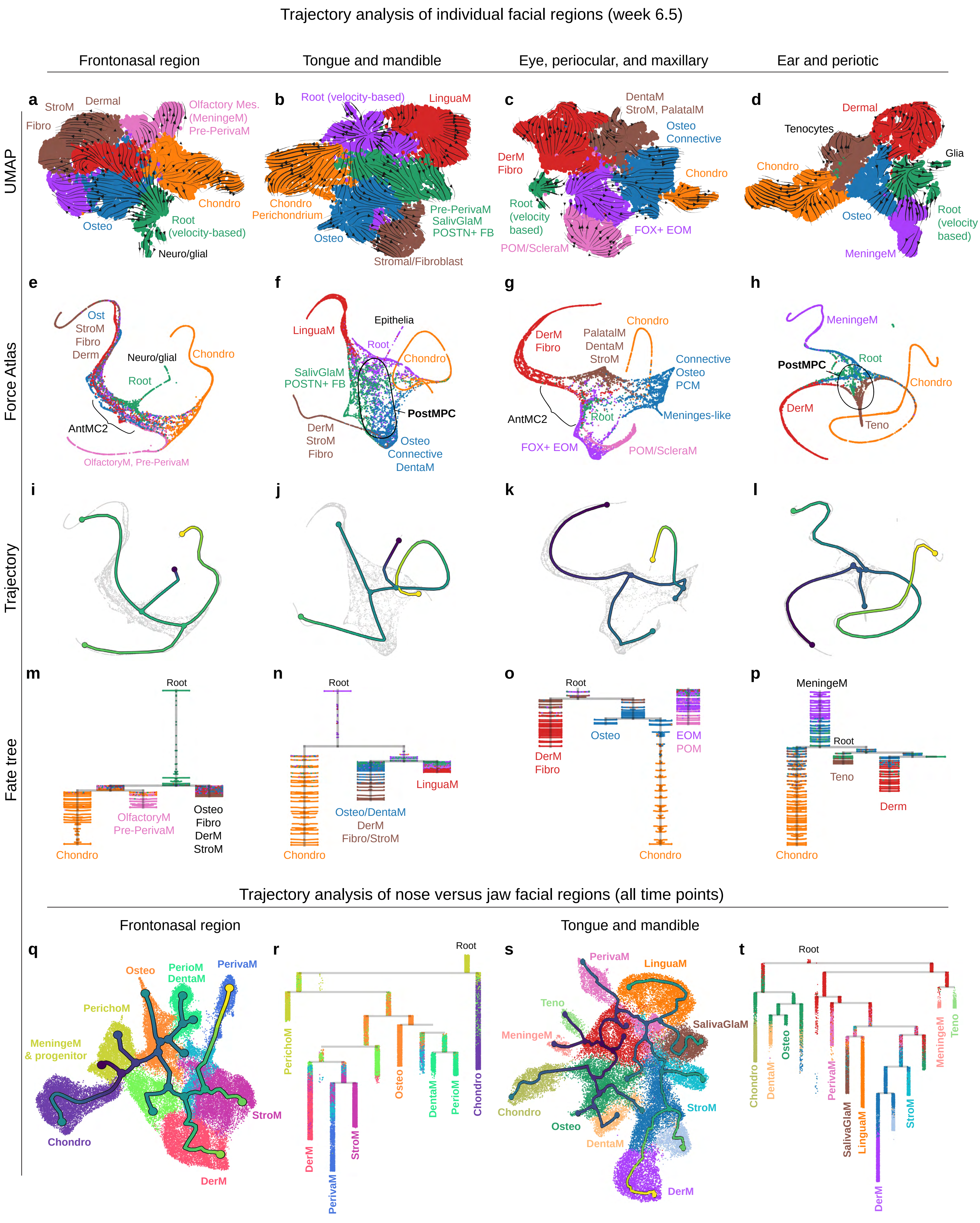

Supplementary Figure 3

Supplementary Figure 4 - *Region-specific differentially accessible regions*
Heatmap of differentially accessible regions (DARs) across cell clusters. Columns represent annotated cell clusters (coarse-grained) subdivided by facial region of origin. Values are row-scaled log_2FC, with color indicating relative accessibility (blue = decreased, red = increased).

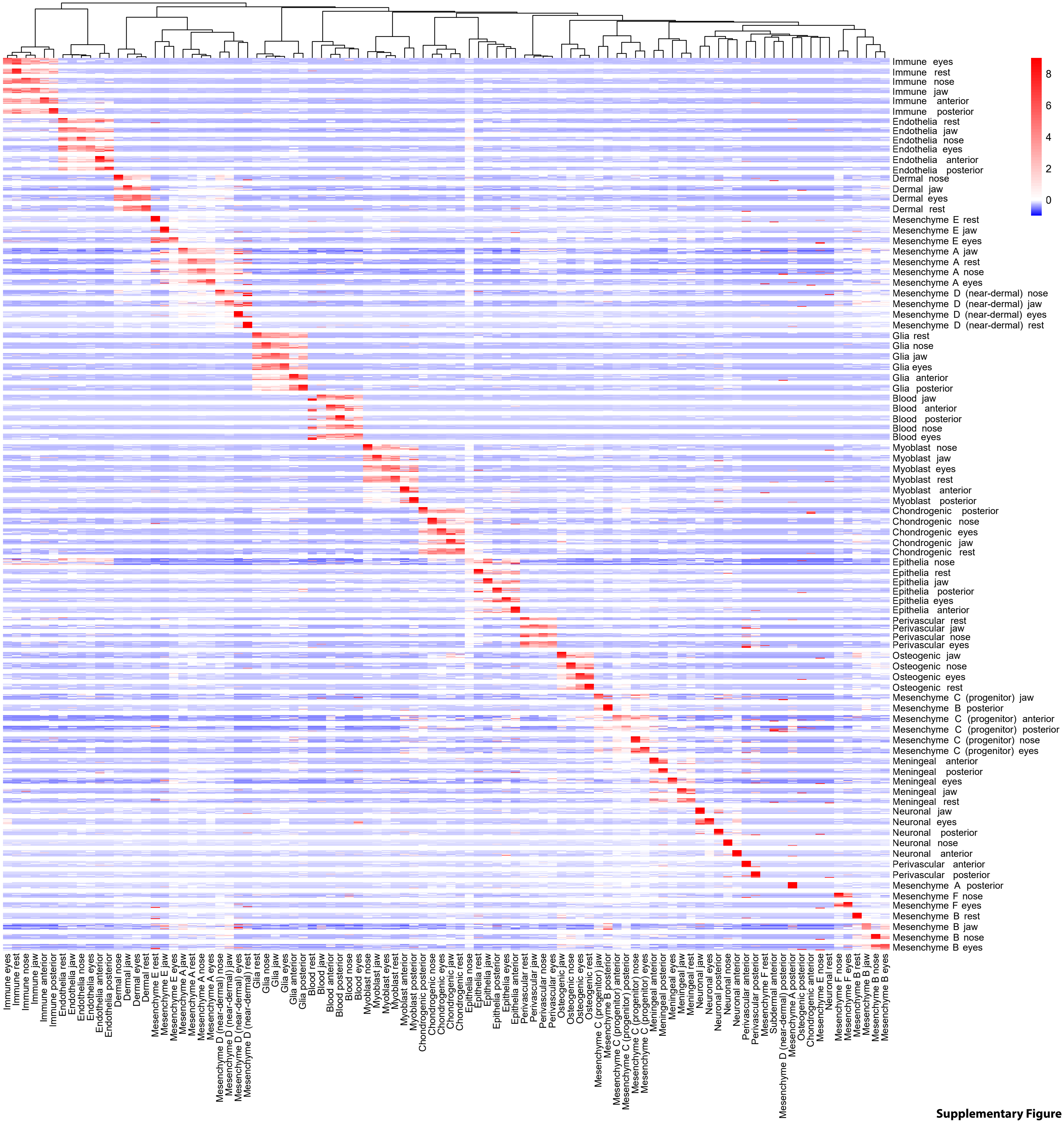

Supplementary Figure 4

Supplementary Figure 5 - *Visium analysis of tissue near sensory organs*

- **a.** Hematoxylin and Eosin stain of the periocular section. Thick black dotted line indicates the fiducial frame. POM = periocular mesenchyme.
- **b.** Leiden clusters of the periocular section.
- **c.** Periocular section is color-coded by expression of genes within 0.5 Mbp of significant (at least PV6) face-related SNPs.
- **d.** Hematoxylin and Eosin stain of the sagittal PCW11 tongue section. Thick black dotted line indicates the fiducial frame.
- **e.** Leiden clusters of the tongue section.
- **f.** Tongue section is color-coded by expression of genes within 0.5 Mbp of significant (at least PV6) face-related SNPs.
- **g.** Hematoxylin and Eosin stain of the frontal nasomaxillary section. Thick black dotted line indicates the fiducial frame.
- **h.** Leiden clusters of the nasomaxillary section.
- **i.** Nasomaxillary section is color-coded by expression of genes within 0.5 Mbp of significant (at least PV6) face-related SNPs.

Scale bars = 1 mm.

OlfactoryM = Olfactory mesenchyme. AntFB = Anterior fibroblast. DerFB = Dermal Fibroblast. Preskeletal MPC = preskeletal mesenchymal progenitor cell. Connect MC = connective tissue mesenchymal cell. PCM = perichondrium mesenchymal cell. Dermal MPC = dermal mesenchymal progenitor cell. PTN FB = periosteum-like, pleiotrophin-expressing fibroblast. AntMC2 = anterior mesenchymal cell 2. Meninges-like = strong expression of the gene expression signature associated with the meninges cell cluster. StroM, stromal mesenchymal cell. EOM, extraocular mesenchyme; Perivasc, perivascular cells

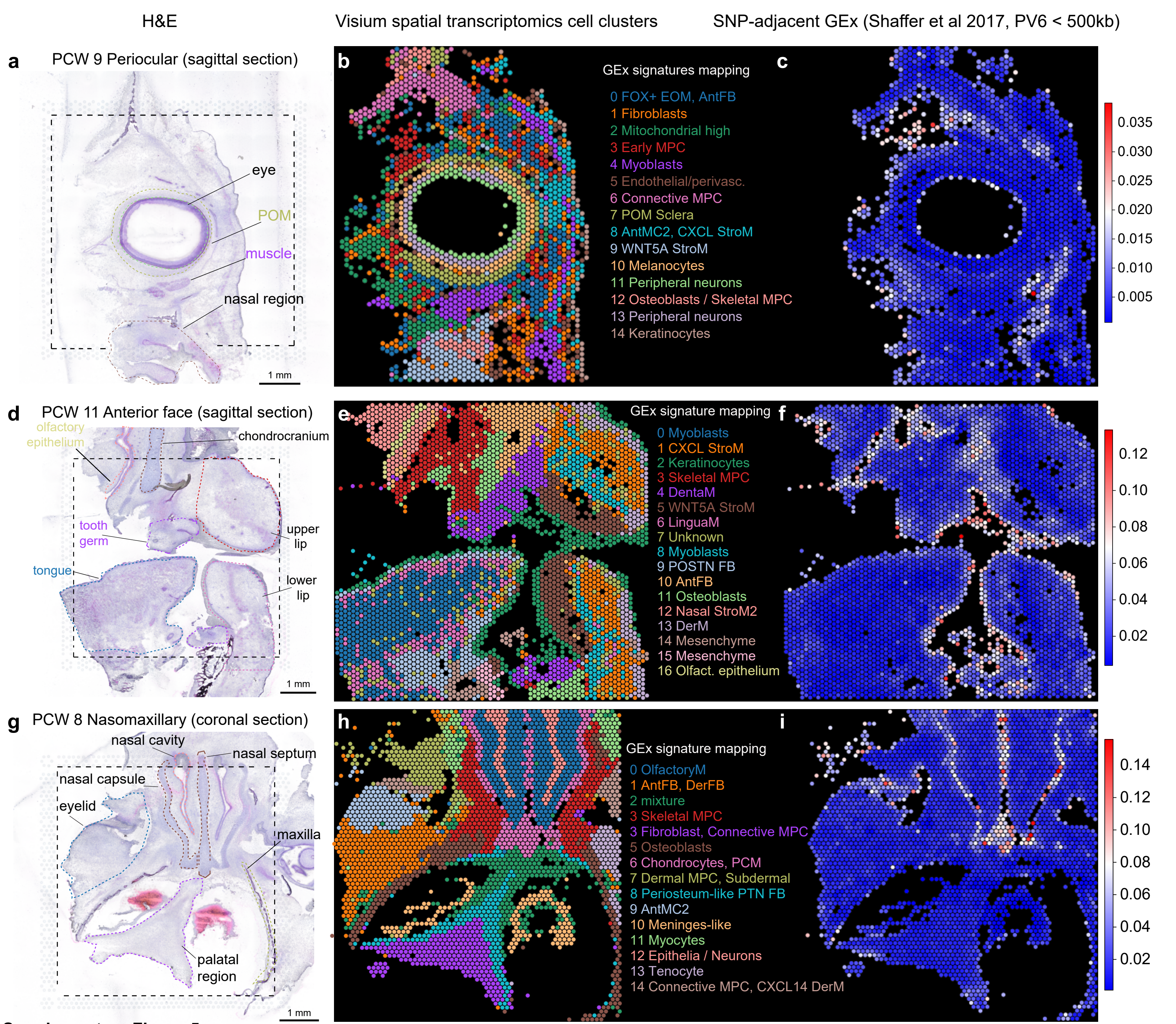

Supplementary Figure 5

Supplementary Figure 6 - *Regulon activity of spatial transcription factors*
Predicted transcriptional factor activity of spatial TFs, based on regulons within enhancer gene regulatory networks (eGRN) calculated via SCENIC plus. Each row indicates a different cell type (focusing on mesenchymal cells and their derivatives, glia included because of their transcriptional similarity with neural crest cells); each column reports specific activation (left) or repression (right) of the indicated regulon (target gene and peak ensemble). Dot size reports accessibility of peaks containing binding sites for the TF; color reports expression of genes in the regulon.

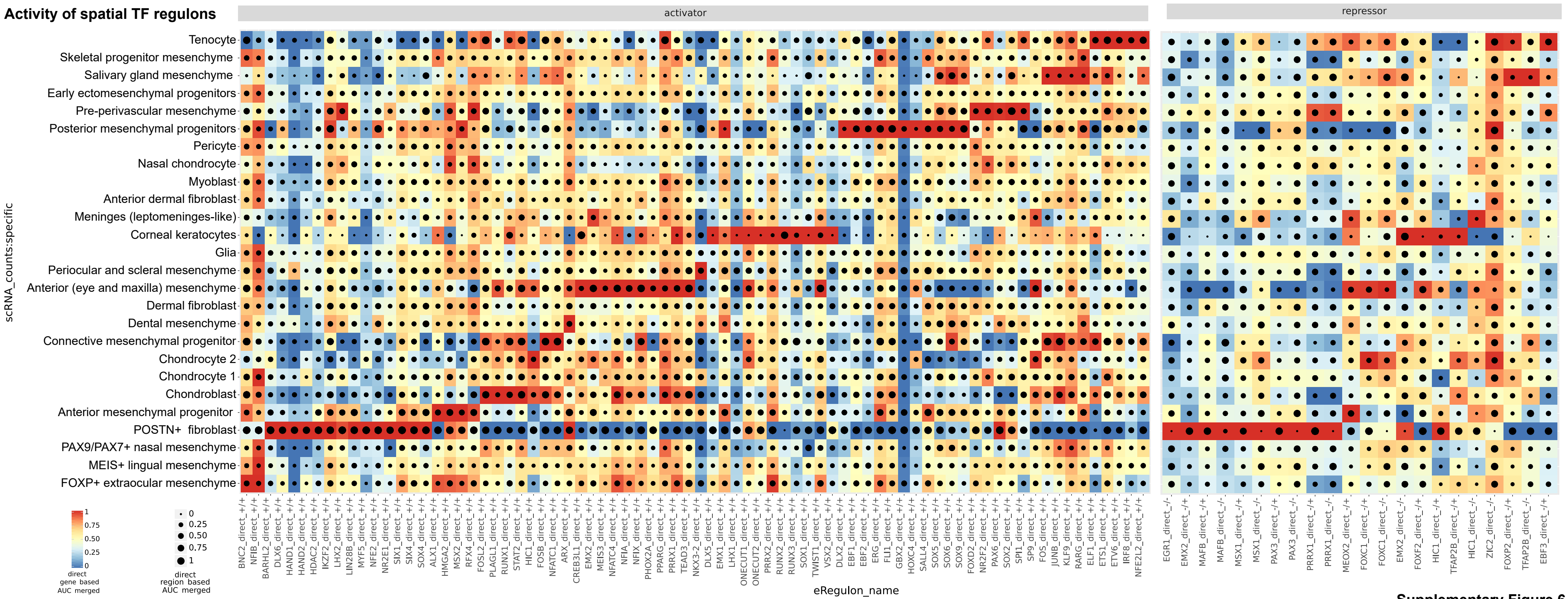

Supplementary Figure 6

Supplementary Figure 7 - *Identifying gene targets for thousands of facial enhancers*

- **a.** Histogram showing the distribution of genomic distances from each peak to the linked target gene.
- **b.** Histogram showing the distribution of average genomic distances from each gene to the set of peaks linked to that gene.
- **c.** Violin plots showing the evolutionary conservation (Average PhastCons score per peak) of linked versus all peaks, stratified by annotation; note increased score in linked versus all intergenic peaks. Across annotation classes, gene-linked peaks show higher conservation than the class-matched background (Wilcoxon rank-sum, BH-adjusted $q<10^{-5}$ for 5/6 classes; median increases 13–70%); 'Downstream' was not significant ($q=0.28$).
- **d.** Scatterplot showing each gene according to the number of linked peaks (y-axis) and the average genomic distance between that gene and its set of linked peaks (x-axis). Selected highly linked genes of interest are highlighted in red and labeled.
- **e.** Bar plot showing the quantification of enhancers identified amongst linked peaks by overlapping data with published epigenomic measurements from human craniofacial tissues. In total, over 18,000 gene-linked peaks were identified as enhancers by at least one metric.
- **f.** Genomic coordinates of entire links (gene to peak) are shown as stacked horizontal blue lines along the x-axis, shown for the first fifteen chromosomes. The y-axis indicates a length index (shorter links are shown above longer ones).

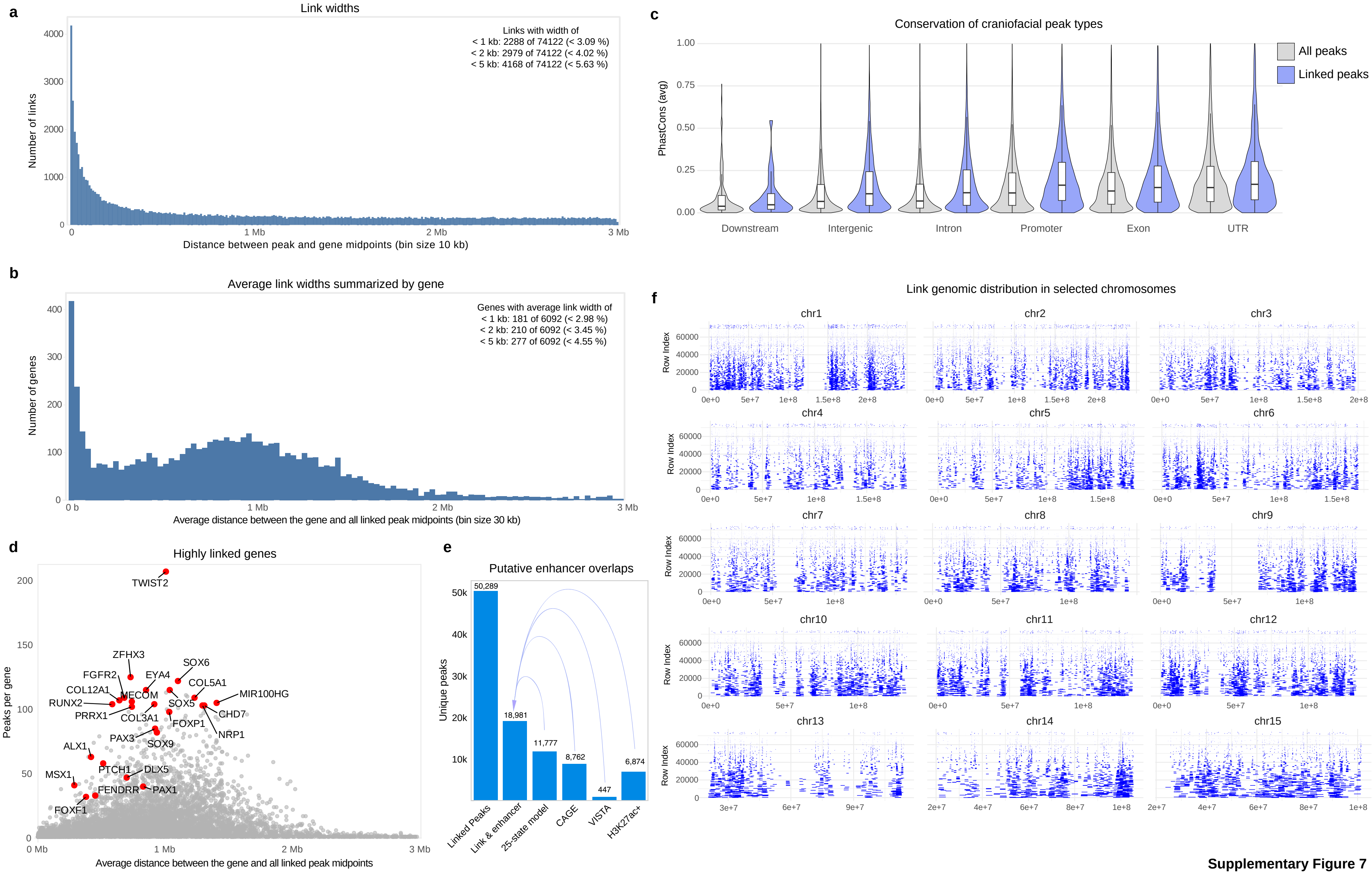

Supplementary Figure 7

Supplementary Figure 8 - *TADs do not predict the directionality of links*

- **a.** Schematic of the analysis workflow. All links were categorized based on distance between the gene and linked peak, with 200 kbp as a distance threshold.
- **b.** HiC data were clustered and piled up using coordinates from proximal links (peak in the center). Gene position relative to the linked peak is plotted below each pileup.
- **c.** Distal link loop identification.
- **d.** HiC data at selected loci, ALX1 and TFAP2B. Links are shown as arcs connecting numerous regions across multiple TADs to the gene.
- **e.** Box and whisker plots showing average PhastCons score in link types that cross (orange) or do not cross (blue) TAD boundaries, stratified by HiC classification. * = p-value < 0.05 according to MWU test with Benjamini-Hochberg correction.

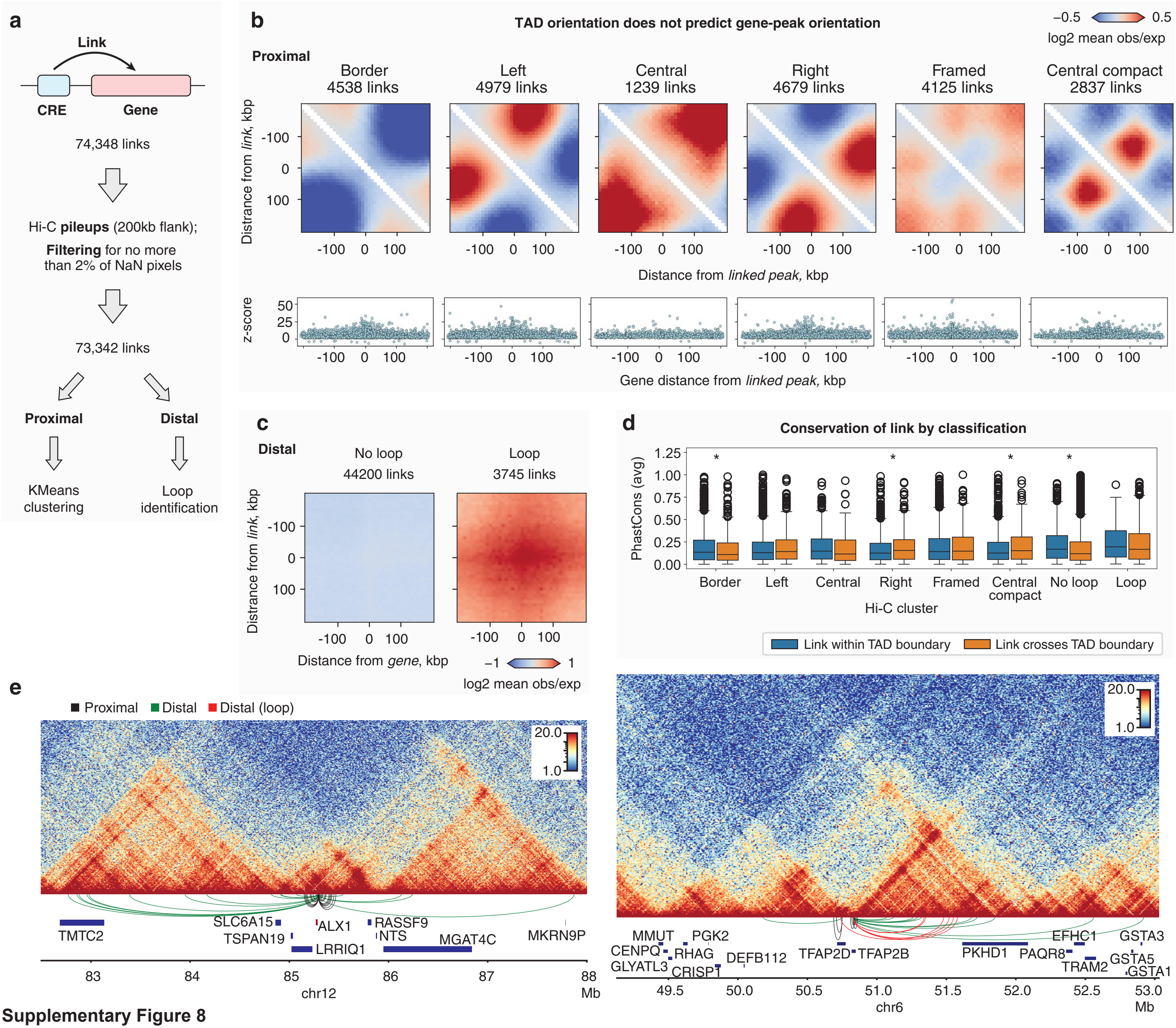

Supplementary Figure 8

Supplementary Figure 9 - *Cellular enrichment of other GWAS studies*
Heatmap showing distance-weighted enrichment z-scores (see Methods) across other facial GWAS studies and facial cell types (granular clustering). Stars indicate one-sided p-values computed from z-scores < 0.05 (nominal, not corrected for multiple comparisons).

Significant facial cell GWAS enrichment across multiple studies of variation and disease

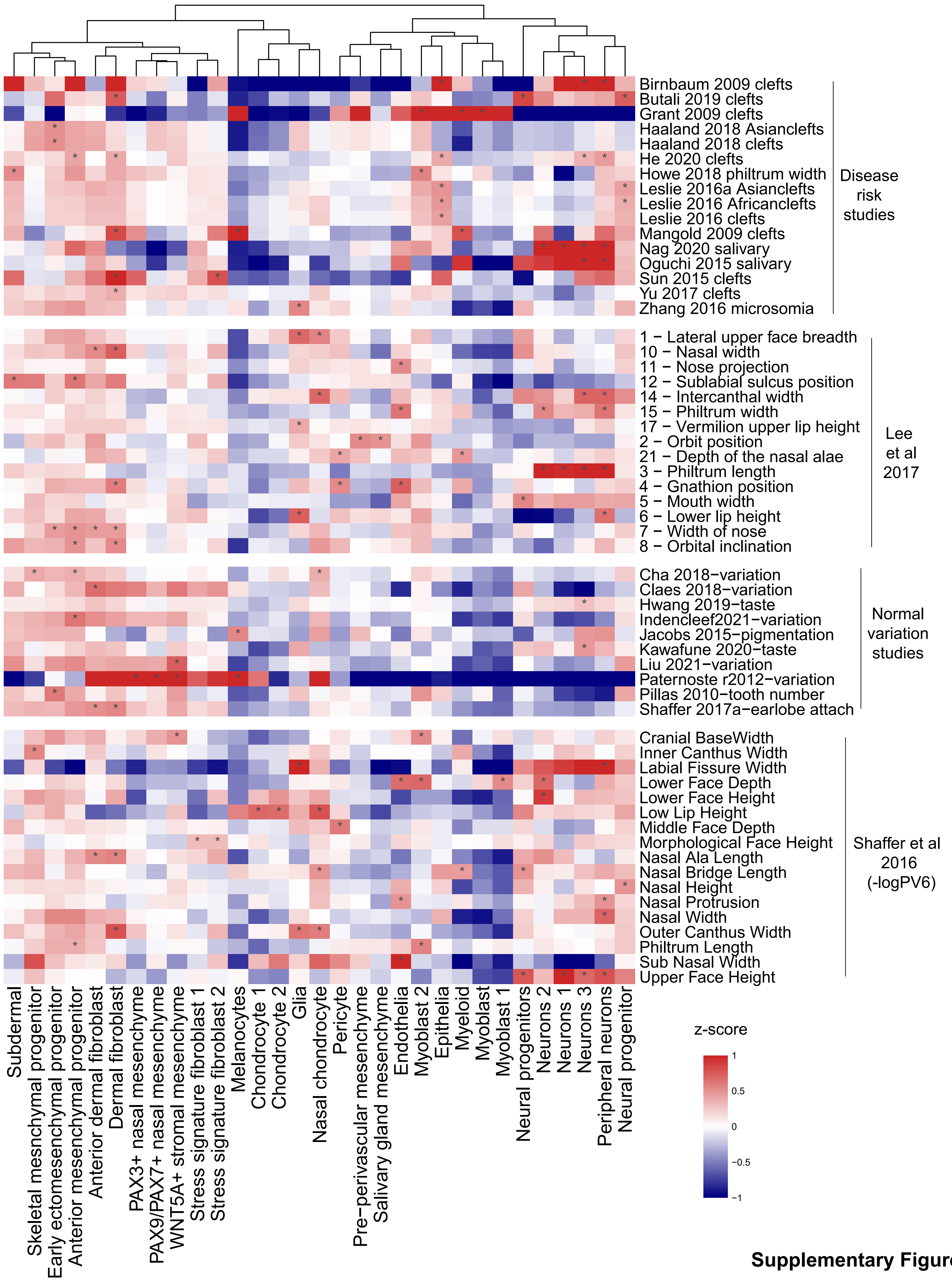

Supplementary Figure 9

Supplementary Figure 10 - *Enhancers of disease-related patterning genes may drive modern facial variation*

- **a.** Gene set enrichment across spatial, disease, and variation-linked genes within the protein coding autosomal universe.
- **b.** Gene set enrichment across spatial, disease, and variation-linked genes in the linked gene universe.
- **c.** Distribution of heterozygosity ratio across selected peak sets in different modern human populations. Dot = median, whiskers = IQR. p-values across peak sets within each population were determined via Kruskal–Wallis test.
- **d.** Box and whisker plots showing the distribution of Neanderthal introgression scores across selected peak sets in modern human populations. Dot = median, whiskers = IQR.

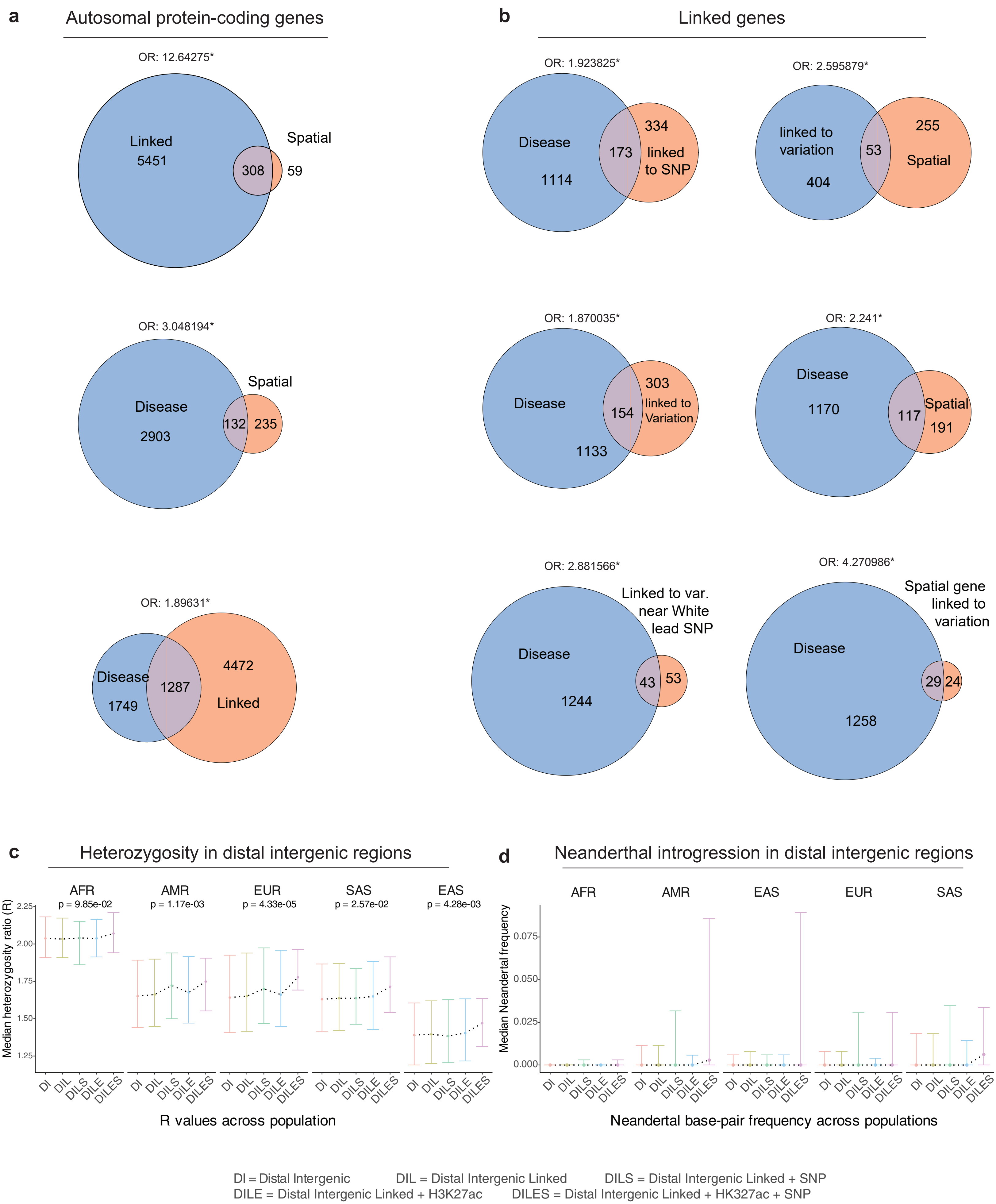

Supplementary Figure 10

Supplementary Figure 11 - *Cell type distribution of selected disease genes*
Heat map showing expression levels of selected disease genes (rows) across facial cell types (columns). Bolded genes are spatial genes that are linked to peaks containing variation SNPs.

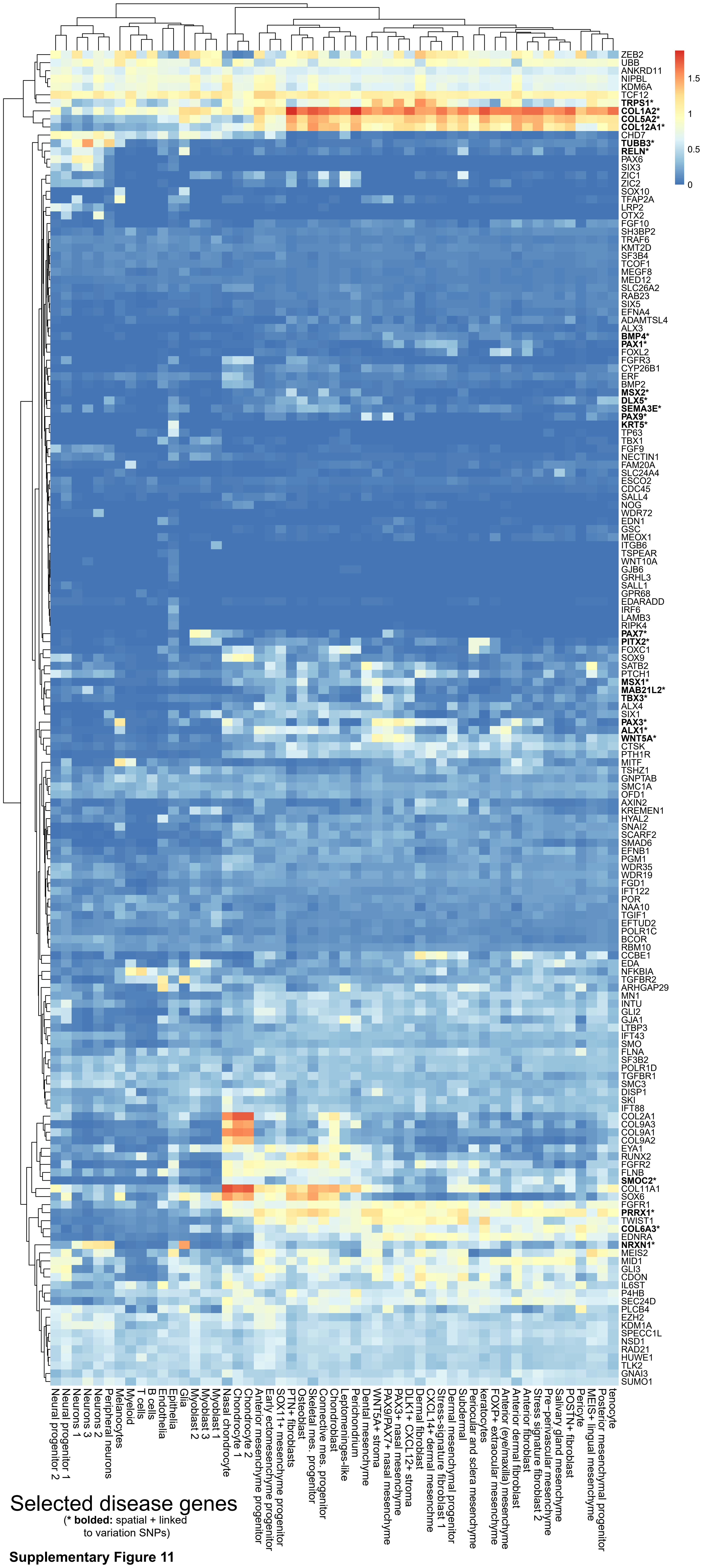

Selected disease genes

(* **bolded:** spatial + linked to variation SNPs)

Supplementary Figure 11

Supplementary Figure 12 - *Cell type distribution of imprinted genes*

Heat map showing expression levels of imprinted genes (rows) across facial cell types (columns). Bolded genes are within 500kb of variation SNPs; of those, green-colored genes are linked to peaks containing at least one variation SNP.

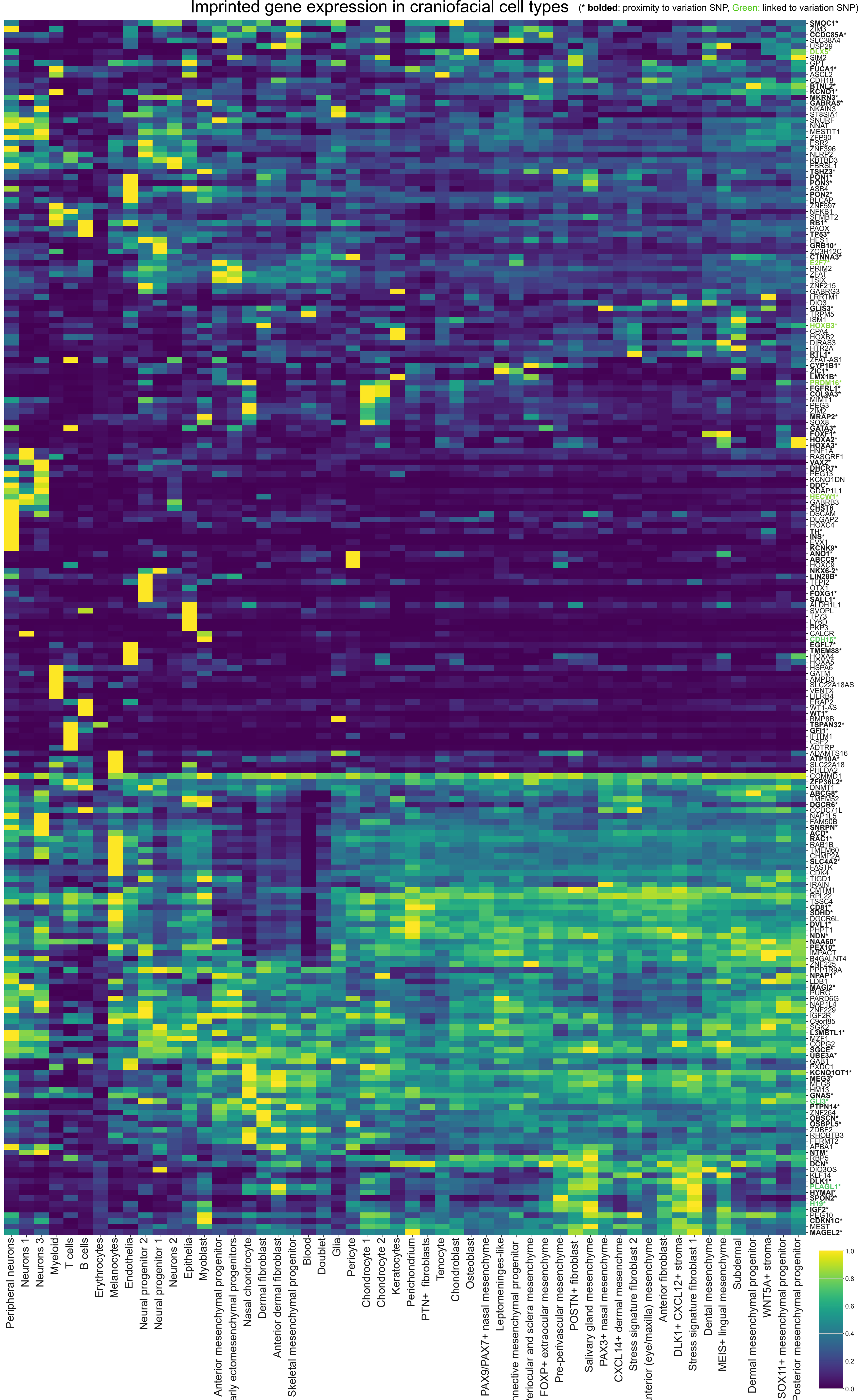

Supplementary Figure 12

Supplementary Figure 13 - *Cell type distribution of spatial signaling and spatial TF genes*
Heat map showing expression levels of genes (rows) across facial cell types (columns). Expression levels are scaled across row.

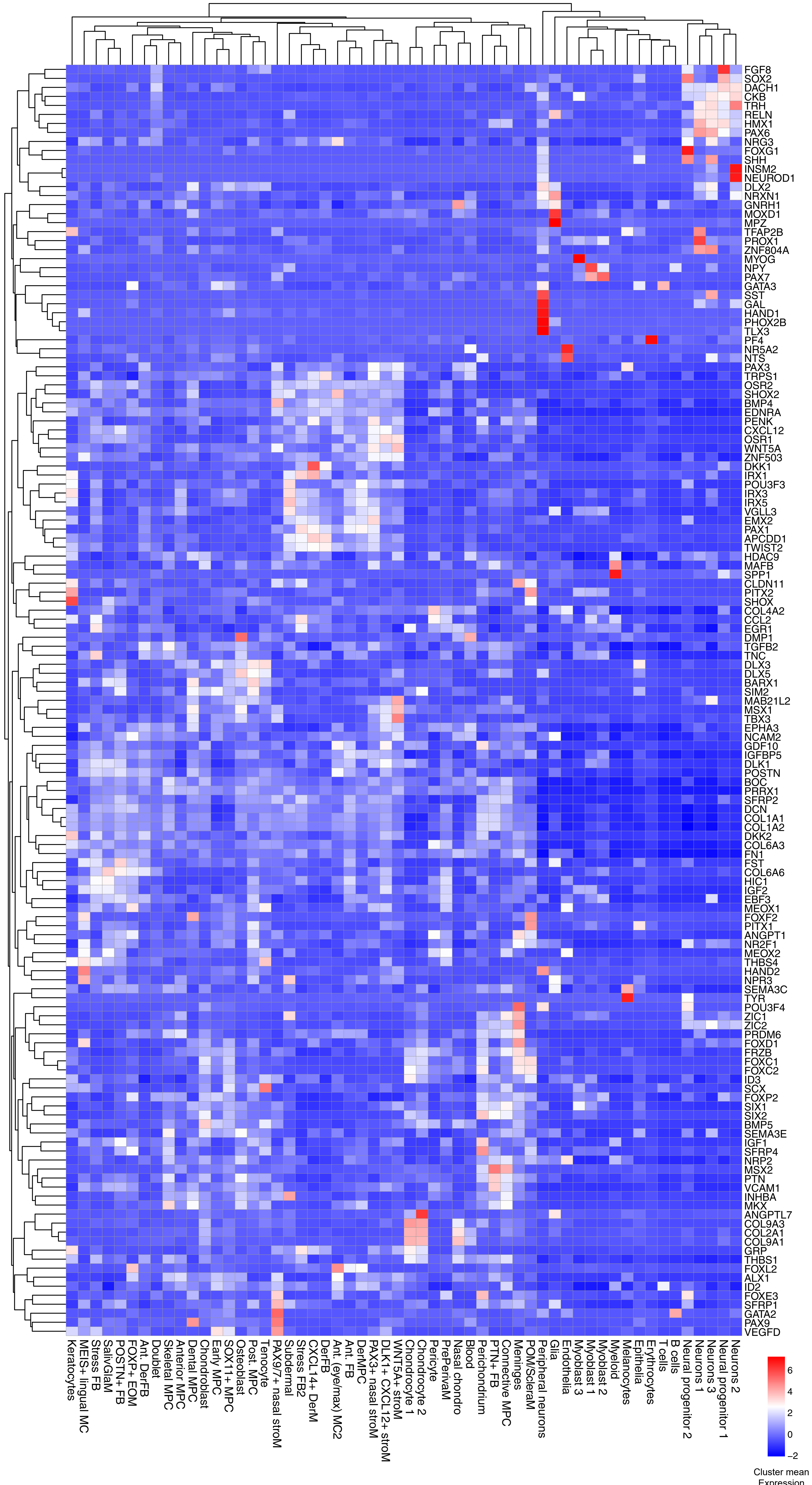

Supplementary Figure 13

Supplementary Figure 14 - *Signaling landscape of human facial tissues.*
COMMOT signaling activity maps for the top predicted pathways per each tissue section. Arrows indicate the directionality and magnitude of signaling scores. MK, midkine; PTN; pleiotrophin; IGF, insulin-like growth factor; ncWNT, non-canonical Wnt; SEMA3, Class 3 Semaphorins; CXCL, chemokine ligand; SPP1, osteopontin; GAS, growth-arrest-specific protein; PERIOSTIN, periostin.

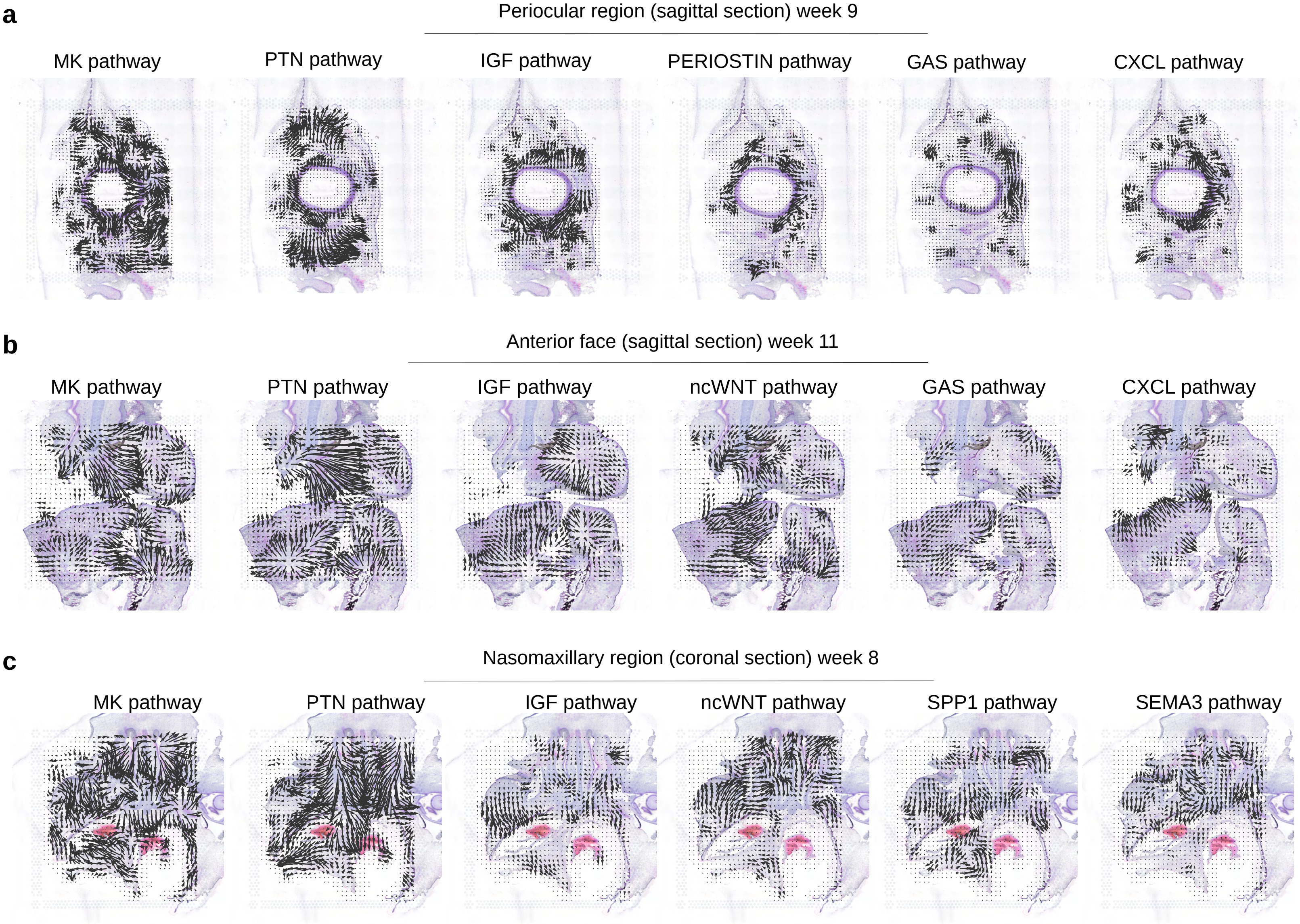

Supplementary Figure 14

Supplementary Figure 15 - *Top predicted signaling pathways in human facial tissues at PCW6.*

- **a.** COMMOT signaling activity maps for the pathways with the top predicted signaling scores across Stereoseq sections. Arrows indicate the directionality and magnitude of signaling scores. MK, midkine; PTN; pleiotrophin; IGF, insulin-like growth factor; ncWNT, non-canonical Wnt; SEMA3, Class 3 Semaphorins; NRG, neuregulin; PDGF, platelet-derived growth factor.
- **b.** Dot plot showing strength of correlation and significance of predicted target genes.

a

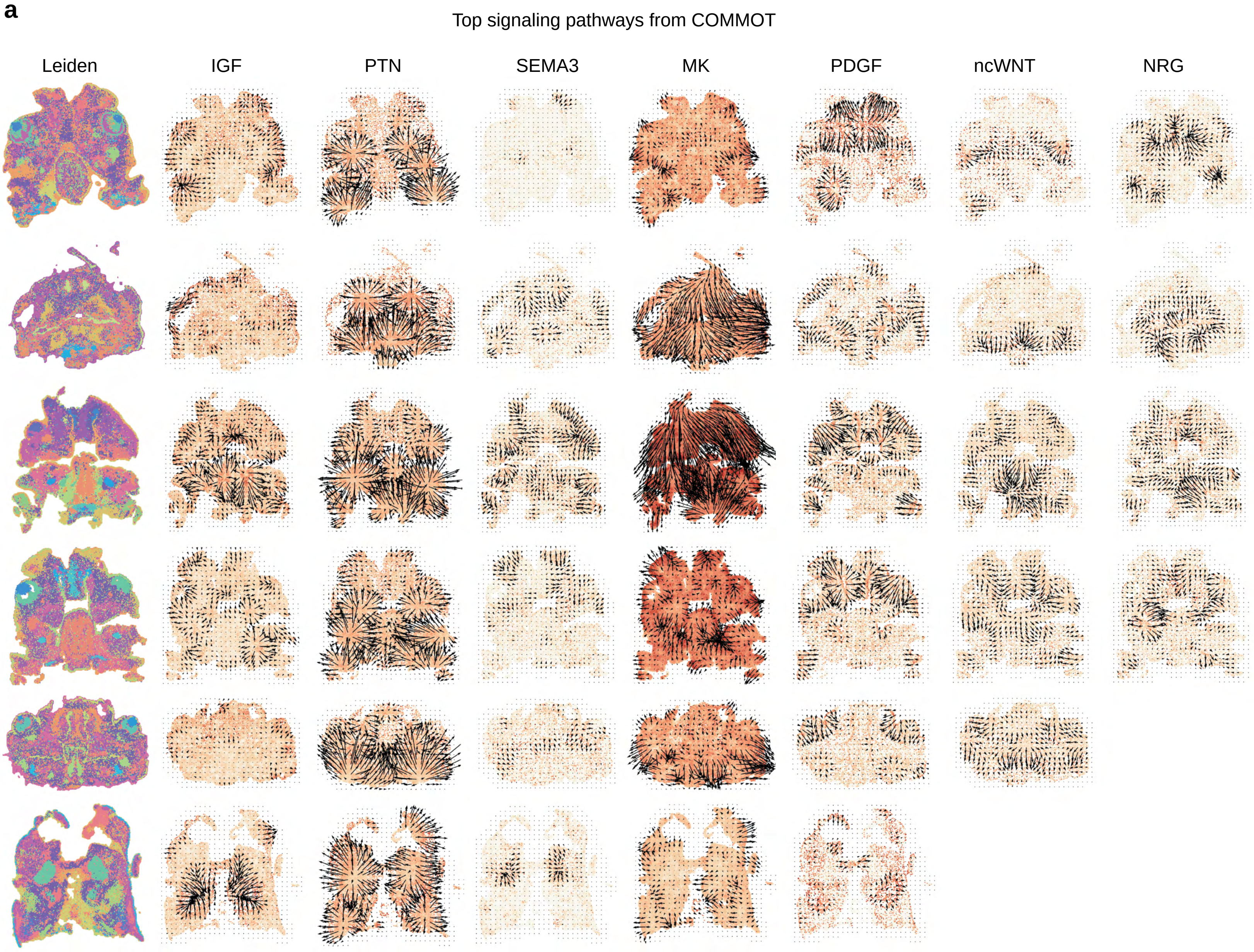

b

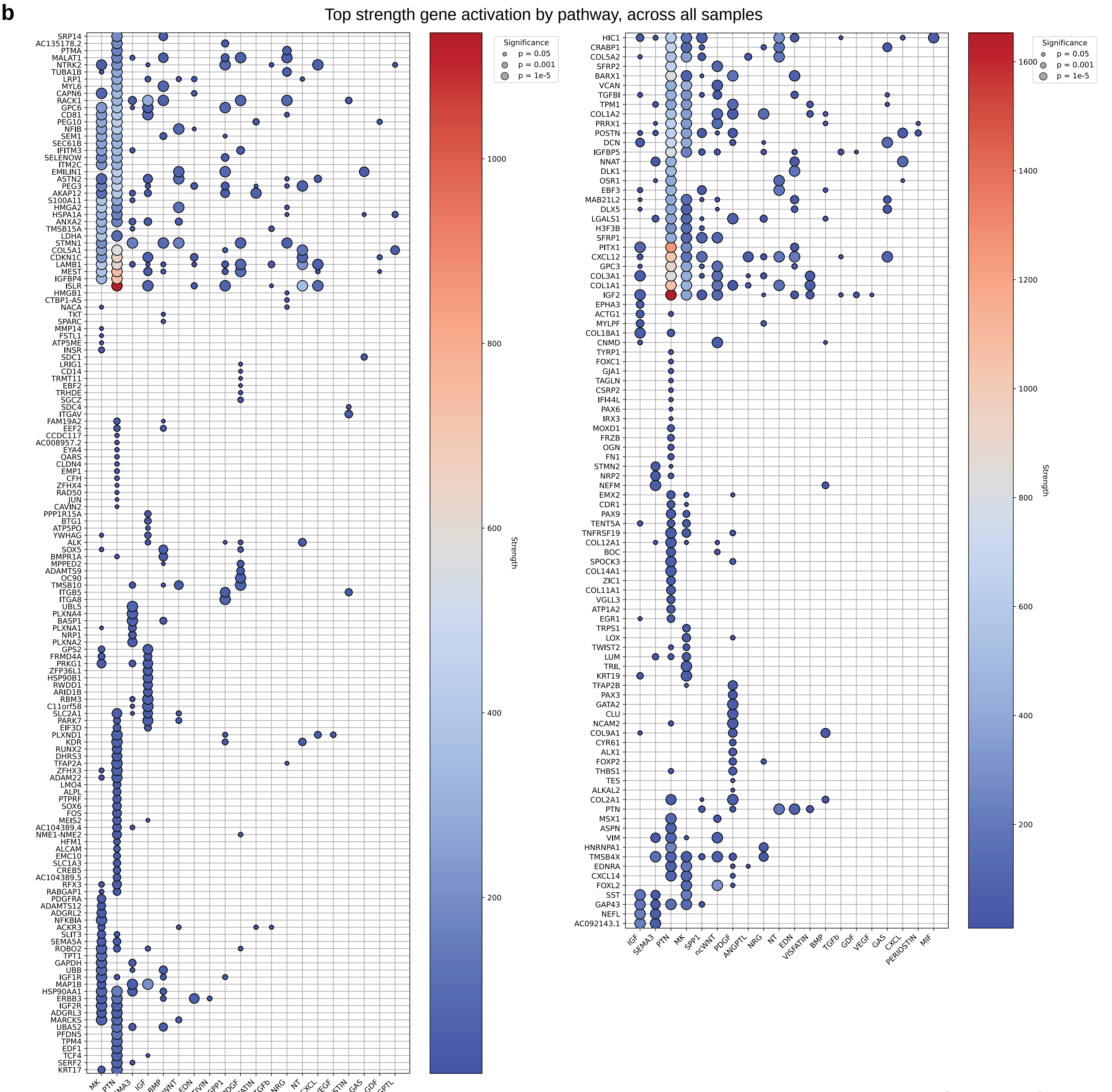

Supplementary Figure 15
