# Supplementary material for: Atlas of cell types and regulatory elements underlying human facial diversity
Source: Nat Genet. 2026 Sep 24;58(10):2645–59. doi: 10.1038/s41588-026-02748-y (PMC13649600; doi:10.1038/s41588-026-02748-y)
Supplement: Supplementary file 2 [file 41588_2026_2748_MOESM2_ESM.pdf]

nature portfolio

Corresponding author(s): Igor Adameyko
Alek Erickson

Last updated by author(s): Apr 7, 2026

Reporting Summary

Nature Portfolio wishes to improve the reproducibility of the work that we publish. This form provides structure for consistency and transparency in reporting. For further information on Nature Portfolio policies, see our Editorial Policies and the Editorial Policy Checklist.

Statistics

For all statistical analyses, confirm that the following items are present in the figure legend, table legend, main text, or Methods section.

n/a	Confirmed	
☐	☒	The exact sample size (*n*) for each experimental group/condition, given as a discrete number and unit of measurement
☐	☒	A statement on whether measurements were taken from distinct samples or whether the same sample was measured repeatedly
☐	☒	The statistical test(s) used AND whether they are one- or two-sided<br>*Only common tests should be described solely by name; describe more complex techniques in the Methods section.*
☐	☒	A description of all covariates tested
☐	☒	A description of any assumptions or corrections, such as tests of normality and adjustment for multiple comparisons
☐	☒	A full description of the statistical parameters including central tendency (e.g. means) or other basic estimates (e.g. regression coefficient) AND variation (e.g. standard deviation) or associated estimates of uncertainty (e.g. confidence intervals)
☐	☒	For null hypothesis testing, the test statistic (e.g. *F*, *t*, *r*) with confidence intervals, effect sizes, degrees of freedom and *P* value noted<br>*Give P values as exact values whenever suitable.*
☒	☐	For Bayesian analysis, information on the choice of priors and Markov chain Monte Carlo settings
☒	☐	For hierarchical and complex designs, identification of the appropriate level for tests and full reporting of outcomes
☐	☒	Estimates of effect sizes (e.g. Cohen's *d*, Pearson's *r*), indicating how they were calculated

Our web collection on statistics for biologists contains articles on many of the points above.

Software and code

Policy information about availability of computer code

Data collection	micro-CT analysis of human embryos was executed using a GE phoenix v|tome|x L 240 system, equipped with a nanofocus X-ray tube and a high-contrast flat panel detector as described21. The acceleration voltage of X-ray tube was 80 kV with 200 µA current. Exposure time was 500 ms and three projections were acquired and averaged in each position (2100-2300 projections depending on the sample width) over 360 degrees. The voxel size of the generated volumes varied depending on the embryo size in the range between 11 and 20 µm, and tomographic reconstructions were performed with specialized GE phoenix datos|x software. For the cartilage segmentation, Avizo software was used, and the data was transformed into a polygonal mesh. Surface smoothing of this mesh was done using VG Studio MAX 2.2 software<br><br>CT scans of mice were performed using the Mediso NanoScan PET/CT system, operated via Nucline software. The acquisition parameters were as follows: 720 projections per rotation, helical scanning mode with a pitch of 1, Ultra Zoom setting, exposure time of 170 ms, binning ratio of 1:4, and an X-ray tube voltage of 35 kVp. Image reconstruction was conducted using Fourier back-projection within the same software environment. The resulting volumetric dataset consists of isotropic voxels with an edge length of 49 µm. Downstream image analysis was performed at the Central European Institute for Technology (CEITEC) using Avizo software and VG Studio MAX 2.2 software as above.
Data analysis	Code Availability Statement<br>Original code has been deposited to GitHub and can be found using the following link:<br>https://github.com/TBouderlique/human_face_development<br><br>The MeshMonk (v0.0.6) spatially dense facial mapping software is free to use for academic purposes (https://github.com/TheWebMonks/meshmonk).

For manuscripts utilizing custom algorithms or software that are central to the research but not yet described in published literature, software must be made available to editors and reviewers. We strongly encourage code deposition in a community repository (e.g. GitHub). See the Nature Portfolio guidelines for submitting code & software for further information.

Data

Policy information about availability of data

All manuscripts must include a data availability statement. This statement should provide the following information, where applicable:

- Accession codes, unique identifiers, or web links for publicly available datasets
- A description of any restrictions on data availability
- For clinical datasets or third party data, please ensure that the statement adheres to our policy

Data Availability Statement
Explore the dataset on CELLXGENE:
https://adameykolab.hifo.meduniwien.ac.at/cellxgene_public/
CAGE data has been made available in the Supp. Table 7. The raw CAGE data has also been analyzed in a different context by our team as part of a different published paper (accessible here: https://zenodo.org/records/10363659).
MicroCT scans have been submitted to Zenodo and will be available under the following DOI https://doi.org/10.5281/zenodo.17347142
Count matrix data has been submitted to Dryad, and is accessible under the following DOI:
https://doi.org/10.5061/dryad.7m0cfxq8t
FASTQ files have been deposited to the Karolinska Institute Data Repository via the DORIS system from the Swedish National Data Service (SND): https://doi.org/10.48723/pq77-v833b

Research involving human participants, their data, or biological material

Policy information about studies with human participants or human data. See also policy information about sex, gender (identity/presentation), and sexual orientation and race, ethnicity and racism.

Reporting on sex and gender	not applicable
Reporting on race, ethnicity, or other socially relevant groupings	not applicable
Population characteristics	The tissue donors were recruited among pregnant women after their decision to terminate their pregnancy. All patients were 18 years of age or older and fluent in Swedish.<br><br>Exclusion criteria: abortions performed for any medical reasons, by socially compromised women and/or by women showing any signs that the consent may not be informed.<br><br>All women provided written consent for tissue usage for research purposes and for their ability to withdraw their consent at any time.
Recruitment	Human prenatal tissue was obtained from elective terminations of pregnancy (induced abortions) with informed oral and written consent from the patient, and pseudonymized. The referral to hospitals was done by a central office for all abortion clinics in the Stockholm region. The recruitment was handled by midwives who were not involved in the research study.
Ethics oversight	Ethical approval<br>All procedures regarding the acquisition of human pre-natal tissue for research was approved by the Swedish Ethical Research Authority and the National Board of Health and Welfare (ethical references are 2018/769-31, 2019-04595, 2020-03045 and 2024/00280-02). Use of fixed embryos for micro-CT analysis was approved by the Ethics board of the Masaryk University (ethical reference is 79/2022).

Note that full information on the approval of the study protocol must also be provided in the manuscript.

Field-specific reporting

Please select the one below that is the best fit for your research. If you are not sure, read the appropriate sections before making your selection.

☒ Life sciences ☐ Behavioural & social sciences ☐ Ecological, evolutionary & environmental sciences

For a reference copy of the document with all sections, see nature.com/documents/nr-reporting-summary-flat.pdf

Life sciences study design

All studies must disclose on these points even when the disclosure is negative.

Sample size	Sample size was not determined ahead of the study. We aimed to cover multiple critical stages, with 2-3 embryos per time point for scRNAseq (except PCW10.5, n=1 embryo for scRNAseq). Twenty embryos were analyzed via transcriptomics-based methods in this study. Five embryos were used for Micro-CT in the study (at 8-12 weeks of gestation). For scanning of mouse bones during functional experiments for the role of

peripheral nerves (n=5 E18.5 Neurog1-KO mouse embryos, n=11 littermate heterozygote/WT mouse embryos, from n=3 litters) and the role of the predicted PAX1 enhancer (n=6 'small' mice from the PAX1-cCRE line and n=5 littermates, from n=2 litters), mice were sacrificed and subsequently analyzed with similar results across experiments.

Data exclusions	One of the microCT scans of a human embryo showed significant deformation of the embryo, and this scan was excluded.
Replication	Cell types observed across biological replicates are very similar.<br>Functional tests in mice showed consistent effects results across multiple independent embryos and across independent litters.
Randomization	Not applicable. The study is primarily descriptive/atlas study. For the few experiments that were performed, allocation of mice to experimental groups was done according to genotype or an obvious phenotype.
Blinding	Blinding was not possible. This study is primarily a descriptive atlas study, so no group allocation is possible. In case of experiments, blinding would be impossible due to the obvious phenotypic differences between the groups studied.

Reporting for specific materials, systems and methods

We require information from authors about some types of materials, experimental systems and methods used in many studies. Here, indicate whether each material, system or method listed is relevant to your study. If you are not sure if a list item applies to your research, read the appropriate section before selecting a response.

Materials & experimental systems

n/a	Involved in the study
☒	☐ Antibodies
☒	☐ Eukaryotic cell lines
☒	☐ Palaeontology and archaeology
☐	☒ Animals and other organisms
☒	☐ Clinical data
☒	☐ Dual use research of concern
☒	☐ Plants

Methods

n/a	Involved in the study
☒	☐ ChIP-seq
☒	☐ Flow cytometry
☒	☐ MRI-based neuroimaging

Animals and other research organisms

Policy information about studies involving animals; ARRIVE guidelines recommended for reporting animal research, and Sex and Gender in Research

Laboratory animals	Mus musculus<br><br>Animals from non-genetically modified mouse lines CD-1 and C57BL/6 were purchased from Charles River Laboratories and Janvier. Neurogenin 1 knockout (Neurog1Tm2and) mice were purchased from Jax Lab (RRID: IMSR_JAX:017306).<br><br>E18.5 Neurog1-KO mouse embryos<br>Pax1-cCRE: 2 months of age
Wild animals	not applicable
Reporting on sex	no specific sex studies or sex selection was done for embryological work reported in this manuscript.
Field-collected samples	not applicable
Ethics oversight	All animal experiments were approved by the Swedish Animal Welfare Body Jordbruksverket De regionala Djurförsöksetiska nämnderna, Stockholms Djurförsöksetiska nämnd, and Göteborgs djurförsöksetiska nämnd, permits N15907-2019, 18314-2021, and 004225.

Note that full information on the approval of the study protocol must also be provided in the manuscript.

Plants

Seed stocks

not applicable

Novel plant genotypes

Describe the methods by which all novel plant genotypes were produced. This includes those generated by transgenic approaches, gene editing, chemical/radiation-based mutagenesis and hybridization. For transgenic lines, describe the transformation method, the number of independent lines analyzed and the generation upon which experiments were performed. For gene-edited lines, describe the editor used, the endogenous sequence targeted for editing, the targeting guide RNA sequence (if applicable) and how the editor was applied.

Authentication

Describe any authentication procedures for each seed stock used or novel genotype generated. Describe any experiments used to assess the effect of a mutation and, where applicable, how potential secondary effects (e.g. second site T-DNA insertions, mosiacism, off-target gene editing) were examined.
