# Supplementary material for: Atlas of cell types and regulatory elements underlying human facial diversity
Source: Nat Genet. 2026 Sep 24;58(10):2645–59. doi: 10.1038/s41588-026-02748-y (PMC13649600; doi:10.1038/s41588-026-02748-y)
Supplement: Supplementary file 3 [file 41588_2026_2748_MOESM3_ESM.pdf]

nature portfolio

Peer Review File

Cell types, positional codes, and enhancers contributing to human facial individuality and pathology

Corresponding Author: Professor Igor Adameyko

Version 0:

Decision Letter:

8th Apr 2024

Dear Igor,

Firstly, thank you to your and your co-authors for your patience during this prolonged review period.

Your Article, "Characterization of cell types, positional codes, and enhancers contributing to human facial individuality and pathology" has now been seen by 3 referees. You will see from their comments below that while they find your work of interest, some important points are raised. We are interested in the possibility of publishing your study in Nature Genetics, but would like to consider your response to these concerns in the form of a revised manuscript before we make a final decision on publication.

In sum, the reviewers all acknowledge the value of your dataset and say that your work is potentially suitable for publication, but suggest further improvement is required.

Referee #1 - as fitting their background - provides a brief report focused on the potential clinical application of your data, suggesting it is not clear what the exact utility of your data is for furthering our understanding of craniofacial disorders.
Reviewer #2 sums up their opinion as a recommendation to "significantly reorganize the manuscript and figures such that there is first a description of all the data collected, and then a handful of more in-depth vignettes illustrating the utility of the data by analyzing this rich resource in light of known craniofacial biology"; they provide some further specific comments that we think are also important (e.g. on using a more up-to-date GWAS).
Reviewer #3 sounds positive but also makes a number of useful observations and recommendations, e.g. noting that the n_sample is small.

In our reading of these reviews there is clear guidance provided that will address many of those concerns. Most notably, we think that Referee #1's comments on the demonstration of (clinical) utility of your data are very much of a piece with the other reviews: per Referee #2, these presentational aspects need major strengthening, such that the novelty of your work is clearer. We note there are also specific technical comments on e.g. the lack of significant overlap between your genesets and published ones that will also require attention.

To guide the scope of the revisions, the editors discuss the referee reports in detail within the team, including with the chief editor, with a view to identifying key priorities that should be addressed in revision and sometimes overruling referee requests that are deemed beyond the scope of the current study. We hope that you will find the prioritized set of referee points to be useful when revising your study. Please do not hesitate to get in touch if you would like to discuss these issues further.

We therefore invite you to revise your manuscript taking into account all reviewer and editor comments. Please highlight all changes in the manuscript text file. At this stage we will need you to upload a copy of the manuscript in MS Word .docx or similar editable format.

We are committed to providing a fair and constructive peer-review process. Do not hesitate to contact us if there are specific requests from the reviewers that you believe are technically impossible or unlikely to yield a meaningful outcome.

When revising your manuscript:

*1) Include a “Response to referees” document detailing, point-by-point, how you addressed each referee comment. If no

action was taken to address a point, you must provide a compelling argument. This response will be sent back to the referees along with the revised manuscript.

*2) If you have not done so already please begin to revise your manuscript so that it conforms to our Article format instructions, available
here.
Refer also to any guidelines provided in this letter.

*3) Include a revised version of any required Reporting Summary: https://www.nature.com/documents/nr-reporting-summary.pdf
It will be available to referees (and, potentially, statisticians) to aid in their evaluation if the manuscript goes back for peer review.
A revised checklist is essential for re-review of the paper.

Please be aware of our guidelines on digital image standards.

Please use the link below to submit your revised manuscript and related files:

Link Redacted

Note: This URL links to your confidential home page and associated information about manuscripts you may have submitted, or that you are reviewing for us. If you wish to forward this email to co-authors, please delete the link to your homepage.

Please do not hesitate to contact me if you have any questions or would like to discuss these revisions further.

Nature Genetics is committed to improving transparency in authorship. As part of our efforts in this direction, we are now requesting that all authors identified as ‘corresponding author’ on published papers create and link their Open Researcher and Contributor Identifier (ORCID) with their account on the Manuscript Tracking System (MTS), prior to acceptance. ORCID helps the scientific community achieve unambiguous attribution of all scholarly contributions. You can create and link your ORCID from the home page of the MTS by clicking on ‘Modify my Springer Nature account’. For more information please visit please visit www.springernature.com/orcid.

We look forward to seeing the revised manuscript and thank you for the opportunity to review your work.

Sincerely,

Michael Fletcher, PhD
Senior Editor, Nature Genetics
ORCiD: 0000-0003-1589-7087

Referee expertise:

Referee #1: bioinformatics; craniofacial disorders.

Referee #2: craniofacial genetics; human genetics; single-cell genomics

Referee #3: tissue and organ development, including craniofacial

Reviewers' Comments:

Reviewer #1:
Remarks to the Author:
Erickson et al. present spatial transcriptomics data at single-cell resolution for human facial microdisections at different developmental stages. This data could become an essential resource for everyone interested in the gene regulatory mechanism involved in craniofacial dysmorphism. The spatial scRNA-seq data was generated with Visium and StereoSeq, while cell types and clusters were identified by algorithms such as Tangram, Leiden, and Harmony.

So far, the genetic architecture of the human face has been analyzed by genome wide association studies, as well as in medical genetics. While GWAS results point to loci in the genome that are involved in the normal variation of the face, pathogenic variants causing Mendelian disorders with facial dysmorphism are of higher effect size.

Major
The authors computed gene-peak pairs with correlated patterns across cell populations. I tried to run a few analyses myself for the gene sets identified by the authors and those from references 2,4,7,42,53,54 (mainly 2). I did not find any significant overlaps. Since this is not necessarily to be expected, this is not a criticism of their results. However, could you please

comment on why we see so little overlap between the "links" from spatial transcriptomics and GWAS?

I also checked facial phenotypes in OMIM and the GestaltMatcher database for all the genes discussed by the authors and found a slightly higher hit rate for facial dysmorphism. Besides PAX1, I found relevant medical conditions for CCDC8, TGFBR2, TBX3, LTBP4, and ALX4. However, I am not sure, whether I am able to make any sense of the abnormal medical phenotype and the spatial scRNA data. I was looking for the genes in the signatures of the Leiden clusters and the spatial information and expected to find genes linked to a facial abnormality in close proximity. Could you comment on how you think the atlas can be used in medical genetics?

Minor
In line 496f you state that grandchildren share a stronger resemblance with their grandparents. I doubt it. Do you have a reference?

Reviewer #2:
Remarks to the Author:
This paper describes a multimodal atlas comprising scRNA-seq, multimodal scRNA + ATAC-seq, spatial transcriptomics, and microCT of the developing human face. The stated goal of the paper is to identify the specific cell types and spatiotemporal gene expression patterns that give rise to both normal-range and disease-associated human craniofacial variation. To do this, the authors map the expression of genes and candidate enhancers near SNPs associated with facial variation, predict cell-cell signaling interactions, and identify positional variation in gene expression and chromatin accessibility in mesenchymal and cartilage cells. They conclude that this atlas has and will provide significant utility in mechanistically understanding how the human face is formed.

Overall, I believe that the data described in this paper will be a significant resource for understanding human craniofacial development and its variation at a more granular and mechanistic level than has previously been possible. This type of in-depth molecular characterization of early human craniofacial development does not exist elsewhere. It seems that the quality of the data themselves are reasonably high, and that the rest of the field should be able to use them in interesting and impactful ways. However, the presentation of the data and associated narrative in its current form do not demonstrate this utility in a coherent fashion. The figures seem to be aimed at presenting as many plots as possible, such that it is challenging to find where the authors are drawing specific conclusions in the text when referring to a certain panel. The main analyses of facial shape-associated SNPs are based on a very outdated GWAS, and the sections regarding positional codes are written without any reference to the significant knowledge on positional codes from the mouse. Finally, the narrative of the paper seems to follow the order of data generation (scRNA-seq, spatial transcriptomics, ATAC-seq), rather than centering around addressing a series of points or questions.

My overall feedback would be to significantly reorganize the manuscript and figures such that there is first a description of all the data collected, and then a handful of more in-depth vignettes illustrating the utility of the data by analyzing this rich resource in light of known craniofacial biology.

Given these large suggested changes, I do not believe it is useful to comment on every single issue in the current manuscript, however here are several more specific issues in the current manuscript:
- The number of individual samples used is not clear. I am fairly certain that for a given timepoint and data modality, samples from only one individual were used, and that this is a different individual between data modalities – is this actually the case? The authors should make this clear.
- There are multiple issues with the analysis of genes nearby facial shape-associated SNPs. The list of facial shape-associated SNPs is from a 2016 paper and is very out of date. The authors also use very relaxed and varying p-value cutoffs for calling significant SNPs (10^-5 to 10^-7) which are not standard. There have been numerous subsequent GWAS of facial shape, identifying many more SNPs. They are summarized in the recent review, Naqvi & Hoskens ARGHG 2022, which also has a summary list of SNPs the authors could download and use instead.
- Also regarding the facial shape GWAS sections, there is no mention of developmental time, which is a unique aspect of this study – do genes near GWAS SNPs tend to be more highly expressed at specific timepoints during craniofacial development?
- I was surprised that the authors noted that there was not a significant overlap between facial shape-associated SNPs and accessible chromatin regions detected, but I could not find the numbers for this. It would be clarifying and helpful if the authors could provide specific fractions, i.e. what fraction of GWAS SNPs (and those in LD) overlap an accessible region in their ATAC-seq.
- The section on microCT (183-196) seems very out of place, I do not see how this belongs in a section about facial shape variation and GWAS.
- Finally, the conclusion section for the facial shape GWAS section do not seem well-supported by the data presented. While it is true that genes near facial shape GWAS signals seem to be more highly expressed in cartilage versus bone (although 'bone' is never actually labeled on any of the UMAPs), the majority of craniofacial syndrome-related genes (58%) apparently fall into a 'bone-related' cluster, with only a small fraction being cartilage related.
- The three sections on positional codes are quite confusing and do not actually discuss what any of these regional/positional codes are and whether they are the same or different to those found in model organisms (i.e. the Alx transcription factors pattern various parts of the upper face/FNP, whereas the Dlx transcription factors pattern nested portions of the lower face). I note that in Figures 7 D,E, ALX1/4 and DLX5 are in fact some of the most specifically regionally enriched genes, but whether these genes are driving the patterning could, for example, be tested by motif enrichment analysis of the

region-specific ATAC-seq peaks.
- The link to the Github code provided is broken, and there is no mention of microCT data availability. Since this paper is being presented as a resource, it is essential to make these data easily accessible to the community, with good documentation.
- The discussion has some sections which border on wild speculation/have inaccurate claims. The section on connecting grandchildren-likeness (lines 496-506) to expression of imprinted genes (which were barely mentioned in the results section) is very far-fetched.
- In the next section (lines 507-515), the authors claim that there is not significant overlap between loci associated with human facial shape variation and genes causing craniofacial syndrome. This is not true, and is highlighted both in the various recent facial shape GWAS and the review by Naqvi & Hoskens, ARGHG 2022.

Reviewer #3:
Remarks to the Author:
In this paper, the authors created a multi-modal atlas across several key developmental stages (from the sixth to the twefth week) of the human face. They performed combined analyses using their new dataset and other published data to characterize how different cell types in the developing human face contribute to facial variation and pathology. The single-cell atlas of human craniofacial development they produced highlights interesting patterns in the transcriptional profiles of facial mesenchyme. Analysis of the the single cell atlas jointly with published GWAS data on SNPs associated with 19 distinct facial traits indicated cartilage cells' associations with traits in early development and bone deposition during mid-gestation. A spatial transcriptomic atlas revealed positional information about gene expression, and predictions of cell-cell signaling landscapes were made using visium. The analysis revealed information about pathways including Midkine, IGF, WNT, and others across multiple facial regions during human craniofacial development. Furthermore, using the spatial and single-cell datasets, region-specific hierarchies of skeletal cell fates were discovered, such as shared progenitors for nasal chondrocytes and olfactory mesenchyme. Single-nuclei multi-omic gene expression assays suggested that enhancer activity might not account for the predominant effects of noncoding SNPs on gene expression in facial tissues. Finally, analysis of chondrocyte single-cell data revealed unique transcriptional identities for cartilage from different anatomical regions, reflecting developmental origins.
This paper provides an important multi-modal atlas for the field, and it advances our knowledge about how cell type positional codes and enhancers contribute to human facial individuality and pathology. A few suggestions below:
- In results section 1, to understand the positional origin of the analyzed cells, the authors performed 130 microdissections of the face samples into distinct regions for separate analysis via scRNAseq. In Figure 1B, the authors showed the regions of interest, however, they did not explain how they made sure that each sample was accurately and exclusively dissected from the region of interest. Given the very tiny size of the regions and how close the regions of interest are to each other, it would be useful for the reader to understand how cells from one region of interest are obtained without being contaminated by cells from the adjacent regions.
- In results section 2, the authors built a model of the cell types involved in the different traits of human face and for this they used a speculative assumption that SNPs from GWAS are potential enhancers of all genes close by (<500kb), and with that they concluded that the cartilage cell type is heavily involved in facial traits. However, in section 6, by using a less speculative way to find enhancers (Multiome and CAGE), they did not find a major association between enhancers and SNPs. The authors should reconcile these seemingly contradictory approaches.
- In results section 2, based on microCT analysis comparing the human vs mice facial development, the authors suggested that larger mammals might rely on accelerated facial bone growth. However, it would seem that a larger sampling would be needed to make such a suggestion.
- For the visium assay performed in results section 3, the authors only use 3 samples of different timing and region (a week 8 frontal section nasomaxillary, a week 9 sagittal section of mostly lateral periocular tissue, and a week 11 sagittal section). The sample size is a concern and the data obtained from the samples may not be representative; this should be addressed.
- Overall, the paper would benefit from some reorganization/rewording to facilitate the reading. Instead of organizing the manuscript by experiment, it might be easier to do it by question/concept. Section 6 for example might be divided in 2 (1/ enhancers and SNPs/facial traits vs 2/ enhancers as potential regulators of cell type specification). Moreover, there is a good amount of text that seems unnecessary when the description of the actual data is relatively light, especially for the end of section 6 and section 7 where it is hard to follow the link between the text and the actual figures. In addition, the panel letters do not fully correspond between the text and the figures (for figures 6 and 7) increasing the difficulty to follow.
Minor comments:
- Figure 1a is not consistent with the text, given the manuscript states the samples were from 6 weeks to 12 weeks, but the schematics shows the samples were between 6.5 weeks to 11.5 weeks.
- The complete forms of each abbrevation should be presented before it appears for the first time in the manuscript (such as line 128, GE/ATAC seq, CAGE, etc.)
- It looks like it was the middle aged rather than the youngest cell clusters that were rated as more progenitor-like based on Figure 1h.
- The description of cells were not consistent between Figure 2B and C. For example, the cell populations in B described as ‘hematopoietic’, ‘chondrocyte’, and ‘neuronal’, were as ‘blood’, ‘cartilage’, and ‘neuron’ respectively.
- Line 287 ‘…according to the maturation stages of observed transcriptional trajectories (Figure 5B)’. It should be describing Figure 5C.
- Line 310, the content is describing Figure 5U but the authors did not refer.
- Line 318 needs rewording. It is not “the enhancers and their TADs that are extruding loops”, but it is the loop extrusion that is creating TADs and then allowing enhancers to connect to promoters even if they are separated by many kilobases.
- Line 321-322, ‘have been discovered by comparing against mouse and chimp neural crest cells’. The authors should cite

the papers reporting mouse and chimp neural crest cells.
- Line 431 '…as many represented general face regional markers (Figure 7Q). However, there is no panel Q in Figure 7.

Version 1:

Decision Letter:

Our ref: NG-A64573R

27th Jan 2026

Dear Dr. Adameyko,

Thank you for submitting your revised manuscript "Cell types, positional codes, and enhancers contributing to human facial individuality and pathology" (NG-A64573R). It has now been seen by the original referees and their comments are below. The reviewers find that the paper has improved in revision, and therefore we'll be happy in principle to publish it in Nature Genetics, pending minor revisions to satisfy the referees' final requests and to comply with our editorial and formatting guidelines.

If the current version of your manuscript is in a PDF format, please email us a copy of the file in an editable format (Microsoft Word or LaTex)-- we can not proceed with PDFs at this stage.

We are now performing detailed checks on your paper and will send you a checklist detailing our editorial and formatting requirements soon. Please do not upload the final materials and make any revisions until you receive this additional information from us.

Thank you again for your interest in Nature Genetics Please do not hesitate to contact me if you have any questions.

Sincerely,

Margot Brandt, PhD
Senior Editor
Nature Genetics
https://orcid.org/0000-0002-9434-794X

Reviewer #1 (Remarks to the Author):

I am happy with the revision and I am looking forward to working with the data as soon as possible.

Reviewer #1 (Remarks on code availability):

The readme file should be improved.

Reviewer #3 (Remarks to the Author):

The authors have performed an extensive and thoughtful revision and carefully addressed the points raised in the previous review. The manuscript has clearly improved in clarity, data depth, and biological cohesion. Just a few additional thoughts:
1) In response to my first question regarding sampling strategy and potential cross-contamination issue for scRNAseq, the authors provide substantially more detail regarding their dissection approach. The use of spatial transcriptomics as a validation is also a strong point. Mapping region-specific signatures back onto tissue sections is exactly the type of cross-check one would expect in a spatial study.
2) In response to my original concern on the contradiction in enhancer inference between early results vs later sections. The authors explained that the early version was limited in GWAS coverage and lacked enhancer annotation depth. In the revision, the authors provided more robust analyses as well as functional validations in mouse of the predictions related to role of PAX1 linked enhancer and of the role of specific cell types for several craniofacial traits. However, it should be more clearly stated that those analyses such as LinkPeaks correlations and enhancer overlaps represent candidate regulatory interactions, not proofs of causality, to prevent readers from over-interpreting proximity alone as regulatory evidence.
3) In the revision, the authors removed the speculative suggestion that "larger mammals rely on accelerated bone growth" and instead use the micro-CT analysis of human embryos to show how in general, early human cartilage templates the later bone formation supporting the aforementioned hierarchical (broad-strokes to fine-tuning) model of cellular contributions to face shaping. Thus, no further concerns here.
4) On the sample size concern in Visium spatial analyses, the authors now appropriately clarified that Visium was down-prioritized due to resolution limitations, and that StereoSeq now supports the main spatial conclusions. I would suggest that the authors explicitly state that Visium results are confirmatory and not the primary spatial source. This will prevent misinterpretation of sample size concerns.
5) The authors have substantially reorganized the manuscript around biological questions (positional codes, enhancer regulation, functional examples) rather than sequencing or modality. This addresses my main structural concern.

Overall, the authors have meaningfully improved the manuscript and have responded constructively to reviewer feedback. I recommend acceptance pending minor revisions.

Open Access This Peer Review File is licensed under a Creative Commons Attribution 4.0 International License, which permits use, sharing, adaptation, distribution and reproduction in any medium or format, as long as you give appropriate credit to the original author(s) and the source, provide a link to the Creative Commons license, and indicate if changes were made.
In cases where reviewers are anonymous, credit should be given to 'Anonymous Referee' and the source.
The images or other third party material in this Peer Review File are included in the article's Creative Commons license, unless indicated otherwise in a credit line to the material. If material is not included in the article's Creative Commons license and your intended use is not permitted by statutory regulation or exceeds the permitted use, you will need to obtain permission directly from the copyright holder.
To view a copy of this license, visit https://creativecommons.org/licenses/by/4.0/

Referee expertise:

Referee #1: bioinformatics; craniofacial disorders.

Referee #2: craniofacial genetics; human genetics; single-cell genomics

Referee #3: tissue and organ development, including craniofacial

Reviewers' Comments:

We sincerely thank all reviewers for their thorough and constructive comments, which greatly contributed to improving the quality and depth of our study over the course of the 20-month revision process. Guided by your valuable feedback, we concentrated our efforts on expanding the dataset and performing a more comprehensive and rigorous analysis. We hope the reviewers will recognize that the manuscript has been extensively reorganized and now presents not only a substantially enhanced and refined atlas, but also novel experimental insights demonstrating how researchers can effectively utilize the enriched multimodal human developmental dataset.

Reviewer #1:

Erickson et al. present spatial transcriptomics data at single-cell resolution for human facial microdissections at different developmental stages. This data could become an essential resource for everyone interested in the gene regulatory mechanism involved in craniofacial dysmorphism. The spatial scRNA-seq data was generated with Visium and StereoSeq, while cell types and clusters were identified by algorithms such as Tangram, Leiden, and Harmony. So far, the genetic architecture of the human face has been analyzed by genome wide association studies, as well as in medical genetics. While GWAS results point to loci in the genome that are involved in the normal variation of the face, pathogenic variants causing Mendelian disorders with facial dysmorphism are of higher effect size.

Major

1. The authors computed gene-peak pairs with correlated patterns across cell populations. I tried to run a few analyses myself for the gene sets identified by the authors and those from references 2,4,7,42,53,54 (mainly 2). I did not find any significant overlaps. Since this is not necessarily to be expected, this is not a criticism of their results. However, could you please comment on why we see so little overlap between the “links” from spatial transcriptomics and GWAS?

We thank the reviewer for this comment. The most explanatory reason for little overlap in the first submission was a lower amount of data as compared to what we have now (when it comes to the number of GWAS datasets used, multi-omics datasets used, and spatial data). Although the sensitivity of our initial analysis was lower, we recovered some reliable information at that time. For instance, the conserved peak containing a SNP linked to PAX1 (shown in our revised Figure

3) was found during the previous version of the study (Figure 6 of the initial submission). This overlap is again reproduced in our more extended analysis, and during the revision we validated the importance of this loci for skeletal development in mice. Now, the revised study includes a Supplementary Table 2 showing all overlap between GWAS studies that we checked and our linked peaks, which reveals around 800 significant face-related SNPs associated with different cell types, compared to the few that were observed before.

Based on the information provided in the question, we don't know exactly which comparisons were performed during Reviewer's independent analysis of the data, but there are a few explanations that together contribute to our improvements in finding GWAS overlaps now versus before.

1) Increase in the number of GWAS SNPs used to seek overlaps: We initially only ran overlaps based on Shaffer 2016, but during the revision we initiated collaboration with Peter Claes group to obtain access to full breadth of statistics related to data from White et al. 2021, a far more updated GWAS study than Shaffer 2016. We also re-used SNPs from Axel Visel's recent publication Rajderkar et al. 2024. By increasing the number of SNPs, we found far more overlaps in our own analysis. Importantly, some genome-wide significant variants from White et al are not shown in their publication or supplementary tables, because of the large number of genome-wide significant SNP signals recovered. So, based on which source data the reviewer used to check overlaps, some signals could have been missed.
2) Increase in the number of samples used to derive Links: during this revision, we significantly improved the depth and the quality of our analysis (increased from 6 to 11 embryos analyzed by scRNAseq, increased from 2 to 4 embryos analyzed by snRNA/ATACseq). For the first submission, we performed LinkPeaks on 3 samples from a single embryo, but for the revision, we calculated LinkPeaks based on 11 samples from 4 embryos.
3) More robust link calling: LinkPeaks previously was calculated on nose, eyes, and jaw region samples separately, whereas in the revised version, a peak dictionary from all samples was first calculated prior to running LinkPeaks on the integrated dataset. This approach is more robust to the noise intrinsic to the ATAC sequencing method. Because of this more accurate approach, we obtain more high confidence linked peaks. Also, we had previously used parameters for LinkPeaks which imposes a cutoff at a genomic distance of 500kbp, which we increased during this revision to 3Mbp. We noticed per gene, the average distance between that gene and its many linked peaks was 1Mbp. Therefore, it may be that some significant GWAS overlap was previously missed because of the distance thresholding approach taken.
4) We doubled the number of spatial transcriptomics samples used to calculate Moran's I genes, resulting in more spatially autocorrelating genes, and more confidence in those that were picked up across both samples. If the reviewer was referring to overlap between gene lists only, it should be noted that we found many overlaps with genes implicated in the White et al. GWAS study. It should be noted that some key overlaps between the spatial gene bodies and White et al. GWAS might be missed due to congenital malformations: spatial genes appear to be highly important for facial development and

cause pathology when mutated (Fig. 2c, Supp. Fig. 18a-b). It may be that mutations in the gene body itself are more likely to result in dramatic alterations, rather than facial individuality along a rather "normal" spectrum of morphology.

This should ameliorate the Reviewer's concerns about the proportion of links containing significant GWAS SNPs.

I also checked facial phenotypes in OMIM and the GestaltMatcher database for all the genes discussed by the authors and found a slightly higher hit rate for facial dysmorphism. Besides PAX1, I found relevant medical conditions for CCDC8, TGFBR2, TBX3, LTBP4, and ALX4. However, I am not sure, whether I am able to make any sense of the abnormal medical phenotype and the spatial scRNA data. I was looking for the genes in the signatures of the Leiden clusters and the spatial information and expected to find genes linked to a facial abnormality in close proximity. Could you comment on how you think the atlas can be used in medical genetics?

We agree with the Reviewer and thought about how to orient this study better towards medical genetics. To directly answer the question, we write in our revised Discussion how we envision medical geneticists using the atlas: *"These results provide a foundation for the identification of novel enhanceropathies from unsolved craniofacial anomalies lacking coding mutations."*

We agree that the previous analysis was a bit superficial when it comes to disease, and this was because we were originally mostly focused on variation – however, during the revision we became convinced that we identified a subset of genes that can partly explain the connection between disease and variation, so we focused on regulation of these genes which appear important for both. We explain this below:

1. The addition of more samples to the spatial, snRNAseq, and scRNAseq atlas help us prioritize with greater confidence the disease-related genes that we focus on. We decided to go deeper into the analysis 'spatial genes' list because we found them to be deeply involved in craniofacial pathologies based on two approaches: first, Enrichr analysis (Revised Figure 2c and Supplementary Table), as well as statistical test of gene list overlaps between spatial and disease genes derived from HPO searches (over 3000 genes associated with diseases of head and neck collected via HP:0000152 and all descendants, Revised Supplementary Fig. 18 and Supplementary Table). After identifying these key genes, we attempt to find their upstream regulatory elements using LinkPeaks, and overlap those with GWAS to find those enhancers which appear relevant to human facial shape.
2. The remainder of the core of the manuscript (heavily revised Figure 3, 4, 5) is centered on providing examples of characterizing the roles of regulatory sequences for genes which show up in our spatial autocorrelation screen as marking a specific spatial module, but which are also strongly disease-associated, that are highly expressed in mesenchymal progenitor cells, and are linked to peaks containing SNPs associated with facial variation. We also directly test the overlap between the HPO-derived disease genes, and genes which are linked to peaks containing SNPs associated with normal facial variation (Supplementary Figure 18 and Supplementary Table 5).

3. We expanded on the cellular distribution of disease genes in facial cells during development by a heatmap for over 200 selected disease genes (Supp. Fig. 19) which was curated with the help of the review article by Naqvi et al 2022. Formatting this data as a heatmap gives far more detail into cell-type specificity of key genes than showing pooled gene signatures on UMAP, the approach taken during our initial submission. We hope the reviewer finds this new format informative.

4. We now dedicate a figure to cleft palate-related gene expression in the epithelial cells which suggests pleiotropic and epithelial-specific enhancer activation of multiple genes including key cleft-associated gene IRF6 (revised Figure 6).
5. Finally, by improving our LinkPeaks analysis and overlapping it with more known enhancer signatures (based on epigenomic atlas from Justin Cotney's lab and CHIPseq data from Axel Visel's lab), we made the LinkPeaks results much more useful for medical genetics (revised Supplementary Figure 13, revised Supplementary Table 2, and examples in panels from revised Figures 3, 4, 5, and 6). This is a necessary screen that paves the way to future studies to prove that these enhancers actually drive gene expression and are implicated in disease for which a protein-coding mutation cannot be found. Thus, having this list of candidate interactions should eventually lead to useful information for genetic consultancy.

Minor
In line 496f you state that grandchildren share a stronger resemblance with their grandparents. I doubt it. Do you have a reference?

- This was taken from the previous discussions about the observations that sometimes grandchildren resemble grandparents more than parents (https://www.newsweek.com/grandparents-prove-genetics-skip-generation-2025095 and other media reports about such phenomena). Partly, this can be explained via imprinted genes influencing facial development, or other reasons including a concentrated genetic fraction of alleles (doi: 10.1534/genetics.120.303680). However, we did not find more reliable scientific literature source with morphometry and reliable quantifications (also comparing grandparents and parents), and therefore we completely abandoned this line in the revised manuscript. We thank the reviewer for pointing this out.

Reviewer #2:

This paper describes a multimodal atlas comprising scRNA-seq, multimodal scRNA + ATAC-seq, spatial transcriptomics, and microCT of the developing human face. The stated goal of the paper is to identify the specific cell types and spatiotemporal gene expression patterns that give rise to both normal-range and disease-associated human craniofacial variation. To do this, the authors map the expression of genes and candidate enhancers near SNPs associated with facial

variation, predict cell-cell signaling interactions, and identify positional variation in gene expression and chromatin accessibility in mesenchymal and cartilage cells. They conclude that this atlas has and will provide significant utility in mechanistically understanding how the human face is formed.

Overall, I believe that the data described in this paper will be a significant resource for understanding human craniofacial development and its variation at a more granular and mechanistic level than has previously been possible. This type of in-depth molecular characterization of early human craniofacial development does not exist elsewhere. It seems that the quality of the data themselves are reasonably high, and that the rest of the field should be able to use them in interesting and impactful ways.

- We thank the reviewer for valuing our atlas part. In this revision, we focused on deepening and improving the datasets, so the atlas became more complete. For this, we

1) added new scRNAseq data replicates

2) also added new snATAC/RNAseq replicates and improved the LinkPeaks analysis

3) introduced new Stereoseq replicates and improved analysis of spatial codes

4) provided more CAGE data and improved the corresponding analysis

5) we introduced and re-analyzed published HiC and ChipSeq data for enhancer characterization of links to generate the better understanding of regulation in developing human face

We hope the reviewer appreciate this development during this review, as we almost doubled the atlas content, also to enable a deeper analysis and make possible the expanded discovery part, which now includes:

6) a hierarchical structure of cell contributions to human facial individuality (diminishing over time/differentiation) - with a great impact from mesenchymal skeletogenic populations. In general, we performed deeper analysis of cell populations from human face overlapping with expressed genes and accessible chromatin regions corresponding to human facial variation polymorphisms (via GWAS integration).

7) based on the predictions from point 6, we performed a functional validation of an unexpected predicted connection between peripheral facial nerves and the facial shape (via analysis of Ngn1 knockout mouse embryos and postnatal animals)

8) Using expanded spatial transcriptomics, we predicted which spatial genes are patterning facial mesenchymal cells in a combinatorial way

9) Based on such predictions, we discovered a new GWAS-linked enhancer for Pax1 (Pax1-cCRE), which we targeted in a mouse model to test facial shape change.

10) We characterized overlap between regulatory regions involved in disease and facial shape variation, and we generally put a lot more emphasis on disease genes and their regulatory regions, especially those, which are also involved in human facial variation.

11) We also revealed that enhancers of disease genes with facial shape variation-related SNPs show increased heterozygosity, and that some introgressed from Neanderthals into modern humans.

However, the presentation of the data and associated narrative in its current form do not demonstrate this utility in a coherent fashion. The figures seem to be aimed at presenting as many plots as possible, such that it is challenging to find where the authors are drawing specific conclusions in the text when referring to a certain panel. The main analyses of facial shape-associated SNPs are based on a very outdated GWAS, and the sections regarding positional codes are written without any reference to the significant knowledge on positional codes from the mouse. Finally, the narrative of the paper seems to follow the order of data generation (scRNA-seq, spatial transcriptomics, ATAC-seq), rather than centering around addressing a series of points or questions. My overall feedback would be to significantly reorganize the manuscript and figures such that there is first a description of all the data collected, and then a handful of more in-depth vignettes illustrating the utility of the data by analyzing this rich resource in light of known craniofacial biology.

- We thank the reviewer for this useful comment. This is exactly what we have tried to do for the revision. Thank you for the great suggestion. Now the structure is totally different from the initial submission, as we will explain now:

As suggested by the reviewer, we begin by providing an overview of all the data collected during the study (revised Figure 1). After this, because of the substantial transcriptional heterogeneity in the mesenchymal cells correlating with cell location, we focus on identifying and characterizing genes which may be responsible for spatial patterning of human mesenchymal cells using the unbiased screening approach (revised Figure 2). It turns out that these genes are highly involved in facial development in mice, and are enriched in human disease. Because of their key importance, and because enhancers spatiotemporally control genes, we spend the next section identifying enhancers in order to learn more about the regulation of these spatial genes (introduced in revised Figure 3 and revised Supplementary Figure 13). At this point, we find out that the enhancers of spatial genes contain many SNPs associated with facial variation, and that their expression profile in embryonic cells can even partly explain why certain SNPs are associated with specific facial traits and not others (revised Figure 4); here we get to the 'who' (cellular basis), 'when' (age), and 'where' (trait/region), of the influence of spatial genes on facial shaping.

The latter part of the manuscript are specific vignettes centered around spatiotemporal regulation of specific genes by enhancers in human embryos. The selected genes all have the following characteristics: 1) selected from the list of spatial patterning transcription factors 2) are responsible for congenital malformations, 3) differentially expressed across facial regional domains, 4) differentially expressed across mesenchymal cell clusters, 5) are linked to peaks overlapping enhancers and containing variation SNPs.

We also added more discussion about the known roles of some positional codes in the mouse to the Discussion:

“A central challenge has been deciphering how the anterior face encodes positional identity. Unlike the trunk and hindbrain, which rely on Hox codes, the upper and midface develop without a canonical Hox system. Only a handful of factors such as ALX, DLX, MSX, and endothelin signaling were previously known to act regionally based on studies in mice. However, high-resolution profiling had not yet been performed to generate a comprehensive list of candidate human facial patterning regulators. Using unbiased spatial clustering approaches [92], applied to highly resolved spatial transcriptomics data, we identified hundreds of ‘spatial’ genes, including dozens of secreted factors and TFs. In addition to the above-mentioned factors, the gene list includes established patterning TFs with proposed spatial codes in vertebrates, such as PAX domains along the anterior-posterior axis of cranial neuroectoderm[93], and FOX domains that contribute to diversification of ectomesenchyme in pharyngeal arches[94]. In addition, we identified spatial TFs that have not been previously associated with roles in patterning, such as ZNF503 and ZNF804a, as well as patterning factors that have not been implicated in craniofacial development, such as PROX1 and HES6.”

Given these large suggested changes, I do not believe it is useful to comment on every single issue in the current manuscript, however <u>here are several more specific issues in the current manuscript:</u>

The number of individual samples used is not clear. I am fairly certain that for a given timepoint and data modality, samples from only one individual were used, and that this is a different individual between data modalities – is this actually the case? The authors should make this clear.

- We apologize that it was not clear in the previously submitted version, but now we have increased the dataset size substantially and added biological replicates. The revised Figure 1a-b now summarizes all of the replication in the study (number of embryos used for each modality at different time points).

There are multiple issues with the analysis of genes nearby facial shape-associated SNPs. The list of facial shape-associated SNPs is from a 2016 paper and is very out of date. The authors also use very relaxed and varying p-value cutoffs for calling significant SNPs (10^-5 to 10^-7) which are not standard. There have been numerous subsequent GWAS of facial shape, identifying many more SNPs. They are summarized in the recent review, Naqvi & Hoskens ARGHG 2022, which also has a summary list of SNPs the authors could download and use instead.

- We agree with the Reviewer, and we took several steps to improve and respond to this comment. Firstly, we established a collaboration with human facial GWAS experts - Peter Claes lab to make sure we understand and use the most complete GWAS lists. Peter kindly agreed to join our team and is among our co-authors in the revised manuscript.

Secondly, we took advantage of the recently published GWAS lists from 2024 (Dynamic Enhancer Landscapes etc. from Axel Visel’s group) and initiated collaboration with the authors of White et al 2021 (Insights into genetic architecture of human facial shape) to receive the detailed p-values

for all segments for each of the lead SNPs from that more updated GWAS study. This allowed us to re-do the analysis of cellular contributions to traits (previously Figure 2, now revised Figure 4) with a higher threshold (around 10^-8.43) for the main result. This also facilitated directly comparing p values across traits to assess trait-specificity and subsequent comparison to gene expression region-specificity. We still use Shaffer SNPs for some visuals, because the PAX1-cCRE was born from an observation of overlap with a significant Shaffer SNP, but in most cases, we rely on SNPs from White in the revised study.

Also regarding the facial shape GWAS sections, there is no mention of developmental time, which is a unique aspect of this study – do genes near GWAS SNPs tend to be more highly expressed at specific timepoints during craniofacial development?

- We thank the reviewer for this question, and we paid attention to this during the revision. We calculated the average age of each cell cluster and show that age negatively correlates with CYTOTRACE score (proxy for cell progenitor-like state) and that age leads to the emergence of definitive cell types (revised Figure 1f, revised Supplementary Figure 2) and using this information, we were able to estimate a diminishing effect of cellular gene expression state on facial diversity over time (significantly enriched populations shown in revised Figure 4, see revised Supp. Fig. 16 for full dataset). Early clusters with young average age were significantly enriched for traits across the board, with more differentiated cells having little to no significant enrichments and generally being ranked lower (revised Supplementary Figure 16).

I was surprised that the authors noted that there was not a significant overlap between facial shape-associated SNPs and accessible chromatin regions detected, but I could not find the numbers for this. It would be clarifying and helpful if the authors could provide specific fractions, i.e. what fraction of GWAS SNPs (and those in LD) overlap an accessible region in their ATAC-seq.

We include specific numbers in the main text and added an integrated table now in Supplementary Table 2. The SNPs come from a number of GWAS studies but were found to be in LD of lead SNPs by Axel Visel's team in 2024 publication etc.

The section on microCT (183-196) seems very out of place, I do not see how this belongs in a section about facial shape variation and GWAS.

The reasoning for including these data is partly that our results point to a hierarchical model in which, over time, there is a diminishing effect of cellular gene expression on facial variation. Accordingly, we want to show what these evolving tissue shapes actually look like in practice:

> *"In humans, as well as other vertebrates such as mice, musculoskeletal tissue shapes are generally established early and appear to faithfully scale up during development, as indicated from micro-CT scans (Supp. Fig. 4 and 15)."*

We introduce the microCT data earlier and hold back the GWAS until relatively later in the manuscript to improve the flow.

- Finally, the conclusion section for the facial shape GWAS section does not seem well-supported by the data presented. While it is true that genes near facial shape GWAS signals seem to be more highly expressed in cartilage versus bone (although ‘bone’ is never actually labeled on any of the UMAPs), the majority of craniofacial syndrome-related genes (58%) apparently fall into a ‘bone-related’ cluster, with only a small fraction being cartilage related.

-Our analysis of GWAS has improved substantially since the previous submission. The use of lower significance GWAS signals for overlap in the initial submission introduced noise which we improved by utilizing the highly significant GWAS results from White et al 2021, during this revision. The addition of more scRNAseq data also increased our confidence in the cell types retrieved (bone cells sometimes hid internally in the mesenchymal supercluster in earlier versions of the dataset).

Further, instead of the manual pattern-based classification of the disease genes presented in the original version, we decided to show the results as a hierarchically clustered heatmap, where the distribution across cell types of every disease gene can be independently assessed (Supplementary Figure 19). In general, our new results suggest that as early as we can detect, cells are highly expressing genes near GWAS signals, and this SNP-adjacent expression gets exchanged for expression of other genes over the course of cell maturation and time.

In addition, we overall decided to focus more intensely on what our atlas may facilitate learning about the regulation of certain known pathologies involving patterning genes (revised Figs 3-5), as well as orofacial clefts (revised Fig 6) in accordance with the reviewer suggestions about manuscript organization.

- The three sections on positional codes are quite confusing and do not actually discuss what any of these regional/positional codes are and whether they are the same or different to those found in model organisms (i.e. the Alx transcription factors pattern various parts of the upper face/FNP, whereas the Dlx transcription factors pattern nested portions of the lower face). I note that in Figures 7 D,E, ALX1/4 and DLX5 are in fact some of the most specifically regionally enriched genes, but whether these genes are driving the patterning could, for example, be tested by motif enrichment analysis of the region-specific ATAC-seq peaks.

- We took this suggestion seriously and performed SCENIC+ analysis for eRegulons, as well as analyzed Transcription Factor Binding Site motif enrichment across cell populations for the spatial genes. These results suggest that the genes are indeed active during patterning of mesenchymal cells in the human embryos too. Also, it may have been an oversight that we did not specify the known roles about these genes but we hope that it is now much clearer from the revised text that the ALX1 and DLX5 factors appear to behave very similarly in human as in mouse, by all accounts (revised Figure 2d-g, revised Figure 4c-e).

- The link to the Github code provided is broken, and there is no mention of microCT data availability. Since this paper is being presented as a resource, it is essential to make these data easily accessible to the community, with good documentation.

- These issues are in progress of being fixed, we totally agree that by the time of publication, community access to the full data will be essential. We are still struggling with GEO (because of the interruptions in the US government funding), but we uploaded the count matrices of our sequencing data to Dryad.

For an interactive viewing of the atlas, we have made the data available on CELLXGENE.

Raw reads will be available at the Karolinska Depository via the DORIS database prior to publication.

MicroCT scans are submitted to Zenodo.

All links can be found in the Data Availability Statement in the manuscript (some DOIs pending).

- The discussion has some sections which border on wild speculation/have inaccurate claims. The section on connecting grandchildren-likeness (lines 496-506) to expression of imprinted genes (which were barely mentioned in the results section) is very far-fetched.

- We agree with the Reviewer, and we have removed these lines and the corresponding logic from the revised discussion. We hope the revised version is now more grounded.

At the same time, because imprinted genes are known to influence craniofacial anomalies, we felt it was important to retain a brief overview of their expression in our dataset (revised Supplementary Figure 20). While we cannot assess allele-specificity with the current 10X platform, we believe it is valuable to show how numerous imprinted genes are expressed in human embryonic craniofacial cells, and we present this as a caveat and direction for future studies rather than a central claim.

- In the next section (lines 507-515), the authors claim that there is not significant overlap between loci associated with human facial shape variation and genes causing craniofacial syndrome. This is not true, and is highlighted both in the various recent facial shape GWAS and the review by Naqvi & Hoskens, ARGHG 2022.

- We thank the Reviewer for pointing this out. After re-analyzing additional GWAS datasets with far more stringent SNP sets (e.g., White et al.) and expanding our disease gene list using the HPO database, we indeed find significant overlap between genes located near loci associated with normal facial variation and genes implicated in craniofacial syndromes. We have updated the relevant section of the manuscript to reflect this corrected interpretation. In the revised text (revised Supplementary Figure 18), we now highlight that this overlap is partly driven by genes linked to peaks harboring variation SNPs as well as by spatially autocorrelated genes. These findings suggest that variation SNPs may alter enhancer sequences regulating disease genes, consistent with the Reviewer’s comment and with the aforementioned review.

<u>Reviewer #3:</u>

In this paper, the authors created a multi-modal atlas across several key developmental stages (from the sixth to the twelfth week) of the human face. They performed combined analyses using their new dataset and other published data to characterize how different cell types in the developing human face contribute to facial variation and pathology. The single-cell atlas of human craniofacial development they produced highlights interesting patterns in the transcriptional profiles of facial mesenchyme. Analysis of the single cell atlas jointly with published GWAS data on SNPs associated with 19 distinct facial traits indicated cartilage cells' associations with traits in early development and bone deposition during mid-gestation. A spatial transcriptomic atlas revealed positional information about gene expression, and predictions of cell-cell signaling landscapes were made using visium. The analysis revealed information about pathways including Midkine, IGF, WNT, and others across multiple facial regions during human craniofacial development. Furthermore, using the spatial and single-cell datasets, region-specific hierarchies of skeletal cell fates were discovered, such as shared progenitors for nasal chondrocytes and olfactory mesenchyme. Single-nuclei multi-omic gene expression assays suggested that enhancer activity might not account for the predominant effects of noncoding SNPs on gene expression in facial tissues. Finally, analysis of chondrocyte single-cell data revealed unique transcriptional identities for cartilage from different anatomical regions, reflecting developmental origins. This paper provides an important multi-modal atlas for the field, and it advances our knowledge about how cell type positional codes and enhancers contribute to human facial individuality and pathology.

<u>A few suggestions below:</u>

Major comments

In results section 1, to understand the positional origin of the analyzed cells, the authors performed microdissections of the face samples into distinct regions for separate analysis via scRNAseq. In Figure 1B, the authors showed the regions of interest, however, they did not explain how they made sure that each sample was accurately and exclusively dissected from the region of interest. Given the very tiny size of the regions and how close the regions of interest are to each other, it would be useful for the reader to understand how cells from one region of interest are obtained without being contaminated by cells from the adjacent regions.

We understand this concern of the reviewer, and we hope we can clearly respond to the request. Below we provide the report about how we designed the sampling strategy to avoid cross-contaminations of different regions, and how we controlled for that using an orthogonal approaches such as spatial transcriptomics or GWAS overlaps. On note, the revised Figure 1 highlights the regions that we sampled. We would like to avoid including example photographs of the dissected regions in Supplementary data unless the reviewer explicitly requests, as we felt it might be unnecessarily graphic for some readers. In the revised manuscript, we wrote more detailed explanation of how we reliably obtained each region in a standardized way, in Materials and Methods:

“Whole craniofacial complex samples, and external ear samples, were received from the Human Developmental Tissue Biobank. Then, dissection of different regions was performed under a stereoscope with darkfield illumination to enrich samples for cells originating from a specific facial region. To achieve standardized and consistent dissection, we developed a uniform approach based on anatomical landmarks: mandibular prominence was isolated by an incision from the oral commissure to the temporomandibular joint, with connecting soft tissue removed to separate the lower jaw and tongue from the upper face. Frontonasal prominence region was defined by excising protruding nasal tissue between alar-facial grooves. Forehead region was obtained by removing flaps of cranial tissue superior to the eyes and nasion. The eye region consisted of the globes with surrounding orbital mesenchyme and adjacent maxillary tissue. For one PCW6 MO specimen only posterior (lower jaw and tongue) versus anterior (upper face) regions were distinguished. For Visium samples, craniofacial samples were bisected along the midline, with one half frozen for spatial analysis, and the other processed for cell isolation. This process naturally leaves margins between the dissection regions which minimizes cross-contamination. A subset of samples was processed directly without regional separation.”

Any significant contamination is unlikely because of the way the samples were dissected. Also, as we are used to fine dissections in E8-9 mouse embryos, dissections of human embryonic facial regions (on the scale of a few mm at the earliest stage of week 6, and scaling up rapidly after this) are not challenging with the help of stereomicroscopes. We assume that theoretically there might be negligible amount (on the scale of a fraction of %) of cell contamination from nearby adjacent regions, due to the continuous nature of transcriptional heterogeneity as a function of cell position. However, the bulk of the cells from a given region are quite far from the other regions. For example, only the external nose is taken for nose tissue, which is straightforward to separate in a clean way from the rest of the face. Thus, the nose samples consist almost entirely of cells protruding from the midface. The eyes, being pigmented, are quite easy to spot and use as a central landmark for that sample. The lower jaw samples could not have contamination from nose or eyes, for instance, yet it could have contamination from ear regions because there is only a small piece of tissue around the temporomandibular junction that connects the lower jaw region to the rest of the face.

Thus, any contamination would be a minimal amount and would not be enough to ruin the comparative analysis. Importantly, the spatial transcriptomic analysis was used to verify that gene expression signatures for the cell types identified within specific subregional dissections are found in the expected locations on tissue sections and vice versa (lingual mesenchyme, for instance, cross-reference revised Supplementary Figure 2 and 8). We also validated that the difference in expression of genes in the Nose versus Jaw sample positively correlates with the difference in significance of their nearby SNPs affecting Nose versus Jaw structures (revised Figure 4b-c). It is reasonable to conclude from this result that at the very least, the samples are highly enriched enough with cells from their respective regions to be considered representative of that region.

In results section 2, the authors built a model of the cell types involved in the different traits of human face and for this they used a speculative assumption that SNPs from GWAS are potential enhancers of all genes close by (<500kb), and with that they concluded that the cartilage cell type is heavily involved in facial traits. However, in section 6, by using a less speculative way to find enhancers (Multiome and CAGE), they did not find a major association between enhancers and SNPs. The authors should reconcile these seemingly contradictory approaches.

We did not use enough data to determine major associations in the first submission. CAGE is extremely reliable, but is not as sensitive as other methods that can be used to measure enhancers. Further, Multiome on its own does not measure the presence of enhancers, but rather identifies correlations between peak accessibility and gene expression that may have a number of explanations. Simultaneously, we were limited by a smaller GWAS dataset to seek overlaps in the first submission. Since we have upgraded our dataset quality on all fronts during this revision (enhancer detection via CAGE and ChIPseq approaches, more extensive GWAS via White et al 2021), we identified many associations between enhancers and SNPs (revised Supplementary Table 2).

Our previous submission relied on a small amount of CAGE data, which was increased in biological replicates, and we also followed the suggestion of another publication (Andersson, R. *et al. Nature 2014;* **507**, 455-461) to add a 100bp buffer zone to allow for better overlap because CAGE lacks sensitivity though it has high accuracy. In our revision, we also added biological n to 2 per each time point (4 individuals; 2 at week 6, and 2 at week 10-11; all split into different regional samples). We improved how we performed LinkPeaks by generating an integrated peak dictionary prior to computing the Links, and increased the distance threshold to catch more longer-range interactions. In addition, we utilized published data from histone modification profiling and H3K27ac, which, when combined with our Multiome data, led to a much better overlap between enhancers and SNPs. Also, the SNPs we were using before were much less significant than what we used for the revised manuscript. After teaming up with Peter Claes lab we were granted access to full statistics from the White et al dataset, which allowed us to find many more associations between enhancers and SNPs within our linked peaks dataset.

Our finding from GWAS data is basically a diminishing effect of cell types on facial shape variation as they differentiate and age in the embryo, with the strongest effect apparent in the earliest progenitor cells, and cartilage/bone having intermediate level of effects (ranked within the top 10-20 or so cell types out of 56), being enriched for smaller traits rather than large ones. Just to note – that GWAS captures variation in traits rather than the traits themselves, and pathological mutations would naturally be excluded from such a study and from such an analysis. Of course, cartilage and bone are critical for facial growth, as without them we cannot generate a face in the first place. However, according to our results, natural variation between humans (individuality) seems to be determined at a young cellular stage by early mesenchymal progenitors, and gets fine-tuned by the differentiated cartilage and bone cells, even in collaboration with peripheral nerves and other cell types, after the fact.

Overall, the current results are much more robust than the previous submission – and we also performed functional validations in mouse of the predictions related to role of PAX1 linked enhancer (revised Figure 3) and of the role of specific cell types for craniofacial traits (role of

peripheral nerves tested in revised Figure 7). It is worth noting that the predicted PAX1 enhancer was already identified in the original version of the manuscript.

In results section 2, based on microCT analysis comparing the human vs mice facial development, the authors suggested that larger mammals might rely on accelerated facial bone growth. However, it would seem that a larger sampling would be needed to make such a suggestion.

- We thank the reviewer for this comment. We gave it a second though and indeed, we think it was speculative, so we decided to remove this suggestion from the manuscript and instead use the micro-CT analysis of human embryos to show how in general, early human cartilage templates the later bone formation supporting the aforementioned hierarchical (broad-strokes to fine-tuning) model of cellular contributions to face shaping. We also think these 3D models of human facial development will be useful for medical students and for researchers interested in human-specific aspects of facial shaping even as a standalone resource (this is a resource/atlas paper after all). These 3D models will be useful to connect the gene expression in specific skeletogenic cell types to the facial shape formation based on the hierarchy of tissue formation and their co-dependencies (revised Supplementary Figure 4).

For the visium assay performed in results section 3, the authors only use 3 samples of different timing and region (a week 8 frontal section nasomaxillary, a week 9 sagittal section of mostly lateral periocular tissue, and a week 11 sagittal section). The sample size is a concern and the data obtained from the samples may not be representative; this should be addressed.

- We de-prioritized the Visium assay because of how much better Stereoseq data was in terms of resolution and overall quality. For Stereosesq we now have 8 sections across many facial regions from two independent embryos (as the result of this revision). We did not perform more analysis on the Visium assay just because of the lower resolution. However, the major results are consistent between Stereoseq and Visium spatial transcriptomics assays.

Overall, the paper would benefit from some reorganization/rewording to facilitate the reading. Instead of organizing the manuscript by experiment, it might be easier to do it by question/concept. Section 6 for example might be divided in 2 (1/ enhancers and SNPs/facial traits vs 2/ enhancers as potential regulators of cell type specification).

- We tried our best to fix these issues and substantially re-organized the manuscript after performing improved analysis approaches on our increased-size atlas. Now, we introduce all the data at once in Fig1, so that Fig2 can be dedicated to the spatial patterning gene set discovery, A supplementary figure is dedicated to enhancer discovery, and the Fig3-6 use a mix of spatial, single-cell, and multi-omics data to answer questions about (mostly) regulation of important disease-associated spatial mesenchymal patterning genes by enhancers. We hope the Reviewer agrees that this current organization is much more conceptual and methods-integrated.

Moreover, there is a good amount of text that seems unnecessary when the description of the actual data is relatively light, especially for the end of section 6 and section 7 where it is hard to follow the link between the text and the actual figures. In addition, the panel letters do not fully

correspond between the text and the figures (for figures 6 and 7) increasing the difficulty to follow.

- We agree with the reviewer. The revision allowed us to focus on which messages are key. Given that the manuscript has been substantially re-worked and re-written following the Reviewer advice, we would hope you give us another chance and we think the reading will be clearer and more enjoyable this time around.

Minor comments

- Figure 1a is not consistent with the text, given the manuscript states the samples were from 6 weeks to 12 weeks, but the schematics shows the samples were between 6.5 weeks to 11.5 weeks.

- All temporal inconsistencies have been checked in the new version. The reason for this is because microCT samples were harvested at Week 12, but the scRNAseq data only goes up to 11.5.

- The complete forms of each abbrevation should be presented before it appears for the first time in the manuscript (such as line 128, GE/ATAC seq, CAGE, etc.)

- Abbreviations have been checked for this in the new version.

- It looks like it was the middle aged rather than the youngest cell clusters that were rated as more progenitor-like based on Figure 1h.

- We added new data and re-calculated the average age of clusters and CYTOTRACE score per cluster (some of this data shown in Figure 1). In general, we see a strong and significant negative correlation between age and CT score, even if a few individual clusters don't exactly line up with this trend. From the text:

"Mean CYTOTRACE score, a proxy for cellular developmental potential, negatively correlated with cluster age (Pearson's product-moment correlation $r = -0.59$, $p = 2.0 \times 10^{-6}$) indicating a loss of multipotency over time (Figure 1f).

- The description of cells were not consistent between Figure 2B and C. For example, the cell populations in B described as 'hematopoietic', 'chondrocyte', and 'neuronal', were as 'blood', 'cartilage', and 'neuron' respectively.

- We made an improved effort for the revised version to discuss cell types using a consistent nomenclature.

- Line 287 '…according to the maturation stages of observed transcriptional trajectories (Figure 5B)'. It should be describing Figure 5C.

- We apologize for the mistake. We think all figure callouts are accurate now.

- Line 310, the content is describing Figure 5U but the authors did not refer.

- We apologize for the mistake. We think all figure callouts are accurate now.

- Line 318 needs rewording. It is not “the enhancers and their TADs that are extruding loops”, but it is the loop extrusion that is creating TADs and then allowing enhancers to connect to promoters even if they are separated by many kilobases.

- We fixed this nuance in the text.

- Line 321-322, ‘have been discovered by comparing against mouse and chimp neural crest cells’. The authors should cite the papers reporting mouse and chimp neural crest cells.

- We checked for this during the revision, and now all references to the previous literature are properly cited.

- Line 431 ‘…as many represented general face regional markers (Figure 7Q). However, there is no panel Q in Figure 7.

- We apologize for the mistake. We checked before final resubmission and we think all figure callouts are accurate now.

Finally, we would like to bring up to the reviewer’s attention that we significantly increased the overall amount of data in this revision, and additionally produced functional validations, which led to new discoveries:

1. **Expanded scRNA-seq datasets.** We generated and incorporated new single-cell RNA sequencing replicates, improving the robustness and reproducibility of the transcriptional landscape.
2. **Enhanced snATAC/RNA-seq integration.** We added new single-nucleus ATAC/RNA-seq replicates and refined the *LinkPeaks* analysis, thereby strengthening the connection between gene expression and chromatin accessibility profiles.
3. **Improved spatial resolution with Stereoseq.** We introduced additional Stereoseq replicates and enhanced the computational analysis of spatial gene expression codes, leading to a more precise mapping of transcriptional domains within developing facial tissues.
4. **Expanded CAGE dataset and analysis.** We included more CAGE data and refined the analytical pipeline, significantly improving the identification of transcription start sites and promoter/enhancers across cell types.
5. **Integration of chromatin conformation and epigenomic data.** We incorporated and re-analyzed published Hi-C and ChIP-seq datasets to characterize enhancer–promoter interactions. This allowed us to better understand the regulatory architecture controlling gene expression during human facial development.

We hope the reviewers appreciate the extent of this progress achieved during the review process. Overall, the atlas has nearly doubled in scope and resolution, enabling both deeper analyses and

a significantly expanded discovery component. These new analyses and experimental validations include:

6. **Hierarchical model of cellular contributions to facial individuality.** We constructed a hierarchical framework describing how contributions of distinct cell populations to human facial individuality change over time and differentiation. Our findings now specifically highlight the predominant role of mesenchymal skeletogenic populations (in addition to cartilage and bone major contributions). More broadly, we conducted a much better in-depth analysis of facial cell populations, correlating gene expression and chromatin accessibility with facial variation-associated polymorphisms identified through GWAS integration.
7. **Functional validation of predicted nerve–shape interactions.** Guided by predictions from point (6), we performed functional experiments validating an unexpected link between peripheral facial nerves and craniofacial morphology (from GWAS overlaps with gene expression and cell types). This was achieved through the analysis of *Ngn1* knockout mouse embryos and postnatal animals, confirming a regulatory contribution of the peripheral nervous system to facial shape.
8. **Identification of combinatorial spatial gene patterning.** Utilizing the expanded spatial transcriptomic datasets, we identified combinations of spatially restricted genes that cooperatively pattern facial mesenchymal cells, revealing novel mechanisms underlying region-specific identity within the face.
9. **Discovery of a new GWAS-linked enhancer for *Pax1*.** Based on predictions from the spatial analyses, we discovered a previously uncharacterized enhancer linked to *Pax1* (*Pax1-cCRE*). Functional testing of this element in a mouse model demonstrated its role in modulating facial shape, thus providing a direct connection between regulatory variation and morphological outcomes.
10. **Expanded analysis of regulatory regions associated with disease and facial variation.** We systematically characterized the overlap between enhancers implicated in disease and those associated with normal-range facial shape variation, emphasizing the shared regulatory mechanisms underlying both.
11. **Evolutionary insights into disease- and variation-linked enhancers.** We found that enhancers of disease genes carrying facial shape variation–related SNPs exhibit elevated heterozygosity and, notably, that some of these enhancers were introgressed from Neanderthals into modern humans, suggesting an evolutionary contribution to facial diversity.
